# Supplementary material for: Low rates of serious complications and further procedures following surgery for base of thumb osteoarthritis: analysis of a national cohort of 43 076 surgeries
Source: BMJ Open. 2021 Jul 7;11(7):e045614. doi: 10.1136/bmjopen-2020-045614 (PMC8264901; doi:10.1136/bmjopen-2020-045614)

Supplementary appendix Low rate of serious complications and further procedures following with BTOA surgery: analysis of national cohort of 43 076 surgeries with up to 19 years of follow up

Supplementary table 1 OPCS and ICD codes used to identify BTOA surgery

**BTOA OPCS codes**			**BTOA ICD-10 codes**
W57.2	alone		M18.0-18.9
W57.3	alone		M15.1-2
W57.8	**WITH ANY**	Z82.3	
W02.8		Z72.4	
W02.9		Z73.8	
W06.8		Z73.9	
W06.9		Z89.5	
W08.5		Z82.2	
W08.8		Z82.9	
W08.9			
W13.8			
W17.8			
W17.9			
W28.1			
W33.8			
W33.9			
W43.1			
W44.1			
W45.1			
W45.3			
W54.1			
W54.3			
W56.2			
W57.2			
W62.1			
W62.2			
W62.8			
W62.9			
W63.1			
W63.2			

W63.8		
W63.9		
W64.1		
W64.2		
W64.8		
W64.9		
W74.2		
W74.3		
W77.5		
W77.7		
W77.8		
W90.3		
W90.4		

Supplementary Table 2 Covariate codes

Covariates for BTOA	ICD-10 code
CTS	G560 (or OPCS A651)
Knee OA	M17
Generalised OA	M15; M19
Rheumatoid Arthritis	M05
Wrist/Hand fracture	S62
Oophorectomy	(OPCS Q22,Q23, Q43)

Supplementary Table 3 Complication codes for systemic complications and local complications

Complications	code
Wound dehiscence	OPCS Z82 + (S60.4 or S42.2 or S42.3 or S42.4)
Tendon repair	OPCS Z82 + T67
Wound debridement	OPCS Z82 + (T96.8 or T96.3 or T96.4)
Neurovascular injury	ICD code T81.2
Stroke	ICD I60X;I610-I619; I630-I639; I64X
Respiratory Tract Infection	ICD J12X J13, J14, J15X J180-J182 J188; J189; J22; J440 J441 J690 J691 J698 J851
Myocardial Infarction	ICD I210-I214; I219
Deep Vein Thrombosis/ Pulmonary Embolus	ICD I801-I803; I260; I269
Urinary tract infection/ acute renal failure	ICD N300 N390 N170-N172; N178 N179

Supplementary Table 4 Comparison of patients with and without laterality (baseline demographics)

	With laterality	Without laterality
**Total**	36 072	7004
**Sex**		
Male	7592 (21.05)	1365 (19.49)
Female	28475 (78.94)	5637 (80.48)
Missing	5 (0.01)	2 (0.03)
**Mean age (SD; years)**	63.04 (9.15)	63.13 (9.26)
**Charlson index**		
0	15365 (42.60)	2914 (41.60)
1	8937 (24.78)	1703 (24.31)
2	4746 (13.16)	947 (13.52)
3	2730 (7.57)	556 (7.94)
4	1372 (3.80)	287 (4.10)
>=5	760 (8.10)	166 (8.52)
missing	0	0
**IMD decile**		
Least deprived 10%	4103 (11.37)	646 (9.22)
Less deprived 10-20%	4041 (11.20)	728 (10.39)
Less deprived 20-30%	4055 (11.24)	764 (10.91)
Less deprived 30-40%	4023 (11.15)	795 (11.35)
Less deprived 40-50%	3947 (10.94)	735 (10.49)
More deprived 10-20%	2681 (7.43)	598 (8.54)
More deprived 20-30%	3125 (8.66)	673 (9.61)
More deprived 30-40%	3487 (9.67)	719 (10.27)
More deprived 40-50%	3820 (10.59)	739 (10.55)
Most deprived 10%	2464 (6.83)	575 (8.21)
Missing	326 (0.90)	32 (0.46)
**Ethnic group**		
Any white background	31107 (86.24)	6097 (87)
Any Asian background	380 (1.05)	68 (1)
Any Black background	71 (0.2)	12 (<1)
Any mixed background	51 (0.14)	9 (<1)
Chinese	19 (0.05)	<7*
Any other ethnic group	127 (0.35)	28 (<1)
Not stated	3639 (10.08)	650 (9)
Not known	678 (1.88)	139 (2)
missing	0	0
**Co variates**		
Carpal Tunnel Syndrome	3989 (11.06)	794 (11.34)
Knee Osteoarthritis	4559 (12.64)	936 (13.36)
General Osteoarthritis	7569 (20.98)	1667 (23.8)
Rheumatoid Arthritis	84 (0.23)	25 (0.36)
Wrist Fracture	206 (0.57)	43 (0.61)
Oophorectomy	1351 (3.74)	232 (3.31)

*Numbers less than 7 suppressed in line with NHS Digital disclosure control guidelines- percentages of other groups rounded to prevent secondary disclosure of data (29)

Supplementary table 5 Baseline demographics of all patients undergoing BTOA surgery

	**All surgery (N, %) (total=43 076)**	**Trapeziectomy N (%) (35 486)**	**LRTI N (%) (3028)**	**Arthroplasty N (%) (1640)**	**Arthrodesis N (%) (2027)**	**Partial Trapeziectomy N (%) (894)**
**Sex**						
Male	8957 (20.7)	6 826 (19.2)	616 (20.3)	390 (23.8)	934 (46.1)	191 (21.3)
Female	34 112 (79.1)	28 655 (80.8)	2411 (79.6)	1250 (76.2)	1092 (53.9)	704 (78.7)
Missing	7 (0.02)	5 (0.01)	1 (0.03)	0	1 (0.05)	0
**Mean age (SD; years)**	63.2 (SD 9.2)	63.6 (SD 8.8)	62.5 (SD 9.7)	61.7 (SD 9.3)	58.1 (11.7)	63.1 (9.8)
**Charlson index**						
0	18 279 (42.4)	14 992 (42.3)	1 324 (43.8)	677 (41.3)	890 (43.9)	396 (44.3)
1	10 640 (24.7)	8 807 (24.8)	721 (23.8)	444 (27.1)	475 (23.4)	193 (21.6)
2	5 693 (13.2)	4 746 (13.4)	392 (13.0)	199 (12.1)	250 (12.3)	106 (11.8)
3	3 286 (7.6)	2 723 (7.7)	222 (7.3)	125 (7.6)	142 (7.0)	74 (8.3)
4	1 659 (3.9)	1 359 (3.8)	123 (4.1)	60 (3.7)	77 (3.8)	40 (4.5)
>=5	3 519 (8.2)	2 859 (8.0)	246 (8.1)	135 (8.2)	193 (9.6)	86 (8.5)
missing	0	0	0	0	0	
**IMD decile**						
Least deprived 10%	4 749 (11.0)	4 015 (11.3)	268 (8.8)	203 (12.4)	153 (7.6)	110 (12)
Less deprived 10-20%	4 769 (11.1)	3 934 (11.1)	314 (10.4)	232 (14.15)	195 (9.6)	94 (10)
Less deprived 20-30%	4 819 (11.2)	3 976 (11.2)	354 (11. 5)	185 (11.3)	212 (10.5)	92 (10)
Less deprived 30-40%	4 818 (11.2)	3 974 (11.2)	393 (13.0)	157 (9.6)	212 (10.5)	82 (9)
Less deprived 40-50%	4 682 (10.9)	3 853 (10.9)	345 (11.4)	154 (9.4)	221 (10.9)	109 (12)
More deprived 10-20%	3 279 (7.61)	2 700 (7.61)	218 (7.2)	110 (6.7)	181 (8.9)	70 (7)
More deprived 20-30%	3 798 (8.8)	3 108 (8.8)	273 (9.0)	145 (8.8)	202 (10.0)	70 (7)
More deprived 30-40%	4 206 (9.8)	3 442 (9.7)	303 (10.1)	164 (10.0)	215 (10.6)	82 (9)
More deprived 40-50%	4 559 (10.6)	3 700 (10.4)	354 (11.7)	172 (10.5)	241 (11.9)	92 (10)
Most deprived 10%	3039 (7.1)	2 529 (7.1)	160 (5.3)	110 (6.7)	152 (7.5)	88 (9)
Missing	358 (0.8)	255 (0.7)	46 (1.5)	8 (0.5)	43 (2.1)	*<7**
**Ethnic group**						
Any white background	37 204 (86.4)	30 738 (86.6)	2628 (86.8)	1410 (86.0)	1640 (80.9)	755 (84.4)
Any Asian background	439 (1.0)	376 (1.1)	24 (0.8)	20 (1.2)	16 (0.8)	12 (1.3)
Any Black background	92 (0.2)	60 (0.2)	*<7**	*<7**	12 (0.6)	*<7**
Any mixed background	60 (0.1)	46 (0.1)	*<7**	*<7**	*<7**	*<7**
Chinese	20 (0.1)	15 (0.05)	*<7**	*<7**	*<7**	*<7**
Any other ethnic group	155 (0.4)	130 (0.36)	9 (0.3)	*<7**	*<7**	*<7**
Not stated	1237 (2.9)	3 467 (9.8)	301 (10.0)	134 (8.2)	299 (14.8)	88 (9.8)
Not known	3 869 (9.0)	654 (1.8)	58 (1.9)	26 (1.6)	51 (2.5)	28 (3.1)
Missing	0	0	0	0	0	0
	43 076	35 486	3028	1640	2027	895
**Co variates**						
Carpal Tunnel Syndrome	4 783	3 998 (11.2)	337 (10.9)	178 (10.9)	178 (8.8)	92 (10.3)
Knee Osteoarthritis	5 495	4 643 (13.0)	368 (11.9)	176 (10.7)	205 (10.1)	103 (11.5)
General Osteoarthritis	9 236	7 663 (21.4)	641 (20.8)	378 (23.0)	382 (18.8)	172 (19.2)
Rheumatoid Arthritis	109	81 (0.2)	7 (0.2)	7 (0.4)	12 (0.6)	2 (0.2)
Wrist Fracture	249	183 (0.5)	17 (0.6)	3 (0.2)	39 (1.9)	7 (0.8)
Oophorectomy	1 583	1 371 (3.8)	83 (2.7)	53 (3.2)	50 (2.5)	26 (2.9)

*Numbers less than 7 suppressed in line with NHS Digital disclosure control guidelines- percentages of other groups rounded to prevent secondary disclosure of data (29)

Supplementary Table 6 Local and systemic complications per surgery subtype

	Time	All surgery N (%[95%CI])	Trapeziectomy N (%, 95%CI)	LRTI N (%, 95%CI)	Arthroplasty N (%, 95%CI)	Arthrodesis N (%, 95%CI)	Partial Trapeziectomy N (%, 95%CI)
**Local complications**							
Wound dehiscence and wound infection	Within 30 days	12 (0.03%, [0.01-0.05])	10 (0.03% [0.01-0.05])	<7*	0	<7*	0
	Within 90 days	14 (0.03% [0.02-0.05])	12 (0.03%[0.02-0.06])	<7*	0	<7*	0
Neurovascular injury	Within 30 days	79 (0.18% [0.15-0.23])	68 (0.19%[0.15-0.24])	<7*	0	<7*	<7*
Any complication	Within 30 days	91 (0.21% [0.17-0.26])	78 (0.22%[0.17-0.27])	<7*	0	<7*	<7*
	Within 90 days	93 (0.22% [0.17-0.26])	80 (0.23%[0.18-0.28])	<7*	0	<7*	<7*
**Systemic complications**							
Stroke	Within 30 days	9 (0.02% [0.01-0.04])	9 (0.03% [0.01-0.05])	0	0	0	0
	Within 90 days	40 (0.09% [0.07-0.13])	34 (0.10%[0.07-0.13])	<7*	<7*	<7*	<7*
Respiratory Tract infection	Within 30 days	70 (0.16% [0.13-0.21])	67 (0.19%[0.15-0.24])	<7*	0	0	<7*
	Within 90 days	174 (0.40% [0.35-0.47])	144 (0.41%[0.34-0.48])	10 (0.33%[0.16-0.61])	8 (0.49%[0.21-0.96])	<7*	<7*
Myocardial infarction	Within 30 days	17 (0.04% [0.02-0.06])	15 (0.04% [0.02-0.07])	<7*	0	0	0
	Within 90 days	50 (0.11% [0.09-0.15])	40 (0.11% [0.08-0.15])	<7*	0	<7*	<7*
DVT/PE	Within 30 days	18 (0.04% [0.02-0.07])	9 (0.03% [0.01-0.05])	<7*	<7*	0	0
	Within 90 days	41 (0.10% [0.07-0.13])	30 (0.08%[0.06-0.12])	<7*	<7*	0	<7*
UTI	Within 30 days	30 (0.07% [0.05-0.10])	25 (0.07%[0.05-0.10])	<7*	0	<7*	0
	Within 90 days	75 (0.17%[0.14-0.22])	62 (0.17% [0.13-0.22])	9 (0.30%[0.14-0.56])	0	<7*	<7*
Acute Renal Failure	Within 30 days	13 (0.03%[0.02-0.05])	9 (0.03%[0.01-0.05])	<7*	0	<7*	0
	Within 90 days	44 (0.10%[0.07-0.14])	40 (0.11 [0.08-0.15])	<7*	0	<7*	0
Any Systemic Complication	Within 30 days	87 (0.20%[0.16-0.25])	67 (0.19% [0.15-0.24])	10 (0.33%[0.16-0.61])	<7*	<7*	0
	Within 90 days	250 (0.58% [0.51-0.66])	206 (0.58% [0.50-0.67])	24 (0.79%[0.51-0.12])	<7*	<7*	<7*
Any Systemic Complication (excl UTI & ARF)	Within 30 days	44 (0.10% [0.07-0.14])	33 (0.10% [0.06-0.13])	<7*	<7*	0	0
	Within 90 days	131 (0.30%[0.25-0.36])	104 (0.29% [0.24-0.36])	13 (0.43%[0.23-0.73])	<7*	<7*	<7*

DVT/PE deep vein thrombosis or pulmonary embolic disease
UTI urinary tract infection
ARF Acute Renal Failure
<7 Numbers less than 7 suppressed in line with NHS Digital disclosure control guidelines (29)

Supplementary Table 7 Risk of revision following any BTOA surgery Multivariable regression analysis accounting for competing risk of mortality (without surgical subtype as a risk in the model)

ALL SURGERIES	**Crude sHR[a]**	**95% CI**	**Adj sHR[a]**	**95% CI**
**Male**	1.22	0.99 to 1.49	1.24	1.01 to 1.52
**Age**				
40-49 years	1.53	1.11 to 2.12	1.53	1.10 to 2.13
50-59 years	1.12	0.91 to 1.38	1.14	0.92 to 1.40
60-69 years	1 (ref)	1 (ref)	1 (ref)	1 (ref)
70-79 years	0.86	0.66 to 1.10	0.85	0.66 to 1.10
>80 years	0.66	0.36 to 1.21	0.66	0.36 to 1.22
**Charlson comorbidity index**				
0	1 (ref)	1 (ref)	1 (ref)	1 (ref)
1	1.14	0.92 to 1.42	1.17	0.94 to 1.46
2	0.99	0.75 to 1.31	1.06	0.80 to 1.40
3+	0.92	0.73 to 1.17	1.01	0.78 to 1.30
**IMD**				
Least deprived 10%	1 (ref)	1 (ref)	1 (ref)	1 (ref)
Less deprived 10-20%	1.03	0.69 to 1.54	1.03	0.68 to 1.54
Less deprived 20-30%	1.16	0.78 to 1.72	1.14	0.77 to 1.70
Less deprived 30-40%	1.08	0.73 to 1.61	1.07	0.72 to 1.60
Less deprived 40-50%	1.24	0.84 to 1.83	1.21	0.82 to 1.79
More deprived 40-50%	1.04	0.66 to 1.62	0.99	0.63 to 1.55
More deprived 30-40%	1.24	0.82 to 1.86	1.18	0.79 to 1.78
More deprived 20-30%	1.66	1.14 to 2.42	1.61	1.10 to 2.34
More deprived 10-20%	1.51	1.04 to 2.20	1.47	1.01 to 2.15
Most deprived 10%	1.03	0.65 to 1.63	0.98	0.62 to 1.55
[a] Subhazard ratio				

Supplementary Table 8 Risk of revision following any BTOA surgery Multivariable regression analysis accounting for competing risk of mortality, including surgical subtype as a factor

ALL SURGERIES, including SURG SUBTYPE	**Crude sHR[a]**	**95% CI**	**Adj sHR[a]**	**95% CI**
**Male**	1.22	0.99 to 1.49	1.11	0.89 to 1.38
**Age**				
40-49 years	1.53	1.11 to 2.12	1.24	0.95 to 1.87
50-59 years	1.12	0.91 to 1.38	1.09	0.88 to 1.34
60-69 years	1 (ref)	1 (ref)	1 (ref)	1 (ref)
70-79 years	0.86	0.66 to 1.10	0.86	0.67 to 1.11
>80 years	0.66	0.36 to 1.21	0.67	0.37 to 1.24
**Charlson comorbidity index**				
0	1 (ref)	1 (ref)	1 (ref)	1 (ref)
1	1.14	0.92 to 1.42	1.17	0.94 to 1.46
2	0.99	0.75 to 1.31	1.05	0.79 to 1.40
3+	0.92	0.73 to 1.17	0.99	0.77 to 1.28
**IMD**				
Least deprived 10%	1 (ref)	1 (ref)	1 (ref)	1 (ref)
Less deprived 10-20%	1.03	0.69 to 1.54	1.00	0.67 to 1.50
Less deprived 20-30%	1.16	0.78 to 1.72	1.13	0.76 to 1.67
Less deprived 30-40%	1.08	0.73 to 1.61	1.06	0.71 to 1.58
Less deprived 40-50%	1.24	0.84 to 1.83	1.19	0.81 to 1.77
More deprived 40-50%	1.04	0.66 to 1.62	0.98	0.63 to 1.53
More deprived 30-40%	1.24	0.82 to 1.86	1.16	0.77 to 1.75
More deprived 20-30%	1.66	1.14 to 2.42	1.58	1.09 to 2.29
More deprived 10-20%	1.51	1.04 to 2.20	1.45	1.00 to 2.11
Most deprived 10%	1.03	0.65 to 1.63	0.96	0.61 to 1.52
**Surgery subtype**				
Simple trapeziectomy	1 (ref)	1 (ref)	1 (ref)	1 (ref)
Trapeziectomy + LRTI	1.22	0.86 to 1.73	1.16	0.82 to 1.66
Arthroplasty	2.51	1.81 to 3.48	2.49	1.80 to 3.44
Arthrodesis	2.55	1.91 to 3.40	2.40	1.78 to 3.26
Partial Trapeziectomy	1.44	0.81 to 2.55	1.46	0.82 to 2.59
[a] Subhazard ratio				

Supplementary Table 9 Risk of revision following Trapeziectomy Multivariable regression analysis accounting for competing risk of mortality

TRAPEZIECTOMY	**Crude sHR[a]**	**95% CI**	**Adj sHR[a]**	**95% CI**
**Male**	1.31	1.01 to 1.70	1.32	1.02 to 1.72
**Age**				
40-49 years	1.21	0.76 to 1.93	1.20	0.74 to 1.92
50-59 years	0.96	0.74 to 1.25	0.97	0.73 to 1.27
60-69 years	1 (ref)	1 (ref)	1 (ref)	1 (ref)
70-79 years	0.88	0.65 to 1.19	0.92	0.67 to 1.24
>80 years	0.53	0.23 to 1.19	0.55	0.24 to 1.25
**Charlson comorbidity index**				
0	1 (ref)	1 (ref)	1 (ref)	1 (ref)
1	0.96	0.73 to 1.26	0.97	0.73 to 1.28
2	0.80	0.56 to 1.15	0.79	0.54 to 1.15
3+	0.85	0.63 to 1.14	0.86	0.63 to 1.19
**IMD**				
Least deprived 10%	1 (ref)	1 (ref)	1 (ref)	1 (ref)
Less deprived 10-20	0.86	0.51 to 1.43	0.86	0.51 to 1.43
Less deprived 20-30%	1.03	0.63 to 1.68	1.02	0.63 to 1.67
Less deprived 30-40%	0.97	0.59 to 1.59	0.97	0.59 to 1.60
Less deprived 40-50%	1.13	0.70 to 1.82	1.12	0.69 to 1.81
More deprived 40-50%	0.89	0.50 to 1.57	0.89	0.50 to 1.57
More deprived 30-40%	1.01	0.60 to 1.70	1.00	0.59 to 1.69
More deprived 20-30%	1.68	1.07 to 2.64	1.68	1.07 to 2.63
More deprived 10-20%	1.59	1.01 to 2.49	1.59	1.01 to 2.49
Most deprived 10%	1.06	0.61 to 1.85	1.07	0.62 to 1.85
[a] Subhazard ratio				

Supplementary Table 10. Risk of revision following Trapeziectomy with LRTI
Multivariable regression analysis accounting for competing risk of mortality

LRTI	**Crude sHR**	**95% CI**	**Adjusted sHR[a]**	**95% CI**
**Male**	1.26	0.60 to 2.67	1.29	0.60 to 2.76
**Age**				
40-49 years	3.10	1.32 to 7.33	2.64	1.02 to 6.86
50-59 years	0.85	0.39 to 1.88	0.79	0.36 to 1.74
60-69 years	1 (ref)	1 (ref)	1 (ref)	1 (ref)
70-79 years	0.77	0.28 to 2.10	0.68	0.23 to 2.01
>80 years	0.77	0.10 to 5.83	0.81	0.10 to 6.28
**Charlson comorbidity index**				
0	1 (ref)	1 (ref)	1 (ref)	1 (ref)
1	1.14	0.55 to 2.34	1.04	0.50 to 2.13
2	0.79	0.29 to 2.09	0.85	0.31 to 2.31
3+	0.29	0.08 to 0.99	0.30	0.09 to 1.02
**IMD**				
Least deprived 10%	1 (ref)	1 (ref)	1 (ref)	1 (ref)
Less deprived 10-20%	0.40	0.04 to 4.39	0.39	0.04 to 4.26
Less deprived 20-30%	1.77	0.35 to 9.08	1.76	0.35 to 8.85
Less deprived 30-40%	1.39	0.25 to 7.64	1.34	0.24 to 7.33
Less deprived 40-50%	2.63	0.55 to 12.66	2.84	0.59 to 13.55
More deprived 40-50%	2.88	0.56 to 14.92	2.75	0.55 to 13.75
More deprived 30-40%	2.17	0.42 to 11.14	1.90	0.37 to 9.68
More deprived 20-30%	0.85	0.12 to 6.02	0.83	0.12 to 5.89
More deprived 10-20%	1.81	0.35 to 9.35	1.72	0.34 to 8.66
Most deprived 10%	1.52	0.22 to 10.73	1.22	0.17 to 8.92

[a] Subhazard ratio

Supplementary Table 11. Risk of revision following BTOA Arthroplasty Multivariable regression analysis accounting for competing risk of mortality

Arthroplasty	**Crude sHR**	**95% CI**	**Adj sHR[a]**	**95% CI**
**Male**	0.66	0.31 to 1.40	0.72	0.33 to 1.60
**Age**				
40-49 years	0.92	0.32 to 2.69	1.07	0.37 to 3.10
50-59 years	0.75	0.37 to 1.48	0.80	0.40 to 1.59
60-69 years	1 (ref)	1 (ref)	1 (ref)	1 (ref)
70-79 years	0.83	0.36 to 1.89	0.76	0.31 to 1.84
>80 years	1.63	0.37 to 7.13	1.41	0.28 to 7.25
**Charlson comorbidity index**				
0	1 (ref)	1 (ref)	1 (ref)	1 (ref)
1	1.84	0.83 to 4.11	1.84	0.79 to 4.30
2	2.73	1.16 to 6.42	2.63	1.08 to 6.40
3+	2.47	1.12 to 5.43	2.14	0.88 to 5.24
**IMD**				
Least deprived 10%	1 (ref)	1 (ref)	1 (ref)	1 (ref)
Less deprived 10-20	1.77	0.45 to 6.89	1.71	0.44 to 6.62
Less deprived 20-30%	1.61	0.38 to 6.78	1.44	0.34 to 6.18
Less deprived 30-40%	3.23	0.87 to 12.00	2.96	0.78 to 11.22
Less deprived 40-50%	1.98	0.48 to 8.24	1.47	0.33 to 6.53
More deprived 40-50%	3.02	0.76 to 12.02	2.72	0.68 to 10.91
More deprived 30-40%	1.58	0.36 to 7.04	1.35	0.30 to 6.02
More deprived 20-30%	1.37	0.31 to 6.06	1.31	0.28 to 6.18
More deprived 10-20%	0.65	0.11 to 3.88	0.60	0.10 to 3.59
Most deprived 10%	1.91	0.42 to 8.65	1.22	0.24 to 6.30

[a] Subhazard ratio

Supplementary Table 12. Risk of revision following BTOA Arthrodesis Multivariable regression analysis accounting for competing risk of mortality

Arthrodesis	**Crude sHR**	**95% CI**	**Adj sHR[a]**	**95% CI**
**Male**	0.71	0.42 to 1.21	0.72	0.42 to 1.25
**Age**				
40-49 years	0.74	0.33 to 1.64	0.70	0.31 to 1.54
50-59 years	1 (ref)	1 (ref)	1 (ref)	1 (ref)
60-69 years	0.71	0.38 to 1.31	0.71	0.38 to 1.34
70-79 years	0.72	0.31 to 1.67	0.81	0.32 to 2.05
>80 years	0.51	0.07 to 3.72	0.54	0.08 to 3.86
**Charlson comorbidity index**				
0	1 (ref)	1 (ref)	1 (ref)	1 (ref)
1	2.07	1.10 to 3.88	2.18	1.16 to 4.10
2	1.36	0.60 to 3.12	1.48	0.62 to 3.55
3+	1.04	0.47 to 2.15	1.05	0.46 to 2.41
**IMD**				
Least deprived 10%	1 (ref)	1 (ref)	1 (ref)	1 (ref)
Less deprived 10-20	5.18	0.64 to 42.24	5.42	0.66 to 44.82
Less deprived 20-30%	4.06	0.49 to 33.89	4.16	0.49 to 35.07
Less deprived 30-40%	2.07	0.21 to 20.01	2.03	0.21 to 19.37
Less deprived 40-50%	4.09	0.49 to 34.21	3.93	0.46 to 33.33
More deprived 40-50%	1.69	0.15 to 18.84	1.62	0.14 to 18.29
More deprived 30-40%	6.59	0.83 to 52.31	6.50	0.79 to 53.61
More deprived 20-30%	10.00	1.30 to 76.46	9.77	1.26 to 75.72
More deprived 10-20%	6.05	0.76 to 48.20	5.92	0.72 to 49.01
Most deprived 10%	1.00	0.06 to 16.29	0.94	0.06 to 15.41

[a] Subhazard ratio

Supplementary Figures 1-5 Kaplan Meier plots of risk of further procedure following

1 Simple Trapeziectomy

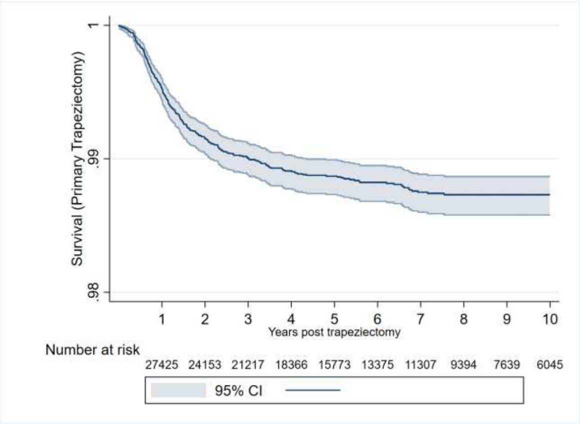

2 Trapeziectomy with LRTI

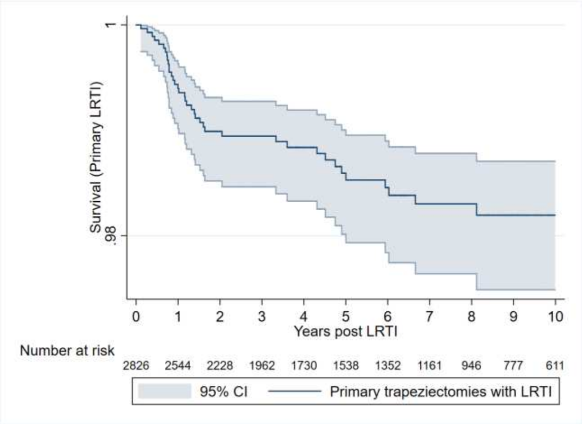

3 BTOA Arthroplasty

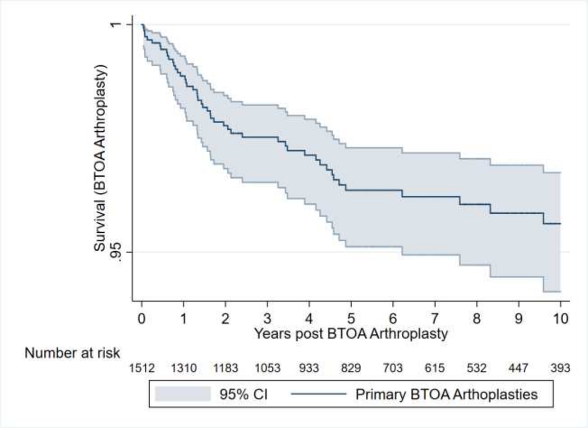

4 BTOA Arthrodesis

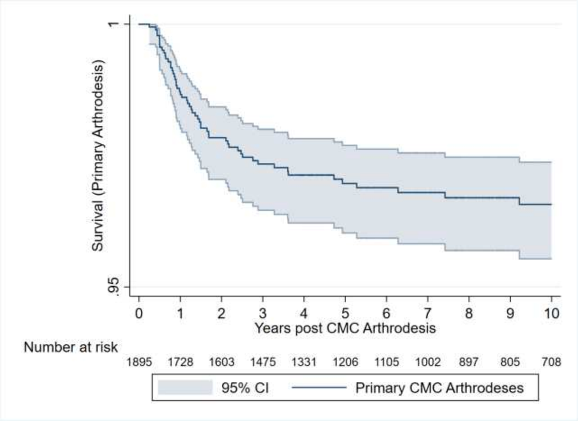

5 Partial Trapeziectomy

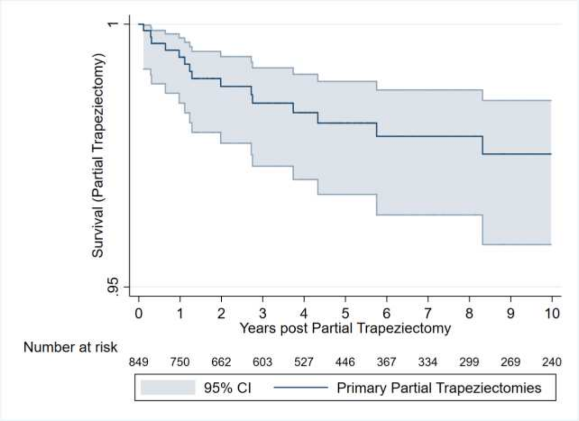

Supplement: Supplementary data [file bmjopen-2020-045614supp001.pdf]
