# Supplementary material for: Low rates of serious complications and further procedures following surgery for base of thumb osteoarthritis: analysis of a national cohort of 43 076 surgeries
Source: BMJ Open. 2021 Jul 7;11(7):e045614. doi: 10.1136/bmjopen-2020-045614 (PMC8264901; doi:10.1136/bmjopen-2020-045614)
Supplement: Author's manuscript [file bmjopen-2020-045614.draft_revisions.pdf]

BMJ Open is committed to open peer review. As part of this commitment we make the peer review history of every article we publish publicly available.

When an article is published we post the peer reviewers' comments and the authors' responses online. We also post the versions of the paper that were used during peer review. These are the versions that the peer review comments apply to.

The versions of the paper that follow are the versions that were submitted during the peer review process. They are not the versions of record or the final published versions. They should not be cited or distributed as the published version of this manuscript.

BMJ Open is an open access journal and the full, final, typeset and author-corrected version of record of the manuscript is available on our site with no access controls, subscription charges or pay-per-view fees (http://bmjopen.bmj.com).

If you have any questions on BMJ Open's open peer review process please email info.bmjopen@bmj.com

BMJ Open

Low rates of complications and further procedures following surgery for Base of Thumb Osteoarthritis: analysis of national cohort of 43 076 surgeries with up to 19 years of follow up

Journal:	*BMJ Open*
Manuscript ID	bmjopen-2020-045614
Article Type:	Original research
Date Submitted by the Author:	06-Oct-2020
Complete List of Authors:	Lane, Jennifer; University of Oxford Nuffield Department of Orthopaedics Rheumatology and Musculoskeletal Sciences<br>Craig, Richard; University of Oxford, Nuffield Department of Orthopaedics, Rheumatology and Musculoskeletal Sciences<br>Rees, J L; University of Oxford, Nuffield Department of Orthopaedics, Rheumatology and Musculoskeletal Sciences<br>Gardiner, Matthew; University of Oxford Nuffield Department of Orthopaedics Rheumatology and Musculoskeletal Sciences; Wexham Park Hospital, Plastic Surgery<br>Mikhail, Mark; Oxford University Hospitals NHS Foundation Trust Nuffield Orthopaedic Centre, Plastic Surgery<br>Riley, Nicholas; Oxford University Hospitals NHS Foundation Trust Nuffield Orthopaedic Centre, Hand Surgery<br>Prieto-Alhambra, Daniel; university of oxford, ndorms<br>Furniss, Dominic ; University of Oxford, Nuffield Department of Orthopaedics Rheumatology and Musculoskeletal Science; Oxford University Hospitals NHS Foundation Trust Nuffield Orthopaedic Centre, Plastic Surgery
Keywords:	SURGERY, Epidemiology < TROPICAL MEDICINE, Hand & wrist < ORTHOPAEDIC & TRAUMA SURGERY, Adult orthopaedics < ORTHOPAEDIC & TRAUMA SURGERY, RHEUMATOLOGY

SCHOLARONE™
Manuscripts

I, the Submitting Author has the right to grant and does grant on behalf of all authors of the Work (as defined in the below author licence), an exclusive licence and/or a non-exclusive licence for contributions from authors who are: i) UK Crown employees; ii) where BMJ has agreed a CC-BY licence shall apply, and/or iii) in accordance with the terms applicable for US Federal Government officers or employees acting as part of their official duties; on a worldwide, perpetual, irrevocable, royalty-free basis to BMJ Publishing Group Ltd ("BMJ") its licensees and where the relevant Journal is co-owned by BMJ to the co-owners of the Journal, to publish the Work in this journal and any other BMJ products and to exploit all rights, as set out in our licence.

The Submitting Author accepts and understands that any supply made under these terms is made by BMJ to the Submitting Author unless you are acting as an employee on behalf of your employer or a postgraduate student of an affiliated institution which is paying any applicable article publishing charge ("APC") for Open Access articles. Where the Submitting Author wishes to make the Work available on an Open Access basis (and intends to pay the relevant APC), the terms of reuse of such Open Access shall be governed by a Creative Commons licence – details of these licences and which Creative Commons licence will apply to this Work are set out in our licence referred to above.

Other than as permitted in any relevant BMJ Author's Self Archiving Policies, I confirm this Work has not been accepted for publication elsewhere, is not being considered for publication elsewhere and does not duplicate material already published. I confirm all authors consent to publication of this Work and authorise the granting of this licence.

For peer review only

Low rates of complications and further procedures following surgery for Base of Thumb Osteoarthritis: analysis of national cohort of 43 076 surgeries with up to 19 years of follow up

Jennifer C.E. Lane MRCS[1], Richard S. Craig DPhil FRCS (Tr&Orth)[1], Jonathan. L. Rees MD, FRCS (Tr&Orth) [1], Matthew D. Gardiner PhD, FRCS(Plast)[2,3], Mark Mikhail MRCS[4], Nicholas D Riley FRCS(Tr&Orth)[5], Daniel Prieto-Alhambra, DPhil[1*], Dominic Furniss, DM FRCS(Plast)[1,4*]

1. Oxford NIHR Musculoskeletal Biomedical Research Unit, Nuffield Department of Orthopaedics, Rheumatology and Musculoskeletal Sciences, Nuffield Orthopaedic Centre, University of Oxford, Windmill Road, Oxford, OX3 7LD, UK
2. Department of Plastic Surgery, Wexham Park Hospital, Slough, SL2 4HL, UK
3. Kennedy Institute of Rheumatology, Nuffield Department of Orthopaedics, Rheumatology and Musculoskeletal Sciences, Roosevelt Drive, Oxford, OX3 7FY, UK
4. Department of Plastic and Reconstructive Surgery, Nuffield Orthopaedic Centre, Windmill Road, Oxford, OX3 7HE, UK
5. Department of Hand Surgery, Nuffield Orthopaedic Centre, Windmill Road, Oxford, OX3 7HE, UK

* authors co-directed this work

Corresponding author: Professor Dominic Furniss, Nuffield Department of Orthopaedics, Rheumatology and Musculoskeletal Sciences, University of Oxford, Windmill Road, Oxford, OX3 7LD, UK dominic.furniss@ndorms.ox.ac.uk

Word Count (excl title page, abstract refs figures and tables): 2948

Keywords: base of thumb osteoarthritis, surgery, adverse event, revision, epidemiology

For peer review only

ABSTRACT

Objectives

To determine the incidence of further procedures and serious adverse events (SAE) following elective surgery for base of thumb osteoarthritis, and the patient factors associated with these outcomes.

Design

Population based cohort study

Setting

National Health Service (NHS) using the national Hospital Episode Statistics dataset linked to mortality records over a 19-year period from 01/04/1998-31/03/2017.

Participants

43 076 primary surgeries were followed longitudinally in secondary care until death or migration on 37 329 patients over the age of 18 years.

Main Outcome measures

Incidence of further procedures (including revision surgery or intra-articular steroid injection) at any post-operative time point, and post-operative local wound and systemic complications (myocardial infarction, stroke, respiratory tract infection, venous thromboembolic events, urinary tract infection or renal failure) within 30 and 90 days. Fine and Gray model regression analysis was used to identify factors influencing adverse outcome, adjusting for the competing risk of mortality in addition to age, overall comorbidity and socioeconomic status.

Results

Over the 19-year period, there was an increasing trend in surgeries undertaken. The overall rate of further procedures after any surgery was 1.39%; the lowest rates after simple trapeziectomy (1.12%), the highest rates seen after arthroplasty (3.84%), and arthrodesis (3.5%). When matched for age, comorbidity and socioeconomic status, those undergoing arthroplasty and arthrodesis were 2.5 times more likely to undergo a further procedure (subhazard ratio 2.51 [95%CI 1.81-3.48]) and 2.55 [1.91-3.40]) than those undergoing simple trapeziectomy. Overall complication rates following surgery were 0.21% within 30 days, 0.22% within 90 days for local complications and 0.20% within 30 days, 0.58% within 90 days for systemic complications.

Conclusions

The number of patients proceeding to BTOA surgery has increased over the last 19 years, with a low rate of further procedures and SAEs after surgery overall. Arthrodesis and arthroplasty had a significantly higher revision rate.

Trial Registration

Clinicaltrials.gov (NCT03573765)
NHS Digital Data Sharing Agreement (DARS-NIC-29827-Q8Z7Q)

For peer review only - http://bmjopen.bmj.com/site/about/guidelines.xhtml

STRENGTHS AND LIMITATIONS OF THIS STUDY

- To our knowledge, this is the largest scale national study identifying the risks following surgery for base of thumb osteoarthritis
- Long follow up is enabled by a nationalised health system linked to mortality records, showing that has been an increasing rate of surgical intervention in England for base of thumb osteoarthritis over the last 19 years
- The risk of further procedure was 2.5 times higher for patients undergoing arthroplasty and arthrodesis, but the overall risk of revision surgery at any time post operatively (1.39%) and serious post-operative adverse events at 90 days (0.58%) remained low
- This study uses data collected from routine clinical care producing results that are generalisable to the whole population, but only identifies complications presenting in secondary care

For peer review only

INTRODUCTION

Background

Osteoarthritis of the first carpometacarpal joint, or base of thumb osteoarthritis (BTOA), is very common and may need surgical treatment.[1] Worldwide, the majority of surgical interventions are simple trapeziectomy with or without ligament reconstruction and tendon interposition (LRTI).[1-3] However, there is increasing interest in the use of arthroplasty for BTOA, with many different implants available.[4-10] Concerns have been raised regarding increased risk of complications with LRTI and arthroplasty compared with simple trapeziectomy.[11, 12] A Cochrane review comparing surgical techniques concluded that there was a high risk of bias within current studies, with no single technique presenting superior results.[13] Recent work aimed to identify complications within a large insurance provider in the US for small joint hand osteoarthritis procedures, and found complication rates as high as 35%, including 5% risk of a complication identified related to the prosthesis within two years of surgery.[14]

Many surgical studies in BTOA compare two techniques either within a clinical trial or single centre cohort, but there are very few studies comparing all available techniques with a long follow up at a national level. Routinely collected data produced from everyday clinical practice offers the advantage of enabling long-term follow up and detection of rare outcomes for healthcare interventions in the general population. [15]

The primary aim of this study was to estimate the incidence of BTOA surgery, further procedures and complications in adults in the NHS in England. Secondary aims were to identify factors associated with adverse outcomes, including local and systemic complications, and further procedures after surgery. Our hypothesis was that there is variation in outcome between surgical subtypes.

METHODS

Data Source

A pseudonymised, bespoke extract was made from the NHS database Hospital Episode Statistics Admitted Patient Care (HES APC) covering the period 1st April 1998 to 31st March 2017).[16] This individual patient level data extract contained all patient episodes of care within the NHS England system, including those episodes that occur within independent providers that are remunerated by the NHS. The NHS provides the majority of healthcare in England, with only 13% of elective surgery estimated to be privately funded.[17] Episodes of care are linked via a patient's individual NHS number, therefore enabling linkage of all treatments undertaken and longitudinal follow up, including complications and revision surgeries undertaken by any provider. The HES APC extract was also linked to the ONS national mortality dataset prior to pseudonymization to identify cause and date of death.[18] By design, raw data was collected by NHS Digital prior to delivery to the research team.

Population

All patients over 18 years undergoing BTOA surgery were identified using a list of OPCS Classification of Interventions and Procedures (OPCS-4.8) and International Classification of Diseases (ICD-10) codes (Supplementary tables 1-3). All episodes of care for each individual prior to and following BTOA surgery were included in the extract. Patients were followed up until date of death or the end of the study period (31/03/2017). Patients who had an ICD-10 code for traumatic injury in the same episode as BTOA surgery were excluded, in order to only include patients undergoing elective surgery for longstanding BTOA. Duplicate

For peer review only - http://bmjopen.bmj.com/site/about/guidelines.xhtml

episodes can occur over the change of financial year, and were removed during data cleaning. Each hand was considered as a separate surgical case.
Validation studies in order to appropriately identify BTOA surgery cases took place in three phases. Firstly, we undertook consensus discussion between surgeons, coders and NHS Digital to determine the most likely OPCS procedure codes and ICD diagnosis codes. Secondly, we undertook a validation study to identify BTOA as a condition [19-21]. Finally, we undertook further validation of OPCS procedure codes in order to identify surgery subtype in our NHS trust. In a year's sample of 104 patients undergoing BTOA surgery, our code list found a positive predictive value of 99% in identifying surgical subtype.

Covariates
Socioeconomic status of patients was identified using the Index of Multiple Deprivation (IMD), a government generated score of deprivation upon geographical location in England.[22] Overall comorbidity level was calculated using the Charlson Comorbidity Index (CCI). [23, 24] Baseline incidence of other conditions associated with BTOA pathogenesis in the literature were also identified using OPCS and ICD codes found previous episodes of care, including a past medical history of carpal tunnel syndrome, knee osteoarthritis, generalized osteoarthritis, rheumatoid arthritis, wrist fracture, and oophorectomy.

Further procedures
Further procedure was defined as any revision surgery or intra-articular steroid injection in the same thumb base following surgery. In order to identify factors associated with further procedure, time to further procedure and produce an incidence rate, only further procedures identified using two episodes with BTOA surgery or injection codes in the same hand were used, identified through laterality code. Also, to produce an estimation of a 'worst case' scenario of the percentage of cases that may have proceeded to further procedure, patients who had 3 or more episodes with BTOA surgery or injection codes but missing laterality were included in the percentage, but were not included in all subsequent analyses.

Complications
Complications were grouped into local (surgical site infection or dehiscence requiring surgery, and neurovascular or tendon injury) and systemic (stroke, respiratory tract infection, acute myocardial infarction, venous thromboembolic disease, urinary tract infection and acute renal failure). Complications were identified within 30 and 90 days of surgery to align with NHS outcomes framework used to compare outcomes between different medical conditions.[25]

Statistical methods
Age and sex specific incidence of surgery were calculated using national mid-year population estimates.[26] All complications were calculated as a proportion of the sample with 95% confidence intervals. Incomplete records were found for 0.95% of cases for age, sex, ethnicity and IMD deciles. Data was assumed to be missing at random, and a complete case analysis undertaken without imputation. Laterality was missing in 16.2% of cases, and in order to assess if missing laterality caused a bias in included cases, the baseline demographics of patients with and without laterality were compared (supplementary table 5). No difference in baseline demographics was seen and therefore this was not considered as a potential cause of bias.

Factors associated with further procedure were identified using a multivariable regression analysis using a Fine and Gray model that accounts for the competing risk of mortality.[27] Adjustment was undertaken for age, socioeconomic status (using IMD) and overall comorbidity (using CCI). As complications were assessed within 90 days of surgery, multivariable cox proportional hazard modelling was used without adjusting for the competing risk of mortality. Patient age was identified as having a non-linear relationship with further procedure, and therefore was categorized, with the category containing the

For peer review only - http://bmjopen.bmj.com/site/about/guidelines.xhtml

median age used as the reference category. When dividing the risk of further procedure by surgical subtype, there were insufficient patients below the age of 40 for inclusion in the multivariable regression analysis. Multivariable regression analysis was undertaken for all patient requiring further procedure, and also for those who had trapeziectomy alone, trapeziectomy with LRTI, arthroplasty and arthrodesis. For partial trapeziectomy there were insufficient numbers of patients who had a further procedure to undertake this analysis.

Statistical analysis was undertaken using STATA version 15.1. Significance within the multivariable regression analysis was determined for a subhazard ratio (sHR) when a 95% confidence interval did not include 1, as p values in multiple regression analysis will find spurious 'significant' results due to multiple testing. No power calculation was undertaken as this study aimed to use all available data within a national cohort analysis.

PATIENT AND PUBLIC INVOLVEMENT

A James Lind Alliance priority setting partnership focused on common conditions affecting the hand and wrist also raised the question *'which interventions give the best results in the treatment of painful joints in the hand and wrist'* as important to patients, making this study a direct response in its research question.[28] No patients were directly involved in the study design, conduct or interpretation of the study.

RESULTS

Data processing

Figure 1 describes the data processing details. In the study period, 43 076 primary BTOA surgeries were performed on 37 329 individuals in English NHS hospitals. 79.1% were performed on female patients, and the mean age at primary surgery was 63 (SD 9.2).

Demographics of patients undergoing BTOA surgery are shown in Table 1, with further granularity given in Supplementary Table 4. Over 60% of patients had no or very low levels of overall comorbidity, and 86% of patients were of white ethnic background. Patients were more likely to come from affluent sections of society. Approximately 1 in 5 patients had another hospital episode prior to BTOA surgery for osteoarthritis elsewhere in the body.

Table 1. Demographics of patients undergoing BTOA surgery

	**All surgery (N, %) (total=43 076)**	**Trapeziectomy N (%) (35 486)**	**LRTI N (%) (3028)**	**Arthroplasty N (%) (1640)**	**Arthrodesis N (%) (2027)**	**Partial Trapeziectomy N (%) (894)**
**Female sex**	34 112 (79.1)	28 655 (80.8)	2411 (79.6)	1250 (76.2)	1092 (53.9)	704 (78.7)
**Mean age (SD; years)**	63.2 (SD 9.2)	63.6 (SD 8.8)	62.5 (SD 9.7)	61.7 (SD 9.3)	58.1 (11.7)	63.1 (9.8)
**Charlson Comorbidity index**						
0	18 279 (42.4)	14 992 (42.3)	1 324 (43.8)	677 (41.3)	890 (43.9)	396 (44.3)
1	10 640 (24.7)	8 807 (24.8)	721 (23.8)	444 (27.1)	475 (23.4)	193 (21.6)
2	5 693 (13.2)	4 746 (13.4)	392 (13.0)	199 (12.1)	250 (12.3)	106 (11.8)
3	3 286 (7.6)	2 723 (7.7)	222 (7.3)	125 (7.6)	142 (7.0)	74 (8.3)
4	1 659 (3.9)	1 359 (3.8)	123 (4.1)	60 (3.7)	77 (3.8)	40 (4.5)
>=5	3 519 (8.2)	2 859 (8.0)	246 (8.1)	135 (8.2)	193 (9.6)	86 (8.5)
**Index of Multiple Deprivation**						
Quintile 1 (least deprived)	9 518 (22.1)	7 949 (22.4)	582 (19.2)	435 (26.5)	348 (17.2)	204 (22.8)
Quintile 2	9 637 (22.4)	7 950 (22.4)	747 (24.6)	342 (20.9)	424 (21)	174 (19.5)
Quintile 3	7 961 (18.5)	6553 (18.5)	563 (18.6)	264 (16.1)	402 (19.8)	179 (20)
Quintile 4	8 004 (18.6)	6550 (18.5)	576 (19.1)	309 (18.8)	417 (20.6)	152 (17)
Quintile 5 (Most deprived)	7 598 (17.7)	6229 (17.5)	514 (17.0)	282 (17.2)	393 (19.4)	180 (20.1)

For peer review only

Missing	358 (0.8)	255 (0.7)	46 (1.5)	8 (0.5)	43 (2.0)	6 (0.7)

Trends in surgery
Over the 19-year period there was an increasing trend in the number of BTOA surgeries being undertaken, predominantly accounted for by a steady increase in simple trapeziectomy.

Further Procedures

The overall rate of further procedure after any BTOA surgery was 1.39%, with the median time to intervention being under 1.5 years (Table 2; Figure 3). Whilst the rate of further procedures was low in general, the highest rates were seen after arthroplasty (3.84%) and arthrodesis (3.50%) and the lowest following simple trapeziectomy (1.12%). The survival of BTOA surgery over time according to surgical subtype is given in Kaplan-Meier plots in the appendix (Supplementary Figures 1-5), noting the majority of patients proceeded to further intervention around the first 18 months post-operatively

Table 2. Rate of further procedure (revision surgery or intraarticular steroid injection) according to BTOA surgical subtype

	Number of cases	Median follow up time in days (IQR)	Number cases needing further procedures (%)	Median time to further procedure in days (IQR)	Further procedure rate per 1000 person years (95%CI)
All BTOA surgeries	43 076	1835 (816 to 3223)	599 (1.39%)	472 (272 to 965)	1.72 (1.54 to 1.92)
Trapeziectomy	35 486	1839 (820 to 3228)	398 (1.12%)	420.5 (248 to 804)	1.73 (1.55 to 1.93)
Trapeziectomy with LRTI	3 028	2063.5 (880 to 3405)	45 (1.49%)	508 (287 to 1648)	2.13 (1.56 to 2.92)
Arthroplasty	1 640	2000.5 (865 to 3683.5)	63 (3.84%)	732.5 (325 to 1666.5)	4.83 (3.65 to 6.40)
Arthrodesis	2 027	2712.5 (1222 to 4546)	71 (3.50%)	520 (301 to 1054)	3.91 (3.04 to 5.04)
Partial Trapeziectomy	894	1972 (929 to 4070)	22 (2.46%)	673.5 (296.5 to 1599.5)	2.63 (1.59 to 4.37)

Complications

Overall there was a low rate of serious local and systemic complications following surgery, with the risk of any serious local complication being 0.22% within 90 days, and 0.58% for systemic complications within 90 days (Table 3; supplementary table 6). As there were a low number of complications seen, there was insufficient number of cases to enable further analyse factors associated with complications.

Table 3. Local and systemic complications following all BTOA surgery

	Time	All surgery N (%[95%CI])
**Local complications**		
Wound dehiscence and wound infection	Within 30 days	12 (0.03% [0.01-0.05])
	Within 90 days	14 (0.03% [0.02-0.05])
Neurovascular injury	Within 30 days	79 (0.18% [0.15-0.23])
Any complication	Within 30 days	91 (0.21% [0.17-0.26]
	Within 90 days	93 (0.22% [0.17-0.26]
**Systemic complications**		
Stroke	Within 30 days	9 (0.02% [0.01-0.04])

For peer review only - http://bmjopen.bmj.com/site/about/guidelines.xhtml

	Within 90 days	40 (0.09% [0.07-0.13])
Respiratory Tract infection	Within 30 days	70 (0.16% [0.13-0.21])
	Within 90 days	174 (0.40% [0.35-0.47])
Myocardial infarction	Within 30 days	17 (0.04% [0.02-0.06])
	Within 90 days	50 (0.11% [0.09-0.15])
DVT/PE	Within 30 days	18 (0.04% [0.02-0.07])
	Within 90 days	41(0.10% [0.07-0.13])
UTI	Within 30 days	30 (0.07% [0.05-0.10]
	Within 90 days	75 (0.17% [0.14-0.22])
Acute Renal Failure	Within 30 days	13 (0.03% [0.02-0.05])
	Within 90 days	44 (0.10% [0.07-0.14])
Any Systemic Complication	Within 30 days	87 (0.20% [0.16-0.25])
	Within 90 days	250 (0.58% [0.51-0.66])
Any Systemic Complication (excl UTI & ARF)	Within 30 days	44 (0.10% [0.07-0.14])
	Within 90 days	131 (0.30% [0.25-0.36])

Factors associated with further procedure

When considering the impact of age, sex, and sociodemographic factors, the risk of further procedure was increased for those in a younger age category of 40-49 years (adjusted subhazard ratio 1.53 [95%CI 1.10 to 2.13]) and in men (adjusted sHR 1.24 [1.01 to 1.53]) (Supplementary table 7) when adjusting for the other factors. However, when the additional factor of surgical subtype was included, the increased risk in men and those who underwent surgery at a younger age did not remain significant. This suggests that increased risk of further procedure was likely to be due to a greater proportion of younger male patients undergoing arthroplasty and arthrodesis (Supplementary table 8). When compared to those undergoing simple trapeziectomy, those undergoing arthroplasty and arthrodesis were around 2.5 times more likely to proceed to further procedure (arthroplasty 2.46 [1.77 to 3.42]; arthrodesis 2.41 [1.78 to 3.28]) after adjustment for age, sex and deprivation. The forest plot in Figure 4 shows the impact of age, sex, socioeconomic deprivation and surgical subtype seen upon the risk of undergoing a further procedure.

When considering the impact of age, sex and deprivation on the risk of further procedure within each surgical subtype, an increased risk of further procedure was seen in men for those who underwent trapeziectomy alone when adjusted for age and deprivation (adjusted sHR 1.33 [1.02 to 1.73]). This was not seen in those undergoing LRTI, arthrodesis or arthroplasty, but noting there were fewer cases in these subgroups. Age and sociodemographic status did not appear significant in any of the surgical subgroups in regression analysis. (Supplementary tables 9-12).

DISCUSSION

Principle Findings

Patients undergoing surgery for BTOA in England are predominantly females and in their 7th decade. There has been an increase in surgery undertaken over the last 19 years. Surgery for BTOA is safe. There is a low rate of revision surgery and intraarticular steroid injection following surgery. In addition, there are low rates of local and systemic complications. Further procedures occur mostly within the first 18 months post operatively. The greatest factor influencing progression to further procedure is the surgical subtype, with patients receiving an arthroplasty around 2.5 times more likely to undergo further procedure than those having simple trapeziectomy.

Strengths and contribution to current knowledge

The major strength of this study is the national nature of the study setting. This large national cohort of patients provides longitudinal follow up of individuals across multiple providers. It allows retention in the study of those individuals who may have sought a second opinion at a

For peer review only - http://bmjopen.bmj.com/site/about/guidelines.xhtml

different hospital in England. This gives a better overview of the need for revision surgery and for the management of serious adverse events following surgery than is possible in single centre or regional studies. It also accounts for patient movement within the country, noting that emigration out of the UK is thought to be low at around 400,000 persons per year.[29]

This study includes all adult patients who underwent surgery in England, and is representative of the generality of hand surgery practice in a large public healthcare system. Patients at extremes of ages, with comorbid conditions that may render them ineligible to take part in clinical trials, are included. Due to practical and financial constraints, randomised control trials typically lack the statistical power and longevity of follow up to reliably inform on the safety of surgical procedures for BTOA. Large observational studies, such as this one, are important to fill that evidence gap. Previous studies have suggested increased revision risk in those of a younger age which we also detected here in a univariable analysis. The effect did not persist here in multivariable analysis, and may therefore be more directly related to choice of surgery type.[30, 31]

All widely used surgical procedure subtypes are included in this study, enabling a full comparison of each main procedure with the other. This facilitates broad comparison of the rate of complications and revision across an inclusive population with longer follow up than most clinical trials.[12, 13] This study emphasises that whilst the overall risk of BTOA surgery is low, there is an increased risk of further procedure for those undergoing arthroplasty that warrants further large scale epidemiological analysis, as has been seen in joint arthroplasty research at other anatomical sites.[32, 33]

Limitations and future work

This study focusses on the patient factors that can impact the risk of adverse outcome. It does not account for potential variation associated with surgeon or hospital factors. Further methodological research is underway to identify the best method of multilevel analysis that can explore the interaction of healthcare provider factors with patient factors. This technique may enable us to identify how multiple factors influence the risk of adverse events.

Patients included here are those who have been identified by NHS Digital records, a dataset that is designed for remuneration rather than research. Care was taken to validate the codes in two different populations used to identify patient records in order to limit misclassification bias, but this remains a potential limitation of the work. All arthroplasties are coded as a generic arthroplasty code, and therefore implant type cannot be analysed. Future work focussing on implant registries, such as currently used in hip and knee arthroplasty, is needed to determine the impact of implant type upon outcome in routine clinical practice.[34, 35]

Comorbidities registered in this dataset are necessarily identified within an admitted patient episode, which may lead to inclusion bias of information used for remuneration. Whilst admitted patient care gives the best granularity of information for surgery and in-hospital events, it does not cover interactions occurring in an outpatient or primary care environment. Therefore, this study only identifies serious adverse events; those requiring at least a day case admission to hospital. It will fail to identify some pre-existing comorbidities in our patients, or persistent pain. Complications treated in the community such as minor surgical site infections requiring oral antibiotics will also not be identified using this data. Our future work is focussed upon identifying patients who have undergone BTOA surgery in primary care records, to compare the information collected here regarding comorbidity and post-operative infection.

Finally, this study uses revision surgery and serious adverse events as a proxy for outcome following surgery, as no patient reported outcomes (PROMS) are routinely collected for hand surgery at a national level by the NHS. Further work is needed to focus upon comparing PROMS for each surgical subtype in routine clinical practice to identify the quality of outcome from the patient perspective.

Meaning and use for clinical practice

For peer review only - http://bmjopen.bmj.com/site/about/guidelines.xhtml

As this study includes a national cohort of patients from routine clinical practice, the rates of further procedure and serious adverse events given in this study are directly of use for counselling patients during the process of consent and shared decision making. The higher rates of further procedure following arthroplasty found in this study concurs with a previous systematic review of smaller trials.[12] Within a large cohort of patients, demographic factors did not appear to be associated with significantly increased risk of adverse outcomes when accounting for surgical subtype, which again is informative for both the surgeon and patient. The study suggests that there is a low overall rate of further procedures and complications following surgery for BTOA. Whilst the risk of further procedure does vary by surgical subtype, demographic factors do not appear to influence this risk.

FIGURE LEGENDS

Figure 1 Data processing flow chart
Figure 2 Age specific incidence of BTOA surgeries by year, England 1998-2017
Figure 3 Kaplan Meier plot of risk of further procedure after all BTOA surgery, divided by sex
Figure 4 Forest plot of relative risk of further procedure after primary BTOA surgery within multivariable regression analysis adjusted for age, sex, sociodemographic status and surgical subtype

ETHICAL APPROVAL

This study was registered at clinicaltrials.gov (NCT03573765) and approved by the University Research Services Clinical Trials Research Group (project ID 12787), and the NHS Data Access Advisory Group (DAAG). It was carried out in accordance with the NHS Digital Data Sharing Agreement (DARS-NIC-29827-Q8Z7Q). Studies using non-identifiable records from Hospital Episode Statistics are exempt from research ethics committee approval. Patients have the right to request that their data is not released by NHS Digital for use by researchers (register a “Type 2 opt-out”).

CONTRIBUTOR STATEMENT

JCEL, DF, RC, JLR, MG and DPA conceived the study and designed the study method. MM and NDR undertook the validation study. JCEL and RC acquired the data. JCEL performed the data analysis. JCEL, DF, RC, JLR, MG, NDR and DPA interpreted the data. JCEL drafted the manuscript. All authors reviewed and edited the final manuscript. JCEL, RC, JR and DF has full access to all data in the study. DF is the guarantor.

ACKNOWLEDGEMENTS

The authors would like to acknowledge Miss Rachel Kuo and Professor Jane Green for their work in this study.

ROLE OF THE FUNDING SOURCE

This work was supported by Versus Arthritis [21605] (JL); the Medical Research Council [MR/K501256/1] (JL); RCS England/ NJR research fellowship (RC); University of Oxford (JR); the National Institute for Health Research (NIHR) [SRF-2018-11- ST2-004] (DPA); NIHR Biomedical Research Centre, Oxford (BRC) (RC, DF, JR) and Oxford Medical Research Fund (MDG). No funders had a direct role in this study. The views and opinions expressed are those of the authors and do not necessarily reflect those of the Clinician Scientist Award programme, NIHR, NHS or the Department of Health. This work used data supplied by NHS Digital copyright 2018, re-used with the permission of NHS Digital. All rights reserved.

COMPETING INTERESTS

All authors have completed an ICJME conflict of interest form that is uploaded with the study (http://www.icmje.org/conflicts-ofinterest/) no support from any organization for the submitted work; D P-A has received research grants from Amgen, Servier, UCB; departmental fees for

For peer review only - http://bmjopen.bmj.com/site/about/guidelines.xhtml

speaker services from Amgen, departmental fees for consultancy from UCB.JL reports grants from the Medical Research Council (MR/K501256/1) and Versus Arthritis (21605), outside of the submitted work. NR reports personal fees from Acumed, outside the submitted work; Dr. Craig reports grants from Royal College of Surgeons/National Joint Registry, outside the submitted work. No other relationships or activities could appear to have influenced the submitted work.

TRANSPARENCY STATEMENT

The lead author affirms that the manuscript is an honest, accurate, and transparent account of the study being reported; no important aspects of the study have been omitted, and that any discrepancies from the study as originally planned have been explained.

DATA SHARING STATEMENT

No further data can be made available due to NHS Digital restrictions. Data extracts can be applied for via the NHS Digital data access request service (https://digital.nhs.uk/services/data-access-request-service-dars).

EXECUTIVE LICENSE

The Corresponding Author has the right to grant on behalf of all authors and does grant on behalf of all authors, a worldwide licence (http://www.bmj.com/sites/default/files/BMJ%20Author%20Licence%20March%202013.doc) to the Publishers and its licensees in perpetuity, in all forms, formats and media (whether known now or created in the future), to i) publish, reproduce, distribute, display and store the Contribution, ii) translate the Contribution into other languages, create adaptations, reprints, include within collections and create summaries, extracts and/or, abstracts of the Contribution and convert or allow conversion into any format including without limitation audio, iii) create any other derivative work(s) based in whole or part on the on the Contribution, iv) to exploit all subsidiary rights to exploit all subsidiary rights that currently exist or as may exist in the future in the Contribution, v) the inclusion of electronic links from the Contribution to third party material where-ever it may be located; and, vi) licence any third party to do any or all of the above. All research articles will be made available on an open access basis (with authors being asked to pay an open access fee—see http://www.bmj.com/about-bmj/resources-authors/forms-policies-and-checklists/copyright-open-access-and-permission-reuse). The terms of such open access shall be governed by a Creative Commons licence—details as to which Creative Commons licence will apply to the research article are set out in our worldwide licence referred to above.

REFERENCES

1. Yuan F, Aliu O, Chung KC, Mahmoudi E. Evidence-Based Practice in the Surgical Treatment of Thumb Carpometacarpal Joint Arthritis. J Hand Surg Am. 2017;42(2):104-12 e1.
2. Field J, Buchanan D. To suspend or not to suspend: a randomised single blind trial of simple trapeziectomy versus trapeziectomy and flexor carpi radialis suspension. Journal of Hand Surgery: European Volume. 2007;32:462-6.
3. Thorkildsen RD, Røkkum M. Trapeziectomy with LRTI or joint replacement for CMC1 arthritis, a randomised controlled trial. J Plast Surg Hand Surg. 2019;53(6):361-9.
4. Hernández-Cortés P, Pajares-López M, Robles-Molina MJ, Gómez-Sánchez R, Toledo-Romero MA, De Torres-Urrea J. Two-year outcomes of Elektra prosthesis for trapeziometacarpal osteoarthritis: a longitudinal cohort study. J Hand Surg Eur Vol. 2012;37(2):130-7.
5. Vissers G, Goorens CK, Vanmierlo B, Bonte F, Mermuys K, Fils JF, et al. Ivory arthroplasty for trapeziometacarpal osteoarthritis: 10-year follow-up. J Hand Surg Eur Vol. 2019;44(2):138-45.
6. Cootjans K, Vanhaecke J, Dezillie M, Barth J, Pottel H, Stockmans F. Joint Survival Analysis and Clinical Outcome of Total Joint Arthroplasties With the ARPE Implant in the Treatment of Trapeziometacarpal Osteoarthritis With a Minimal Follow-Up of 5 Years. J Hand Surg Am. 2017;42(8):630-8.

For peer review only

7. Toffoli A, Teissier J. MAÏA Trapeziometacarpal Joint Arthroplasty: Clinical and Radiological Outcomes of 80 Patients With More than 6 Years of Follow-Up. J Hand Surg Am. 2017;42(10):838 e1- e8.
8. Dehl M, Chelli M, Lippmann S, Benaissa S, Rotari V, Moughabghab M. Results of 115 Rubis II reverse thumb carpometacarpal joint prostheses with a mean follow-up of 10 years. J Hand Surg Eur Vol. 2017;42(6):592-8.
9. Barrera-Ochoa S, Vidal-Tarrason N, Correa-Vázquez E, Reverte-Vinaixa MM, Font-Segura J, Mir-Bullo X. Pyrocarbon interposition (PyroDisk) implant for trapeziometacarpal osteoarthritis: minimum 5-year follow-up. J Hand Surg Am. 2014;39(11):2150-60.
10. van Cappelle HG, Deutman R, van Horn JR. Use of the Swanson silicone trapezium implant for treatment of primary osteoarthritis : long-term results. J Bone Joint Surg Am. 2001;83(7):999-1004.
11. Gangopadhyay S, McKenna H, Burke FD, Davis TR. Five- to 18-year follow-up for treatment of trapeziometacarpal osteoarthritis: a prospective comparison of excision, tendon interposition, and ligament reconstruction and tendon interposition. J Hand Surg Am. 2012;37(3):411-7.
12. Ganhewa AD, Wu R, Chae MP, Tobin V, Miller GS, Smith JA, et al. Failure Rates of Base of Thumb Arthritis Surgery: A Systematic Review. J Hand Surg Am. 2019;44(9):728-41 e10.
13. Wajon A, Carr E, Edmunds I, Ada L. Surgery for thumb (trapeziometacarpal joint) osteoarthritis. Cochrane Database Syst Rev. 2009(4):CD004631.
14. Billig JI, Nasser JS, Chung KC. National Prevalence of Complications and Cost of Small Joint Arthroplasty for Hand Osteoarthritis and Post-Traumatic Arthritis. J Hand Surg Am. 2020;45(6):553 e1- e12.
15. Makady A, van Veelen A, Jonsson P, Moseley O, D'Andon A, de Boer A, et al. Using Real-World Data in Health Technology Assessment (HTA) Practice: A Comparative Study of Five HTA Agencies. PharmacoEconomics. 2018;36(3):359-68.
16. Digital N. Hospital Episode Statistics. 2018 [Available from: https://digital.nhs.uk/data-and-information/data-tools-and-services/data-services/hospital-episode-statistics.
17. Kings Fund. The UK private health market. London: Kings Fund; 2014.
18. Digital N. Linked HES-ONS mortality data 2018 [Available from: https://digital.nhs.uk/data-and-information/data-tools-and-services/data-services/linked-hes-ons-mortality-data#ons-mortality-data.
19. Lane JCE SC, Green J, Lam WL, Furniss D, Sudlow C. Can we use routinely collected hospital and GP data for epidemiological study of common hand conditions? A UK Biobank based validation project. Biorxiv [Internet]. 2018.
20. Digital N. National clinical coding standards: OPCS-4 and ICD-10 London2017 [Available from: https://digital.nhs.uk/services/terminology-and-classifications/clinical-classifications.
21. WHO. International Classification of Disease version 10 2016 [2016:[Available from: https://www.who.int/classifications/icd/icdonlineversions/en/.
22. Ministry of Housing CaLG. English indices of Deprivation: UK Government; 2018 [Available from: https://www.gov.uk/government/collections/english-indices-of-deprivation.
23. Quan H, Sundararajan V, Halfon P, Fong A, Burnand B, Luthi JC, et al. Coding algorithms for defining comorbidities in ICD-9-CM and ICD-10 administrative data. Medical care. 2005;43(11):1130-9.
24. Quan H, Li B, Couris CM, Fushimi K, Graham P, Hider P, et al. Updating and validating the Charlson comorbidity index and score for risk adjustment in hospital discharge abstracts using data from 6 countries. American journal of epidemiology. 2011;173(6):676-82.
25. Digital N. NHS Outcomes Framework 2018 [Available from: https://digital.nhs.uk/data-and-information/publications/ci-hub/nhs-outcomes-framework.
26. ONS. Population estimates for the UK, England and Wales, Scotland and Northern Ireland 2018 [Available from: https://www.ons.gov.uk/peoplepopulationandcommunity/populationandmigration/populationestimates/datasets/populationestimatesforukenglandandwalesscotlandandnorthernireland.
27. Fine JP, Gray R.J. A proportional hazards model for the subdistribution of a competing risk. Journal of the American Statistical Association. 1999;94:496-509.
28. James Lind Alliance. Common Conditions Affecting the Hand and Wrist 2017 [Available from: http://www.jla.nihr.ac.uk/priority-setting-partnerships/common-conditons-affecting-the-hand-and-wrist/ [last accessed 27.04.2020].
29. ONS. Provisional long-term international migration estimates. 2020.
30. Mattila S, Waris E. Revision of trapeziometacarpal arthroplasty: risk factors, procedures and outcomes. Acta Orthop. 2019;90(4):389-93.

For peer review only - http://bmjopen.bmj.com/site/about/guidelines.xhtml

31. Wilkens SC, Xue Z, Mellema JJ, Ring D, Chen N. Unplanned Reoperation After Trapeziometacarpal Arthroplasty: Rate, Reasons, and Risk Factors. Hand (N Y). 2017;12(5):446-52.
32. Partridge TCJ, Baker PN, Jameson SS, Mason J, Reed MR, Deehan DJ. Conventional Versus Highly Cross-Linked Polyethylene in Primary Total Knee Replacement: A Comparison of Revision Rates Using Data from the National Joint Registry for England, Wales, and Northern Ireland. J Bone Joint Surg Am. 2020;102(2):119-27.
33. Penfold CM, Blom AW, Sayers A, Wilkinson JM, Hunt L, Judge A, et al. Understanding the uptake of new hip replacement implants in the UK: a cohort study using data from the National Joint Registry for England and Wales. BMJ Open. 2019;9(11):e029572.
34. BOA. Trauma and Orthopaedic Registries Unifying Structure (TORUS) 2020 [Available from: https://www.boa.ac.uk/policy-engagement/registries-and-outcomes/trauma-orthopaedic-registries-unifying-structure.html [last accessed 24.06.2020].
35. BSSH. UK Hand Registry 2018 [Available from: https://secure.amplitude-registry.com/BSSH/Account/Login?ReturnUrl=%2fBSSH.

For peer review only

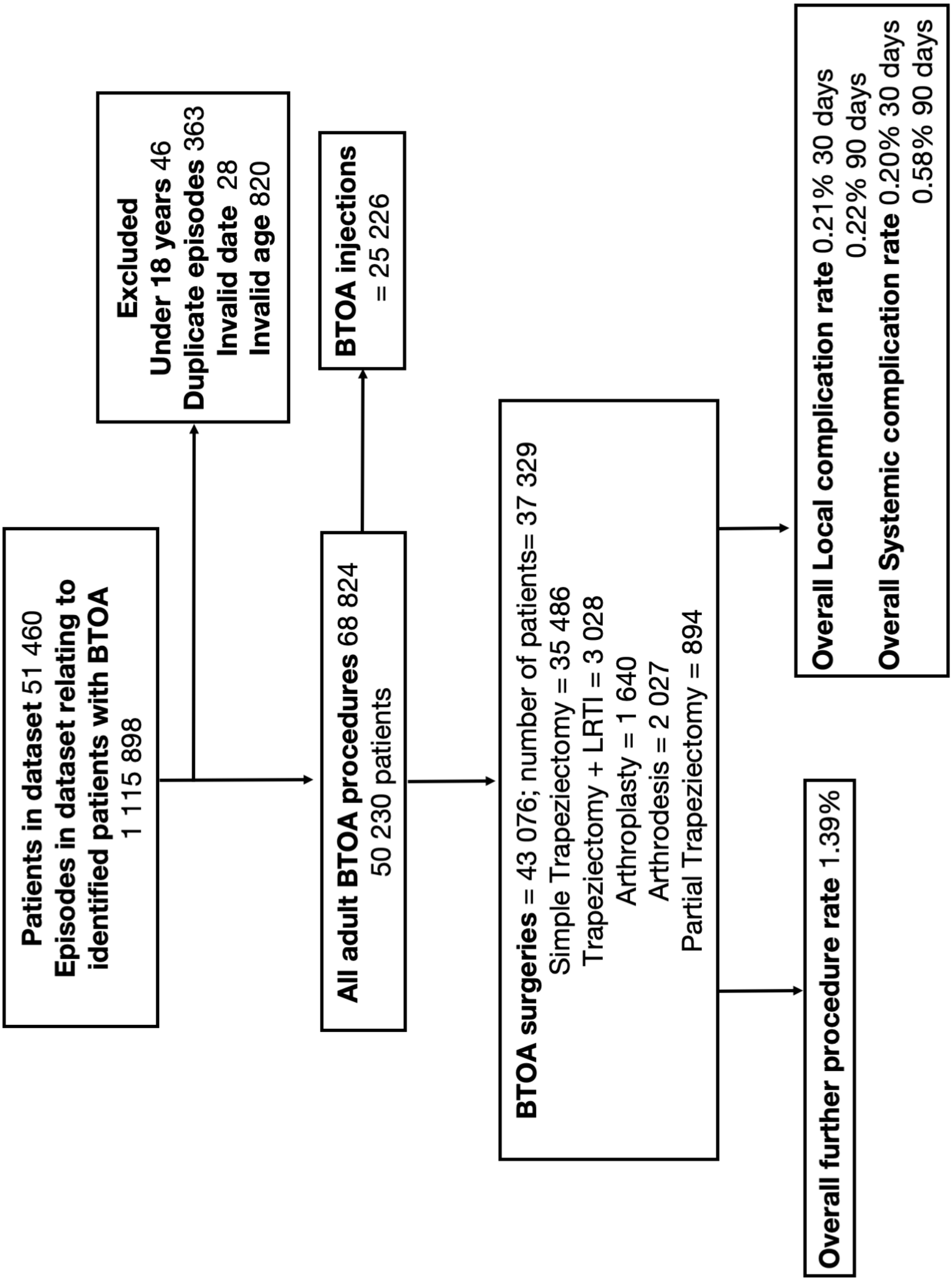
Patients in dataset 51 460
Episodes in dataset relating to identified patients with BTOA
1 115 898
Excluded
Under 18 years 46
Duplicate episodes 363
Invalid date 28
Invalid age 820
All adult BTOA procedures 68 824
50 230 patients
BTOA injections
= 25 226
BTOA surgeries = 43 076; number of patients= 37 329
Simple Trapeziectomy = 35 486
Trapeziectomy + LRTI = 3 028
Arthroplasty = 1 640
Arthrodesis = 2 027
Partial Trapeziectomy = 894
Overall Local complication rate 0.21% 30 days
0.22% 90 days
Overall Systemic complication rate 0.20% 30 days
0.58% 90 days
Overall further procedure rate 1.39%

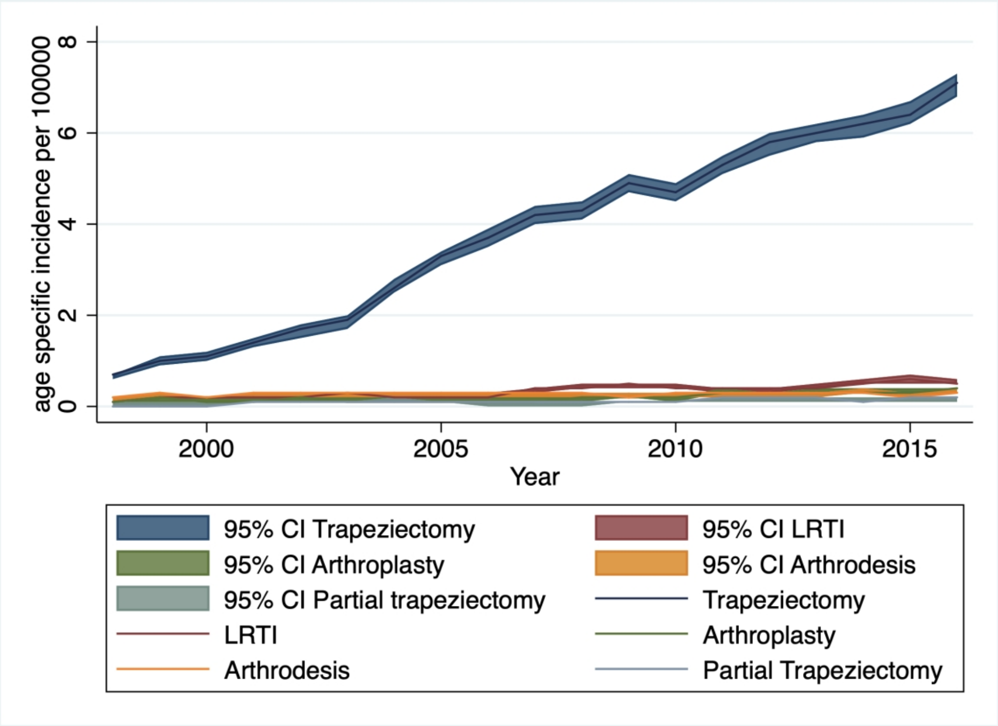

395x287mm (144 x 144 DPI)

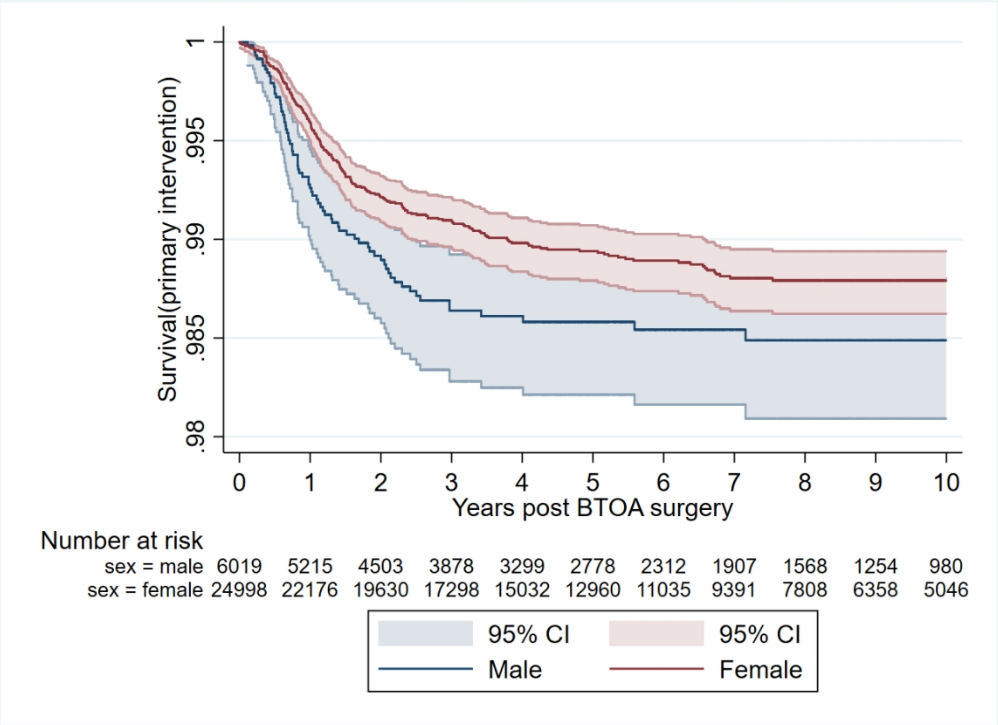

450x327mm (72 x 72 DPI)

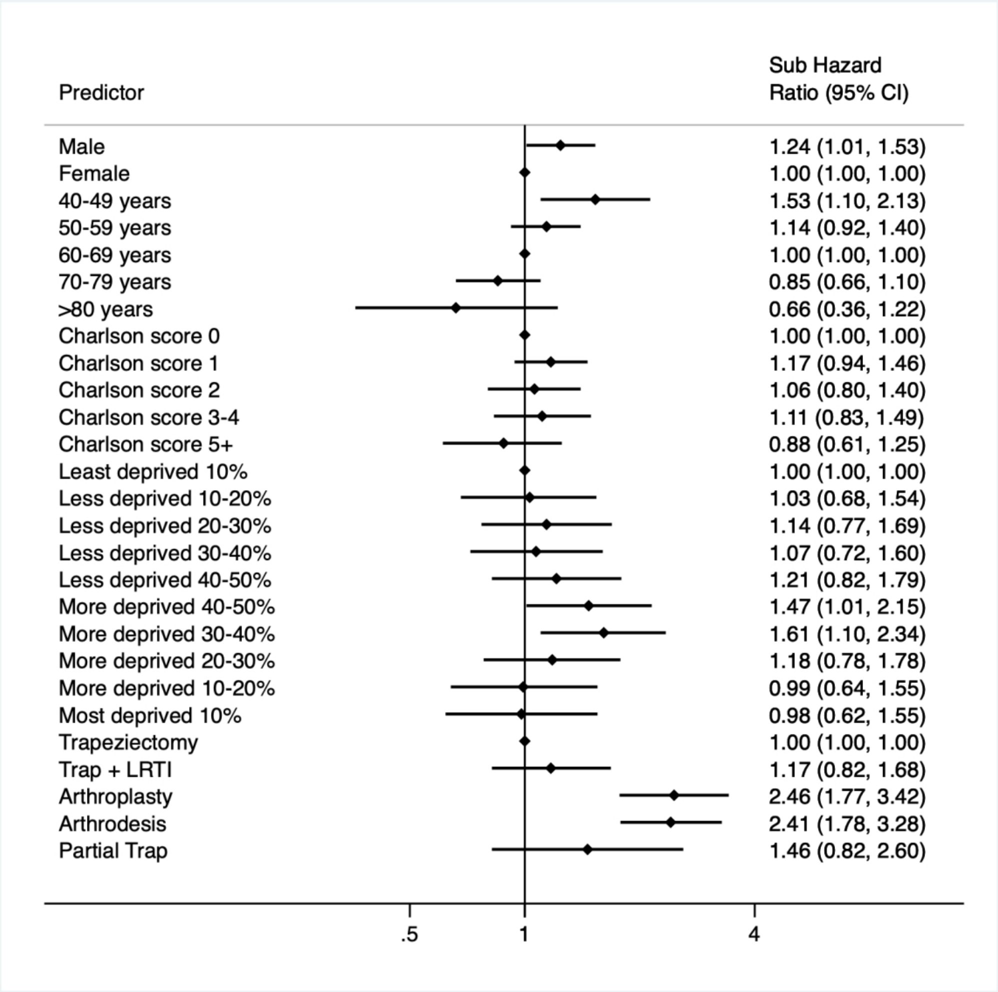

289x287mm (144 x 144 DPI)

Supplementary appendix Low rate of complications and further procedures following with BTOA surgery: analysis of national cohort of 43 076 surgeries with up to 19 years of follow up

Supplementary table 1 OPCS and ICD codes used to identify BTOA surgery

**BTOA OPCS codes**			**BTOA ICD-10 codes**
W57.2	alone		M18.0-18.9
W57.3	alone		M15.1-2
W57.8	**WITH ANY**	Z82.3	
W02.8		Z72.4	
W02.9		Z73.8	
W06.8		Z73.9	
W06.9		Z89.5	
W08.5		Z82.2	
W08.8		Z82.9	
W08.9			
W13.8			
W17.8			
W17.9			
W28.1			
W33.8			
W33.9			
W43.1			
W44.1			
W45.1			
W45.3			
W54.1			
W54.3			
W56.2			
W57.2			
W62.1			
W62.2			
W62.8			
W62.9			
W63.1			
W63.2			

For peer review only

W63.8		
W63.9		
W64.1		
W64.2		
W64.8		
W64.9		
W74.2		
W74.3		
W77.5		
W77.7		
W77.8		
W90.3		
W90.4		

Supplementary Table 2 Covariate codes

Covariates for BTOA	ICD-10 code
CTS	G560 (or OPCS A651)
Knee OA	M17
Generalised OA	M15; M19
Rheumatoid Arthritis	M05
Wrist/Hand fracture	S62
Oophorectomy	(OPCS Q22,Q23, Q43)

Supplementary Table 3 Complication codes for systemic complications and local complications

Complications	code
Wound dehiscence	OPCS Z82 + (S60.4 or S42.2 or S42.3 or S42.4)
Tendon repair	OPCS Z82 + T67
Wound debridement	OPCS Z82 + (T96.8 or T96.3 or T96.4)
Neurovascular injury	ICD code T81.2
Stroke	ICD I60X;I610-I619; I630-I639; I64X
Respiratory Tract Infection	ICD J12X J13, J14, J15X J180-J182 J188; J189; J22; J440 J441 J690 J691 J698 J851
Myocardial Infarction	ICD I210-I214; I219
Deep Vein Thrombosis/ Pulmonary Embolus	ICD I801-I803; I260; I269
Urinary tract infection/ acute renal failure	ICD N300 N390 N170-N172; N178 N179

For peer review only

Supplementary table 4 Baseline demographics of all patients undergoing BTOA surgery

	All surgery (N, %) (total=43 076)	Trapeziectomy N (%) (35 486)	LRTI N (%) (3028)	Arthroplasty N (%) (1640)	Arthrodesis N (%) (2027)	Partial Trapeziectomy N (%) (894)
**Sex**						
Male	8957 (20.7)	6 826 (19.2)	616 (20.3)	390 (23.8)	934 (46.1)	191 (21.3)
Female	34 112 (79.1)	28 655 (80.8)	2411 (79.6)	1250 (76.2)	1092 (53.9)	704 (78.7)
Missing	7 (0.02)	5 (0.01)	1 (0.03)	0	1 (0.05)	0
**Mean age (SD; years)**	63.2 (SD 9.2)	63.6 (SD 8.8)	62.5 (SD 9.7)	61.7 (SD 9.3)	58.1 (11.7)	63.1 (9.8)
**Charlson index**						
0	18 279 (42.4)	14 992 (42.3)	1 324 (43.8)	677 (41.3)	890 (43.9)	396 (44.3)
1	10 640 (24.7)	8 807 (24.8)	721 (23.8)	444 (27.1)	475 (23.4)	193 (21.6)
2	5 693 (13.2)	4 746 (13.4)	392 (13.0)	199 (12.1)	250 (12.3)	106 (11.8)
3	3 286 (7.6)	2 723 (7.7)	222 (7.3)	125 (7.6)	142 (7.0)	74 (8.3)
4	1 659 (3.9)	1 359 (3.8)	123 (4.1)	60 (3.7)	77 (3.8)	40 (4.5)
>=5	3 519 (8.2)	2 859 (8.0)	246 (8.1)	135 (8.2)	193 (9.6)	86 (8.5)
missing	0	0	0	0	0	
**IMD decile**						
Least deprived 10%	4 749 (11.0)	4 015 (11.3)	268 (8.8)	203 (12.4)	153 (7.6)	110 (12)
Less deprived 10-20%	4 769 (11.1)	3 934 (11.1)	314 (10.4)	232 (14.15)	195 (9.6)	94 (10)
Less deprived 20-30%	4 819 (11.2)	3 976 (11.2)	354 (11. 5)	185 (11.3)	212 (10.5)	92 (10)
Less deprived 30-40%	4 818 (11.2)	3 974 (11.2)	393 (13.0)	157 (9.6)	212 (10.5)	82 (9)
Less deprived 40-50%	4 682 (10.9)	3 853 (10.9)	345 (11.4)	154 (9.4)	221 (10.9)	109 (12)
More deprived 10-20%	3 279 (7.61)	2 700 (7.61)	218 (7.2)	110 (6.7)	181 (8.9)	70 (7)
More deprived 20-30%	3 798 (8.8)	3 108 (8.8)	273 (9.0)	145 (8.8)	202 (10.0)	70 (7)
More deprived 30-40%	4 206 (9.8)	3 442 (9.7)	303 (10.1)	164 (10.0)	215 (10.6)	82 (9)
More deprived 40-50%	4 559 (10.6)	3 700 (10.4)	354 (11.7)	172 (10.5)	241 (11.9)	92 (10)
Most deprived 10%	3039 (7.1)	2 529 (7.1)	160 (5.3)	110 (6.7)	152 (7.5)	88 (9)
Missing	358 (0.8)	255 (0.7)	46 (1.5)	8 (0.5)	43 (2.1)	<7*
**Ethnic group**						
Any white background	37 204 (86.4)	30 738 (86.6)	2628 (86.8)	1410 (86.0)	1640 (80.9)	755 (84.4)
Any Asian background	439 (1.0)	376 (1.1)	24 (0.8)	20 (1.2)	16 (0.8)	12 (1.3)
Any Black background	92 (0.2)	60 (0.2)	<7*	<7*	12 (0.6)	<7*
Any mixed background	60 (0.1)	46 (0.1)	<7*	<7*	<7*	<7*
Chinese	20 (0.1)	15 (0.05)	<7*	<7*	<7*	<7*
Any other ethnic group	155 (0.4)	130 (0.36)	9 (0.3)	<7*	<7*	<7*
Not stated	1237 (2.9)	3 467 (9.8)	301 (10.0)	134 (8.2)	299 (14.8)	88 (9.8)
Not known	3 869 (9.0)	654 (1.8)	58 (1.9)	26 (1.6)	51 (2.5)	28 (3.1)
Missing	0	0	0	0	0	0
	43 076	35 486	3028	1640	2027	895
**Co variates**						
Carpal Tunnel Syndrome	4 783	3 998 (11.2)	337 (10.9)	178 (10.9)	178 (8.8)	92 (10.3)
Knee Osteoarthritis	5 495	4 643 (13.0)	368 (11.9)	176 (10.7)	205 (10.1)	103 (11.5)
General Osteoarthritis	9 236	7 663 (21.4)	641 (20.8)	378 (23.0)	382 (18.8)	172 (19.2)
Rheumatoid Arthritis	109	81 (0.2)	7 (0.2)	7 (0.4)	12 (0.6)	2 (0.2)
Wrist Fracture	249	183 (0.5)	17 (0.6)	3 (0.2)	39 (1.9)	7 (0.8)
Oophorectomy	1 583	1 371 (3.8)	83 (2.7)	53 (3.2)	50 (2.5)	26 (2.9)

*Numbers less than 7 suppressed in line with NHS Digital disclosure control guidelines- percentages of other groups rounded to prevent secondary disclosure of data (29)

For peer review only

Supplementary Table 5 Comparison of patients with and without laterality (baseline demographics)

	**With laterality**	**Without laterality**
**Total**	36 072	7004
**Sex**		
Male	7592 (21.05)	1365 (19.49)
Female	28475 (78.94)	5637 (80.48)
Missing	5 (0.01)	2 (0.03)
**Mean age (SD; years)**	63.04 (9.15)	63.13 (9.26)
**Charlson index**		
0	15365 (42.60)	2914 (41.60)
1	8937 (24.78)	1703 (24.31)
2	4746 (13.16)	947 (13.52)
3	2730 (7.57)	556 (7.94)
4	1372 (3.80)	287 (4.10)
>=5	760 (8.10)	166 (8.52)
missing	0	0
**IMD decile**		
Least deprived 10%	4103 (11.37)	646 (9.22)
Less deprived 10-20%	4041 (11.20)	728 (10.39)
Less deprived 20-30%	4055 (11.24)	764 (10.91)
Less deprived 30-40%	4023 (11.15)	795 (11.35)
Less deprived 40-50%	3947 (10.94)	735 (10.49)
More deprived 10-20%	2681 (7.43)	598 (8.54)
More deprived 20-30%	3125 (8.66)	673 (9.61)
More deprived 30-40%	3487 (9.67)	719 (10.27)
More deprived 40-50%	3820 (10.59)	739 (10.55)
Most deprived 10%	2464 (6.83)	575 (8.21)
Missing	326 (0.90)	32 (0.46)
**Ethnic group**		
Any white background	31107 (86.24)	6097 (87)
Any Asian background	380 (1.05)	68 (1)
Any Black background	71 (0.2)	12 (<1)
Any mixed background	51 (0.14)	9 (<1)
Chinese	19 (0.05)	<7*
Any other ethnic group	127 (0.35)	28 (<1)
Not stated	3639 (10.08)	650 (9)
Not known	678 (1.88)	139 (2)
missing	0	0
**Co variates**		
Carpal Tunnel Syndrome	3989 (11.06)	794 (11.34)
Knee Osteoarthritis	4559 (12.64)	936 (13.36)
General Osteoarthritis	7569 (20.98)	1667 (23.8)
Rheumatoid Arthritis	84 (0.23)	25 (0.36)
Wrist Fracture	206 (0.57)	43 (0.61)
Oophorectomy	1351 (3.74)	232 (3.31)

*Numbers less than 7 suppressed in line with NHS Digital disclosure control guidelines- percentages of other groups rounded to prevent secondary disclosure of data (29)

For peer review only

Supplementary Table 6 Local and systemic complications per surgery subtype

	Time	All surgery N (%[95%CI])	Trapeziectomy N (%, 95%CI)	LRTI N (%, 95%CI)	Arthroplasty N (%, 95%CI)	Arthrodesis N (%, 95%CI)	Partial Trapeziectomy N (%, 95%CI)
**Local complications**							
Wound dehiscence and wound infection	Within 30 days	12(0.03%, [0.01-0.05])	10 (0.03% [0.01-0.05])	<7*	0	<7*	0
	Within 90 days	14(0.03% [0.02-0.05])	12 (0.03%[0.02-0.06])	<7*	0	<7*	0
Neurovascular injury	Within 30 days	79(0.18% [0.15-0.23])	68 (0.19% [0.15-0.24])	<7*	0	<7*	<7*
Any complication	Within 30 days	91(0.21% [0.17-0.26])	78 (0.22% [0.17-0.27])	<7*	0	<7*	<7*
	Within 90 days	93 (0.22% [0.17-0.26])	80 (0.23% [0.18-0.28])	<7*	0	<7*	<7*
**Systemic complications**							
Stroke	Within 30 days	9 (0.02% [0.01-0.04])	9 (0.03% [0.01-0.05])	0	0	0	0
	Within 90 days	40 (0.09%[0.07-0.13])	34 (0.10% [0.07-0.13])	<7*	<7*	<7*	<7*
Respiratory Tract infection	Within 30 days	70 (0.16% [0.13-0.21])	67 (0.19% [0.15-0.24])	<7*	0	0	<7*
	Within 90 days	174 (0.40% [0.35-0.47])	144 (0.41% [0.34-0.48])	10 (0.33% [0.16-0.61])	8 (0.49%[0.21-0.96]	<7*	<7*
Myocardial infarction	Within 30 days	17 (0.04%[0.02-0.06])	15 (0.04% [0.02-0.07])	<7*	0	0	0
	Within 90 days	50 (0.11% [0.09-0.15])	40 (0.11% [0.08-0.15])	<7*	0	<7*	<7*
DVT/PE	Within 30 days	18 (0.04% [0.02-0.07])	9 (0.03% [0.01-0.05])	<7*	<7*	0	0
	Within 90 days	41 (0.10% [0.07-0.13])	30 (0.08% [0.06-0.12])	<7*	<7*	0	<7*
UTI	Within 30 days	30 (0.07% [0.05-0.10]	25 (0.07% [0.05-0.10])	<7*	0	<7*	0
	Within 90 days	75 (0.17% [0.14-0.22])	62 (0.17% [0.13-0.22])	9 (0.30% [0.14-0.56])	0	<7*	<7*
Acute Renal Failure	Within 30 days	13 (0.03% [0.02-0.05])	9 (0.03% [0.01-0.05])	<7*	0	<7*	0
	Within 90 days	44 (0.10% [0.07-0.14])	40 (0.11% [0.08-0.15])	<7*	0	<7*	0
Any Systemic Complication	Within 30 days	87 (0.20% [0.16-0.25])	67 (0.19% [0.15-0.24])	10 (0.33%[0.16-0.61])	<7*	<7*	0
	Within 90 days	250 (0.58% [0.51-0.66])	206 (0.58% [0.50-0.67])	24 (0.79%[0.51-0.12])	<7*	<7*	<7*
Any Systemic Complication (excl UTI & ARF)	Within 30 days	44 (0.10% [0.07-0.14])	33 (0.10% [0.06-0.13])	<7*	<7*	0	0
	Within 90 days	131 (0.30%[0.25-0.36])	104 (0.29% [0.24-0.36])	13 (0.43%[0.23-0.73]	<7*	<7*	<7*

<7 Numbers less than 7 suppressed in line with NHS Digital disclosure control guidelines (29)

For peer review only

Supplementary Table 7 Risk of revision following any BTOA surgery Multivariable regression analysis accounting for competing risk of mortality (without surgical subtype as a risk in the model)

ALL SURGERIES	**Crude sHR[a]**	**95% CI**	**Adj sHR[a]**	**95% CI**
**Male**	1.22	0.99 to 1.49	1.24	1.01 to 1.52
**Age**				
40-49 years	1.53	1.11 to 2.12	1.53	1.10 to 2.13
50-59 years	1.12	0.91 to 1.38	1.14	0.92 to 1.40
60-69 years	1 (ref)	1 (ref)	1 (ref)	1 (ref)
70-79 years	0.86	0.66 to 1.10	0.85	0.66 to 1.10
>80 years	0.66	0.36 to 1.21	0.66	0.36 to 1.22
**Charlson comorbidity index**				
0	1 (ref)	1 (ref)	1 (ref)	1 (ref)
1	1.14	0.92 to 1.42	1.17	0.94 to 1.46
2	0.99	0.75 to 1.31	1.06	0.80 to 1.40
3+	0.92	0.73 to 1.17	1.01	0.78 to 1.30
**IMD**				
Least deprived 10%	1 (ref)	1 (ref)	1 (ref)	1 (ref)
Less deprived 10-20%	1.03	0.69 to 1.54	1.03	0.68 to 1.54
Less deprived 20-30%	1.16	0.78 to 1.72	1.14	0.77 to 1.70
Less deprived 30-40%	1.08	0.73 to 1.61	1.07	0.72 to 1.60
Less deprived 40-50%	1.24	0.84 to 1.83	1.21	0.82 to 1.79
More deprived 40-50%	1.04	0.66 to 1.62	0.99	0.63 to 1.55
More deprived 30-40%	1.24	0.82 to 1.86	1.18	0.79 to 1.78
More deprived 20-30%	1.66	1.14 to 2.42	1.61	1.10 to 2.34
More deprived 10-20%	1.51	1.04 to 2.20	1.47	1.01 to 2.15
Most deprived 10%	1.03	0.65 to 1.63	0.98	0.62 to 1.55
[a] Subhazard ratio				

For peer review only

Supplementary Table 8 Risk of revision following any BTOA surgery Multivariable regression analysis accounting for competing risk of mortality, including surgical subtype as a factor

ALL SURGERIES, including SURG SUBTYPE	**Crude sHR[a]**	**95% CI**	**Adj sHR[a]**	**95% CI**
**Male**	1.22	0.99 to 1.49	1.11	0.89 to 1.38
**Age**				
40-49 years	1.53	1.11 to 2.12	1.24	0.95 to 1.87
50-59 years	1.12	0.91 to 1.38	1.09	0.88 to 1.34
60-69 years	1 (ref)	1 (ref)	1 (ref)	1 (ref)
70-79 years	0.86	0.66 to 1.10	0.86	0.67 to 1.11
>80 years	0.66	0.36 to 1.21	0.67	0.37 to 1.24
**Charlson comorbidity index**				
0	1 (ref)	1 (ref)	1 (ref)	1 (ref)
1	1.14	0.92 to 1.42	1.17	0.94 to 1.46
2	0.99	0.75 to 1.31	1.05	0.79 to 1.40
3+	0.92	0.73 to 1.17	0.99	0.77 to 1.28
**IMD**				
Least deprived 10%	1 (ref)	1 (ref)	1 (ref)	1 (ref)
Less deprived 10-20%	1.03	0.69 to 1.54	1.00	0.67 to 1.50
Less deprived 20-30%	1.16	0.78 to 1.72	1.13	0.76 to 1.67
Less deprived 30-40%	1.08	0.73 to 1.61	1.06	0.71 to 1.58
Less deprived 40-50%	1.24	0.84 to 1.83	1.19	0.81 to 1.77
More deprived 40-50%	1.04	0.66 to 1.62	0.98	0.63 to 1.53
More deprived 30-40%	1.24	0.82 to 1.86	1.16	0.77 to 1.75
More deprived 20-30%	1.66	1.14 to 2.42	1.58	1.09 to 2.29
More deprived 10-20%	1.51	1.04 to 2.20	1.45	1.00 to 2.11
Most deprived 10%	1.03	0.65 to 1.63	0.96	0.61 to 1.52
**Surgery subtype**				
Simple trapeziectomy	1 (ref)	1 (ref)	1 (ref)	1 (ref)
Trapeziectomy + LRTI	1.22	0.86 to 1.73	1.16	0.82 to 1.66
Arthroplasty	2.51	1.81 to 3.48	2.49	1.80 to 3.44
Arthrodesis	2.55	1.91 to 3.40	2.40	1.78 to 3.26
Partial Trapeziectomy	1.44	0.81 to 2.55	1.46	0.82 to 2.59
[a] Subhazard ratio				

For peer review only

Supplementary Table 9 Risk of revision following Trapeziectomy Multivariable regression analysis accounting for competing risk of mortality

TRAPEZIECTOMY	**Crude sHR[a]**	**95% CI**	**Adj sHR[a]**	**95% CI**
**Male**	1.31	1.01 to 1.70	1.32	1.02 to 1.72
**Age**				
40-49 years	1.21	0.76 to 1.93	1.20	0.74 to 1.92
50-59 years	0.96	0.74 to 1.25	0.97	0.73 to 1.27
60-69 years	1 (ref)	1 (ref)	1 (ref)	1 (ref)
70-79 years	0.88	0.65 to 1.19	0.92	0.67 to 1.24
>80 years	0.53	0.23 to 1.19	0.55	0.24 to 1.25
**Charlson comorbidity index**				
0	1 (ref)	1 (ref)	1 (ref)	1 (ref)
1	0.96	0.73 to 1.26	0.97	0.73 to 1.28
2	0.80	0.56 to 1.15	0.79	0.54 to 1.15
3+	0.85	0.63 to 1.14	0.86	0.63 to 1.19
**IMD**				
Least deprived 10%	1 (ref)	1 (ref)	1 (ref)	1 (ref)
Less deprived 10-20	0.86	0.51 to 1.43	0.86	0.51 to 1.43
Less deprived 20-30%	1.03	0.63 to 1.68	1.02	0.63 to 1.67
Less deprived 30-40%	0.97	0.59 to 1.59	0.97	0.59 to 1.60
Less deprived 40-50%	1.13	0.70 to 1.82	1.12	0.69 to 1.81
More deprived 40-50%	0.89	0.50 to 1.57	0.89	0.50 to 1.57
More deprived 30-40%	1.01	0.60 to 1.70	1.00	0.59 to 1.69
More deprived 20-30%	1.68	1.07 to 2.64	1.68	1.07 to 2.63
More deprived 10-20%	1.59	1.01 to 2.49	1.59	1.01 to 2.49
Most deprived 10%	1.06	0.61 to 1.85	1.07	0.62 to 1.85
[a] Subhazard ratio				

For peer review only

Supplementary Table 10. Risk of revision following Trapeziectomy with LRTI
Multivariable regression analysis accounting for competing risk of mortality

LRTI	**Crude sHR**	**95% CI**	**Adjusted sHR[a]**	**95% CI**
**Male**	1.26	0.60 to 2.67	1.29	0.60 to 2.76
**Age**				
40-49 years	3.10	1.32 to 7.33	2.64	1.02 to 6.86
50-59 years	0.85	0.39 to 1.88	0.79	0.36 to 1.74
60-69 years	1 (ref)	1 (ref)	1 (ref)	1 (ref)
70-79 years	0.77	0.28 to 2.10	0.68	0.23 to 2.01
>80 years	0.77	0.10 to 5.83	0.81	0.10 to 6.28
**Charlson comorbidity index**				
0	1 (ref)	1 (ref)	1 (ref)	1 (ref)
1	1.14	0.55 to 2.34	1.04	0.50 to 2.13
2	0.79	0.29 to 2.09	0.85	0.31 to 2.31
3+	0.29	0.08 to 0.99	0.30	0.09 to 1.02
**IMD**				
Least deprived 10%	1 (ref)	1 (ref)	1 (ref)	1 (ref)
Less deprived 10-20%	0.40	0.04 to 4.39	0.39	0.04 to 4.26
Less deprived 20-30%	1.77	0.35 to 9.08	1.76	0.35 to 8.85
Less deprived 30-40%	1.39	0.25 to 7.64	1.34	0.24 to 7.33
Less deprived 40-50%	2.63	0.55 to 12.66	2.84	0.59 to 13.55
More deprived 40-50%	2.88	0.56 to 14.92	2.75	0.55 to 13.75
More deprived 30-40%	2.17	0.42 to 11.14	1.90	0.37 to 9.68
More deprived 20-30%	0.85	0.12 to 6.02	0.83	0.12 to 5.89
More deprived 10-20%	1.81	0.35 to 9.35	1.72	0.34 to 8.66
Most deprived 10%	1.52	0.22 to 10.73	1.22	0.17 to 8.92
[a] Subhazard ratio				

For peer review only

Supplementary Table 11. Risk of revision following BTOA Arthroplasty Multivariable regression analysis accounting for competing risk of mortality

Arthroplasty	**Crude sHR**	**95% CI**	**Adj sHR[a]**	**95% CI**
**Male**	0.66	0.31 to 1.40	0.72	0.33 to 1.60
**Age**				
40-49 years	0.92	0.32 to 2.69	1.07	0.37 to 3.10
50-59 years	0.75	0.37 to 1.48	0.80	0.40 to 1.59
60-69 years	1 (ref)	1 (ref)	1 (ref)	1 (ref)
70-79 years	0.83	0.36 to 1.89	0.76	0.31 to 1.84
>80 years	1.63	0.37 to 7.13	1.41	0.28 to 7.25
**Charlson comorbidity index**				
0	1 (ref)	1 (ref)	1 (ref)	1 (ref)
1	1.84	0.83 to 4.11	1.84	0.79 to 4.30
2	2.73	1.16 to 6.42	2.63	1.08 to 6.40
3+	2.47	1.12 to 5.43	2.14	0.88 to 5.24
**IMD**				
Least deprived 10%	1 (ref)	1 (ref)	1 (ref)	1 (ref)
Less deprived 10-20	1.77	0.45 to 6.89	1.71	0.44 to 6.62
Less deprived 20-30%	1.61	0.38 to 6.78	1.44	0.34 to 6.18
Less deprived 30-40%	3.23	0.87 to 12.00	2.96	0.78 to 11.22
Less deprived 40-50%	1.98	0.48 to 8.24	1.47	0.33 to 6.53
More deprived 40-50%	3.02	0.76 to 12.02	2.72	0.68 to 10.91
More deprived 30-40%	1.58	0.36 to 7.04	1.35	0.30 to 6.02
More deprived 20-30%	1.37	0.31 to 6.06	1.31	0.28 to 6.18
More deprived 10-20%	0.65	0.11 to 3.88	0.60	0.10 to 3.59
Most deprived 10%	1.91	0.42 to 8.65	1.22	0.24 to 6.30

[a] Subhazard ratio

For peer review only

Supplementary Table 12. Risk of revision following BTOA Arthrodesis Multivariable regression analysis accounting for competing risk of mortality

Arthrodesis	**Crude sHR**	**95% CI**	**Adj sHR[a]**	**95% CI**
**Male**	0.71	0.42 to 1.21	0.72	0.42 to 1.25
**Age**				
40-49 years	0.74	0.33 to 1.64	0.70	0.31 to 1.54
50-59 years	1 (ref)	1 (ref)	1 (ref)	1 (ref)
60-69 years	0.71	0.38 to 1.31	0.71	0.38 to 1.34
70-79 years	0.72	0.31 to 1.67	0.81	0.32 to 2.05
>80 years	0.51	0.07 to 3.72	0.54	0.08 to 3.86
**Charlson comorbidity index**				
0	1 (ref)	1 (ref)	1 (ref)	1 (ref)
1	2.07	1.10 to 3.88	2.18	1.16 to 4.10
2	1.36	0.60 to 3.12	1.48	0.62 to 3.55
3+	1.04	0.47 to 2.15	1.05	0.46 to 2.41
**IMD**				
Least deprived 10%	1 (ref)	1 (ref)	1 (ref)	1 (ref)
Less deprived 10-20	5.18	0.64 to 42.24	5.42	0.66 to 44.82
Less deprived 20-30%	4.06	0.49 to 33.89	4.16	0.49 to 35.07
Less deprived 30-40%	2.07	0.21 to 20.01	2.03	0.21 to 19.37
Less deprived 40-50%	4.09	0.49 to 34.21	3.93	0.46 to 33.33
More deprived 40-50%	1.69	0.15 to 18.84	1.62	0.14 to 18.29
More deprived 30-40%	6.59	0.83 to 52.31	6.50	0.79 to 53.61
More deprived 20-30%	10.00	1.30 to 76.46	9.77	1.26 to 75.72
More deprived 10-20%	6.05	0.76 to 48.20	5.92	0.72 to 49.01
Most deprived 10%	1.00	0.06 to 16.29	0.94	0.06 to 15.41
[a] Subhazard ratio				

For peer review only

Supplementary Figures 1-5 Kaplan Meier plots of risk of further procedure following

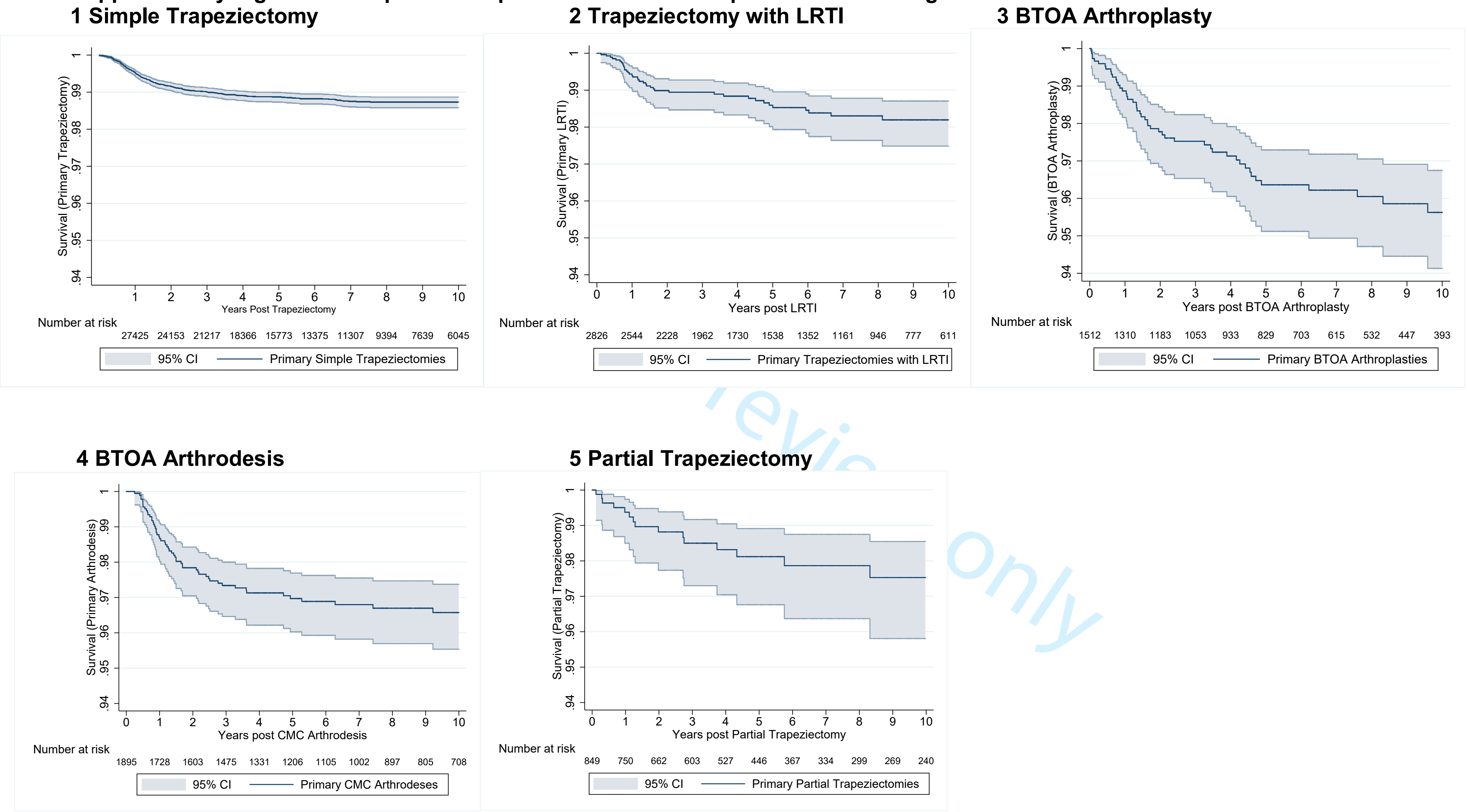

For peer review only - http://bmjopen.bmj.com/site/about/guidelines.xhtml

BMJ Open

Low rates of serious complications and further procedures following surgery for Base of Thumb Osteoarthritis: analysis of a national cohort of 43 076 surgeries

Journal:	*BMJ Open*
Manuscript ID	bmjopen-2020-045614.R1
Article Type:	Original research
Date Submitted by the Author:	11-Dec-2020
Complete List of Authors:	Lane, Jennifer; University of Oxford Nuffield Department of Orthopaedics Rheumatology and Musculoskeletal Sciences<br>Craig, Richard; University of Oxford, Nuffield Department of Orthopaedics, Rheumatology and Musculoskeletal Sciences<br>Rees, J L; University of Oxford, Nuffield Department of Orthopaedics, Rheumatology and Musculoskeletal Sciences<br>Gardiner, Matthew; University of Oxford Nuffield Department of Orthopaedics Rheumatology and Musculoskeletal Sciences; Wexham Park Hospital, Plastic Surgery<br>Mikhail, Mark; Oxford University Hospitals NHS Foundation Trust Nuffield Orthopaedic Centre, Plastic Surgery<br>Riley, Nicholas; Oxford University Hospitals NHS Foundation Trust Nuffield Orthopaedic Centre, Hand Surgery<br>Prieto-Alhambra, Daniel; university of oxford, ndorms<br>Furniss, Dominic ; University of Oxford, Nuffield Department of Orthopaedics Rheumatology and Musculoskeletal Science; Oxford University Hospitals NHS Foundation Trust Nuffield Orthopaedic Centre, Plastic Surgery
**Primary Subject Heading**:	Surgery
Secondary Subject Heading:	Epidemiology
Keywords:	SURGERY, Epidemiology < TROPICAL MEDICINE, Hand & wrist < ORTHOPAEDIC & TRAUMA SURGERY, Adult orthopaedics < ORTHOPAEDIC & TRAUMA SURGERY, RHEUMATOLOGY

SCHOLARONE™
Manuscripts

I, the Submitting Author has the right to grant and does grant on behalf of all authors of the Work (as defined in the below author licence), an exclusive licence and/or a non-exclusive licence for contributions from authors who are: i) UK Crown employees; ii) where BMJ has agreed a CC-BY licence shall apply, and/or iii) in accordance with the terms applicable for US Federal Government officers or employees acting as part of their official duties; on a worldwide, perpetual, irrevocable, royalty-free basis to BMJ Publishing Group Ltd ("BMJ") its licensees and where the relevant Journal is co-owned by BMJ to the co-owners of the Journal, to publish the Work in this journal and any other BMJ products and to exploit all rights, as set out in our licence.

The Submitting Author accepts and understands that any supply made under these terms is made by BMJ to the Submitting Author unless you are acting as an employee on behalf of your employer or a postgraduate student of an affiliated institution which is paying any applicable article publishing charge ("APC") for Open Access articles. Where the Submitting Author wishes to make the Work available on an Open Access basis (and intends to pay the relevant APC), the terms of reuse of such Open Access shall be governed by a Creative Commons licence – details of these licences and which Creative Commons licence will apply to this Work are set out in our licence referred to above.

Other than as permitted in any relevant BMJ Author's Self Archiving Policies, I confirm this Work has not been accepted for publication elsewhere, is not being considered for publication elsewhere and does not duplicate material already published. I confirm all authors consent to publication of this Work and authorise the granting of this licence.

For peer review only

Low rates of serious complications and further procedures following surgery for Base of Thumb Osteoarthritis: analysis of a national cohort of 43 076 surgeries

Jennifer C.E. Lane MRCS[1], Richard S. Craig DPhil FRCS (Tr&Orth)[1], Jonathan. L. Rees MD, FRCS (Tr&Orth) [1], Matthew D. Gardiner PhD, FRCS(Plast)[2,3], Mark Mikhail MRCS[4], Nicholas D Riley FRCS(Tr&Orth)[5], Daniel Prieto-Alhambra, DPhil[1*], Dominic Furniss, DM FRCS(Plast)[1,4*]

1. Oxford NIHR Musculoskeletal Biomedical Research Unit, Nuffield Department of Orthopaedics, Rheumatology and Musculoskeletal Sciences, Nuffield Orthopaedic Centre, University of Oxford, Windmill Road, Oxford, OX3 7LD, UK
2. Department of Plastic Surgery, Wexham Park Hospital, Slough, SL2 4HL, UK
3. Kennedy Institute of Rheumatology, Nuffield Department of Orthopaedics, Rheumatology and Musculoskeletal Sciences, Roosevelt Drive, Oxford, OX3 7FY, UK
4. Department of Plastic and Reconstructive Surgery, Nuffield Orthopaedic Centre, Windmill Road, Oxford, OX3 7HE, UK
5. Department of Hand Surgery, Nuffield Orthopaedic Centre, Windmill Road, Oxford, OX3 7HE, UK

* authors co-directed this work

Corresponding author: Professor Dominic Furniss, Nuffield Department of Orthopaedics, Rheumatology and Musculoskeletal Sciences, University of Oxford, Windmill Road, Oxford, OX3 7LD, UK dominic.furniss@ndorms.ox.ac.uk

Word Count (excl title page, abstract refs figures and tables): 3967

Keywords: base of thumb osteoarthritis, surgery, adverse event, revision, epidemiology

For peer review only - http://bmjopen.bmj.com/site/about/guidelines.xhtml

ABSTRACT

Objectives

To determine the incidence of further procedures and serious adverse events (SAE) requiring admission to hospital following elective surgery for base of thumb osteoarthritis (BTOA), and the patient factors associated with these outcomes.

Design

Population based cohort study

Setting

National Health Service (NHS) using the national Hospital Episode Statistics dataset linked to mortality records over a 19-year period (01/04/1998-31/03/2017).

Participants

43 076 primary surgeries were followed longitudinally in secondary care until death or migration on 37 329 patients over the age of 18 years.

Main Outcome measures

Incidence of further thumb base procedures (including revision surgery or intra-articular steroid injection) at any time post-operatively, and local wound and systemic complications (myocardial infarction, stroke, respiratory tract infection, venous thromboembolic events, urinary tract infection or renal failure) within 30 and 90 days. To identify patient factors associated with outcome, Fine and Gray model regression analysis was used to adjust for the competing risk of mortality in addition to age, overall comorbidity and socioeconomic status.

Results

Over the 19-years, there was an increasing trend in surgeries undertaken. The overall rate of further thumb base procedures after any surgery was 1.39%; the lowest rates after simple trapeziectomy (1.12%), the highest rates after arthroplasty (3.84%), and arthrodesis (3.5%). When matched for age, comorbidity and socioeconomic status, those undergoing arthroplasty and arthrodesis were 2.5 times more likely to undergo a further procedure (subhazard ratio 2.51 [95%CI 1.81-3.48]) and 2.55 [1.91-3.40]) than those undergoing simple trapeziectomy. Overall complication rates following surgery were 0.22% for serious local complications and 0.58% for systemic complications within 90 days of surgery.

Conclusions

The number of patients proceeding to BTOA surgery has increased over the last 19 years, with a low rate of further thumb base procedures and SAEs after surgery overall identified. Arthrodesis and arthroplasty had a significantly higher revision rate.

Trial Registration

Clinicaltrials.gov (NCT03573765)
NHS Digital Data Sharing Agreement (DARS-NIC-29827-Q8Z7Q)

For peer review only - http://bmjopen.bmj.com/site/about/guidelines.xhtml

STRENGTHS AND LIMITATIONS OF THIS STUDY

- To our knowledge, this is the largest scale national study identifying the risks following surgery for base of thumb osteoarthritis
- Long follow up is enabled by a nationalised health system linked to mortality records, that can identify surgical intervention in England over the last 19 years
- Using data from a nationalised health system has the benefit of capturing data from patients who present to another hospital for treatment of their complication
- As this study uses data collected from routine clinical care, results have a greater chance of being generalisable to a whole population
- This dataset can only identify complications presenting to hospital, so is limited to identifying serious adverse events requiring day case admission or further surgery.

For peer review only

INTRODUCTION

Background

Osteoarthritis of the first carpometacarpal joint, or base of thumb osteoarthritis (BTOA), is very common and may need surgical treatment.[1] Worldwide, the majority of surgical interventions are simple trapeziectomy with or without ligament reconstruction and tendon interposition (LRTI).[1-3] However, there is increasing interest in the use of arthroplasty for BTOA, with many different implants available.[4-10] Concerns have been raised regarding increased risk of complications with LRTI and arthroplasty compared with simple trapeziectomy.[11, 12] A Cochrane review comparing surgical techniques concluded that there was a high risk of bias within current studies, with no single technique presenting superior results.[13] Recent work aimed to identify complications within a large insurance provider in the US for small joint hand osteoarthritis procedures, and found complication rates as high as 35%, including 5% risk of a complication identified related to the prosthesis within two years of surgery.[14]

Many surgical studies in BTOA compare two techniques either within a clinical trial or single centre cohort, but there are very few studies comparing all available techniques with a long follow up at a national level. Routinely collected data produced from everyday clinical practice offers the advantage of enabling long-term follow up and detection of rare outcomes for healthcare interventions in the general population. [15] Routinely collected data for hospital admissions within the nationalised health service in England has been widely used to evaluate the safety and outcomes following surgery for other studies musculoskeletal conditions.[16-18]

The primary aim of this study was to estimate the incidence of BTOA surgery, further thumb base procedures and complications requiring admission to hospital in adults in the NHS in England. Secondary aims were to identify factors associated with adverse outcomes, including local and systemic complications, and further thumb base procedures after surgery. Our hypothesis was that there is variation in outcome between surgical subtypes.

METHODS

Data Source

A pseudonymised, bespoke extract was made from the NHS database Hospital Episode Statistics Admitted Patient Care (HES APC) covering the period 1st April 1998 to 31st March 2017).[19] This individual patient level data extract contained all patient episodes of care within the NHS England system, including those episodes that occur within independent providers that are remunerated by the NHS. The NHS provides the majority of healthcare in England, with only 13% of elective surgery estimated to be privately funded.[20] Episodes of care are linked via a patient's individual NHS number, therefore enabling linkage of all treatments undertaken and longitudinal follow up, including complications and revision surgeries undertaken by any provider. The HES APC extract was also linked to the ONS national mortality dataset prior to pseudonymization to identify cause and date of death.[21] By design, raw data was collected by NHS Digital prior to delivery to the research team.

Population

All patients over 18 years undergoing BTOA surgery were identified using a list of OPCS Classification of Interventions and Procedures (OPCS-4.8) to identify the procedures undertaken and International Classification of Diseases (ICD-10) codes to identify disease associated with the procedure(Supplementary tables 1-3).[22, 23]. OPCS codes are used in England to classify any procedure undertaken, and are used to identify a combination of the

For peer review only - http://bmjopen.bmj.com/site/about/guidelines.xhtml

implant and surgical subtype used in association with the anatomical area of the body where the surgery took place. For hand surgery, OPCS codes are used with combination of a generic surgical procedure codes and anatomical location.
All episodes of care for each individual prior to and following BTOA surgery were included in the extract. Patients were followed up until date of death or the end of the study period (31/03/2017). Patients who had an ICD-10 code for traumatic injury in the same episode as BTOA surgery were excluded, in order to only include patients undergoing elective surgery for longstanding BTOA. Duplicate episodes can occur over the change of financial year, and were removed during data cleaning. Each hand was considered as a separate surgical case. All cases of BTOA surgery identified within the 19 year period were included if they were not associated with a fracture ICD diagnosis code, and were not considered to be a duplicate episode. All cases were included with the aim of identifying the full national cohort within the time frame. Minimum follow up was set at one day, due to the clinical opinion of the team that further procedure or serious adverse events can occur very shortly after surgery (for example, acute carpal tunnel syndrome requiring decompression).

Validation: determining the feasibility of identifying surgical procedures and BTOA cases
2 Validation studies were undertaken in order to determine if it was possible to successfully identify surgical treatment of BTOA in this dataset, and secondly to determine if it was possible to identify the surgical subtypes undertaken.

Firstly, we undertook consensus discussion between surgeons, coders and NHS Digital to determine the most likely OPCS procedure codes and ICD diagnosis codes that would identify surgery for BTOA within routinely collected hospital data in England. This was an iterative process within a diverse team of stakeholders. Following this, an external validation study was then undertaken using this list of OPCS and ICD codes that identified a positive predictive value of 81% for incident BTOA with good interobserver reliability.[24]

Secondly, we undertook a further external validation study to determine if OPCS procedure codes could identify surgery subtypes within routinely collected data in the NHS in England. This was undertaken in our NHS trust using 2 blinded and independent reviewers (MM and NDR), who reviewed a year of surgical activity and the OPCS and ICD codes used during this time. In a year's sample of 104 patients undergoing BTOA surgery in our institution, we demonstrated a positive predictive value of 99% in identifying surgical subtype.

Covariates
Socioeconomic status of patients was identified using the Index of Multiple Deprivation (IMD), a government generated score of deprivation upon geographical location in England.[25] Overall comorbidity level was calculated using the Charlson Comorbidity Index (CCI). [26, 27] Ethnicity, as collected by NHS Digital, was included as a baseline covariate of interest, as national data is more inclusive than trial data, allowing us to identify any effect of ethnicity.[28] Baseline incidence of other conditions associated with BTOA pathogenesis in the literature were also identified using OPCS and ICD codes from previous episodes of secondary care, including a past medical history of carpal tunnel syndrome, knee osteoarthritis, generalized osteoarthritis, rheumatoid arthritis, wrist fracture, and oophorectomy.

Further thumb base procedures
Further thumb base procedure was defined as any revision surgery or intra-articular steroid injection in the same thumb base following surgery. In order to identify factors associated with further thumb base procedure, time to further procedure and produce an incidence rate, only further procedures identified with BTOA surgery or injection codes *in the same hand* were used, identified through a laterality code. Also, to produce an estimation of a 'worst case' scenario of the percentage of cases that *may* have proceeded to further thumb base

For peer review only - http://bmjopen.bmj.com/site/about/guidelines.xhtml

procedure, patients who had 3 or more episodes with BTOA surgery or injection codes but missing laterality were included in the percentage, but were not included in all subsequent analyses.

Complications
Complications were grouped into local (surgical site infection or dehiscence requiring surgery, and neurovascular or tendon injury) and systemic (stroke, respiratory tract infection, acute myocardial infarction, venous thromboembolic disease, urinary tract infection and acute renal failure). All complications were identified within HES APC and therefore require at least day case admission or surgery, so minor complications will not be recorded in the dataset. Complications were identified within 30 and 90 days of surgery to align with NHS outcomes framework used to compare outcomes between different medical conditions.[29] Furthermore, in the NHS, all surgical activity is compared with regards to outcomes within 30 and 90 days, and therefore exploring serious adverse events that occur within this timeframe enables comparison to other musculoskeletal conditions and limb surgeries. It was also our clinical experience that these serious local and systemic complications requiring hospital admission were likely to present within these time periods. Serious systemic complications were studied in addition to serious local complications due to concern about the side effects of general anaesthesia associated with upper limb procedures, and to enable comparison to rates identified following surgery for other musculoskeletal conditions.[17, 18]

Statistical methods
Age and sex specific incidence of surgery were calculated using official national mid-year population estimates in order to reflect the national nature of the healthcare system being studied, and to enable comparison to other musculoskeletal conditions, or study results from other countries.[30] Producing age and sex specific incidence also enables the results to take into account the change in population demographics over the long time period studied. All complications were calculated as a proportion of the sample with 95% confidence intervals. Incomplete records were found for 0.95% of cases for age, sex, ethnicity and IMD deciles. Data was assumed to be missing at random, and a complete case analysis undertaken without imputation. Laterality was missing in 16.2% of cases, and in order to assess if missing laterality caused a bias in included cases, the baseline demographics of patients with and without laterality were compared (supplementary table 4). No difference in baseline demographics was seen and therefore this was not considered as a potential cause of bias.

Factors associated with a further thumb base procedure were identified using a multivariable regression analysis using a Fine and Gray model that accounts for the competing risk of mortality.[31] The Fine and Gray model is a proportional hazards model that produces a subhazard ratio, that is derived from the cumulative incidence function (cause specific hazard). The Fine and Gray model splits the risk into two parts, the risk of further procedure, against (in this context) the risk of death. This is important within this study due to the long follow up and potential for patients to die within the follow up period, and allows the model to identify those who undergo a further thumb base procedure only within those who remain alive. Adjustment was undertaken for sex, age, socioeconomic status (using IMD), and overall comorbidity (using CCI). As complications were assessed within 90 days of surgery, multivariable cox proportional hazard modelling was used without adjusting for the competing risk of mortality. Patient age was identified as having a non-linear relationship with further thumb base procedure, and therefore was categorized, with the category containing the median age used as the reference category. When dividing the risk of further procedure by surgical subtype, there were insufficient patients below the age of 40 for inclusion in the multivariable regression analysis. Multivariable regression analysis was undertaken for all patient requiring further thumb base procedure, and also for those who had trapeziectomy alone, trapeziectomy with LRTI, arthroplasty and arthrodesis. For partial

For peer review only - http://bmjopen.bmj.com/site/about/guidelines.xhtml

trapeziectomy there were insufficient numbers of patients who had a further thumb base procedure to undertake this analysis.

In order to assess the impact of surgical subtype, the main multivariable regression analysis was undertaken to compare the risk of further thumb base procedure for each surgical subtype compared to simple trapeziectomy. Further sensitivity analyses were undertaken, where each surgical subtype was assessed as an individual cohort in separate multivariable models adjusted for age, sex and socioeconomic status. These were undertaken to investigate each cohort of patients undergoing a particular surgical procedure, to assess for the potential of confounding by indication within the main model where all patients were combined as one group.

Statistical analysis was undertaken using STATA version 15.1. Significance within the multivariable regression analysis was determined for a subhazard ratio (sHR) when a 95% confidence interval did not include 1, giving a more granular picture of the data than a p-value alone. No power calculation was undertaken as this study used all available data within a national cohort analysis.

PATIENT AND PUBLIC INVOLVEMENT

A James Lind Alliance priority setting partnership focused on common conditions affecting the hand and wrist also raised the question *'which interventions give the best results in the treatment of painful joints in the hand and wrist'* as important to patients, making this study a direct response in its research question.[32] No patients were directly involved in the study design, conduct or interpretation of the study.

RESULTS

Data processing

Figure 1 describes the data processing details. In the study period, 43 076 primary BTOA surgeries were performed on 37 329 individuals in English NHS hospitals. 79.1% were performed on female patients, and the mean age at primary surgery was 63 (SD 9.2). The median follow up time was 1835 days (interquartile range 816 to 3223). Only 345 cases (0.8%) had a follow up time of less than 30 days, and 1075 cases (2.5%) had a follow up time of less than 90 days. It was therefore considered that loss to follow up was not a sufficient source of bias within the cohort.

Demographics of patients undergoing BTOA surgery are shown in Table 1, with further granularity given in Supplementary Table 5. Over 60% of patients had no or very low levels of overall comorbidity, and 86% of patients were of white ethnic background. Patients were more likely to come from affluent sections of society. Approximately 1 in 5 patients had another hospital episode prior to BTOA surgery for osteoarthritis elsewhere in the body.

The most commonly undertaken surgical procedure was simple trapeziectomy, followed by trapeziectomy with LRTI. When comparing the baseline demographics of patients undergoing the different surgical subtypes, more men underwent arthrodesis and were slightly younger compared to the other groups. There appeared to be no difference in overall comorbidity as shown by Charlson index, and comparable socioeconomic status of patients shown by the index of multiple deprivation (IMD).

Table 1. Demographics of patients undergoing BTOA surgery

	All surgery (N, %) (total=43 076)	Trapeziectomy N (%) (35 486)	LRTI N (%) (3028)	Arthroplasty N (%) (1640)	Arthrodesis N (%) (2027)	Partial Trapeziectomy N (%) (894)
**Female sex**	34 112 (79.1)	28 655 (80.8)	2411 (79.6)	1250 (76.2)	1092 (53.9)	704 (78.7)

For peer review only

**Mean age (SD; years)**	63.2 (SD 9.2)	63.6 (SD 8.8)	62.5 (SD 9.7)	61.7 (SD 9.3)	58.1 (11.7)	63.1 (9.8)
**Charlson Comorbidity index**						
0	18 279 (42.4)	14 992 (42.3)	1 324 (43.8)	677 (41.3)	890 (43.9)	396 (44.3)
1	10 640 (24.7)	8 807 (24.8)	721 (23.8)	444 (27.1)	475 (23.4)	193 (21.6)
2	5 693 (13.2)	4 746 (13.4)	392 (13.0)	199 (12.1)	250 (12.3)	106 (11.8)
3	3 286 (7.6)	2 723 (7.7)	222 (7.3)	125 (7.6)	142 (7.0)	74 (8.3)
4	1 659 (3.9)	1 359 (3.8)	123 (4.1)	60 (3.7)	77 (3.8)	40 (4.5)
>=5	3 519 (8.2)	2 859 (8.0)	246 (8.1)	135 (8.2)	193 (9.6)	86 (8.5)
**Index of Multiple Deprivation (IMD)**						
Quintile 1 (least deprived)	9 518 (22.1)	7 949 (22.4)	582 (19.2)	435 (26.5)	348 (17.2)	204 (22.8)
Quintile 2	9 637 (22.4)	7 950 (22.4)	747 (24.6)	342 (20.9)	424 (21)	174 (19.5)
Quintile 3	7 961 (18.5)	6553 (18.5)	563 (18.6)	264 (16.1)	402 (19.8)	179 (20)
Quintile 4	8 004 (18.6)	6550 (18.5)	576 (19.1)	309 (18.8)	417 (20.6)	152 (17)
Quintile 5 (Most deprived)	7 598 (17.7)	6229 (17.5)	514 (17.0)	282 (17.2)	393 (19.4)	180 (20.1)
Missing	358 (0.8)	255 (0.7)	46 (1.5)	8 (0.5)	43 (2.0)	6 (0.7)

Trends in surgery

Over the 19-year period there was an increasing trend in the number of BTOA surgeries undertaken, predominantly accounted for by a steady increase in simple trapeziectomy, with minimal change in the incidence of other surgical procedures. (Figure 2)

Further Thumb Base Procedures

The overall rate of further thumb base procedure after any BTOA surgery was 1.39%, with the median time to intervention being under 1.5 years (Table 2; Figure 3). Whilst the rate of further thumb base procedures was low in general in this population, the highest rates were seen after arthroplasty (3.84%) and arthrodesis (3.50%) and the lowest following simple trapeziectomy (1.12%). The survival of BTOA surgery over time according to surgical subtype is given in Kaplan-Meier plots in the appendix (Supplementary Figures 1-5), noting the majority of patients proceeded to further intervention around the first 18 months post-operatively.

Table 2. Rate of further thumb base procedure (revision surgery or intraarticular steroid injection) according to BTOA surgical subtype

	Number of cases	Median follow up time in days (IQR)	Number cases needing further procedures[a] (%)	Median time to further procedure[a] in days (IQR)	Further procedure[a] rate per 1000 person years (95%CI)
All BTOA surgeries	43 076	1835 (816 to 3223)	599 (1.39%)	472 (272 to 965)	1.72 (1.54 to 1.92)
Trapeziectomy	35 486	1839 (820 to 3228)	398 (1.12%)	420.5 (248 to 804)	1.73 (1.55 to 1.93)
Trapeziectomy with LRTI	3 028	2063.5 (880 to 3405)	45 (1.49%)	508 (287 to 1648)	2.13 (1.56 to 2.92)
Arthroplasty	1 640	2000.5 (865 to 3683.5)	63 (3.84%)	732.5 (325 to 1666.5)	4.83 (3.65 to 6.40)
Arthrodesis	2 027	2712.5 (1222 to 4546)	71 (3.50%)	520 (301 to 1054)	3.91 (3.04 to 5.04)
Partial Trapeziectomy	894	1972 (929 to 4070)	22 (2.46%)	673.5 (296.5 to 1599.5)	2.63 (1.59 to 4.37)

[a] Further thumb base procedure only (intra-articular steroid injection or revision surgery)

For peer review only

Complications

Overall there was a low rate of serious local and systemic complications requiring admission to hospital following surgery, with the risk of any serious local complication being 0.22% within 90 days, and 0.58% for systemic complications within 90 days (Table 3; supplementary table 6). There was an insufficient number of cases to enable further analysis of factors associated with complications.

Table 3. Local and systemic complications following all BTOA surgery

	Time	All surgery N (%[95%CI])
**Local complications**		
Wound dehiscence and wound infection	Within 30 days	12 (0.03% [0.01-0.05])
	Within 90 days	14 (0.03% [0.02-0.05])
Neurovascular injury	Within 30 days	79 (0.18% [0.15-0.23])
Any complication	Within 30 days	91 (0.21% [0.17-0.26]
	Within 90 days	93 (0.22% [0.17-0.26]
**Systemic complications**		
Stroke	Within 30 days	9 (0.02% [0.01-0.04])
	Within 90 days	40 (0.09% [0.07-0.13])
Respiratory Tract infection	Within 30 days	70 (0.16% [0.13-0.21])
	Within 90 days	174 (0.40% [0.35-0.47])
Myocardial infarction	Within 30 days	17 (0.04% [0.02-0.06])
	Within 90 days	50 (0.11% [0.09-0.15])
DVT/PE	Within 30 days	18 (0.04% [0.02-0.07])
	Within 90 days	41(0.10% [0.07-0.13])
UTI	Within 30 days	30 (0.07% [0.05-0.10]
	Within 90 days	75 (0.17% [0.14-0.22])
Acute Renal Failure	Within 30 days	13 (0.03% [0.02-0.05])
	Within 90 days	44 (0.10% [0.07-0.14])
Any Systemic Complication	Within 30 days	87 (0.20% [0.16-0.25])
	Within 90 days	250 (0.58% [0.51-0.66])
Any Systemic Complication (excl UTI & ARF)	Within 30 days	44 (0.10% [0.07-0.14])
	Within 90 days	131 (0.30% [0.25-0.36])

Factors associated with further thumb base procedure

When considering the impact of age, sex, and sociodemographic factors, the risk of further thumb base procedure was increased for those in a younger age category of 40-49 years (adjusted subhazard ratio 1.53 [95%CI 1.10 to 2.13]) and for men (adjusted sHR 1.24 [1.01 to 1.53]) (Supplementary table 7) when adjusting for the other factors. When adjusting for surgical subtype in addition to age, sex and socioeconomic status to determine if surgical subtype had an impact on the risk of further procedure, the increased risk for men and those who underwent surgery at a younger age did not remain significant. This suggests that increased risk of further thumb base procedure was likely to be due to a greater proportion of younger male patients undergoing arthroplasty and arthrodesis (Supplementary table 8).

When adding in surgical subtype as a variable of interest into the main analysis, compared to those undergoing simple trapeziectomy, those undergoing arthroplasty and arthrodesis were around 2.5 times more likely to proceed to further thumb base procedure (arthroplasty 2.46 [1.77 to 3.42]; arthrodesis 2.41 [1.78 to 3.28]) after adjustment for age, sex and socioeconomic deprivation. The forest plot in Figure 4 shows the impact of age, sex, socioeconomic deprivation and surgical subtype seen upon the risk of undergoing a further procedure.

For peer review only - http://bmjopen.bmj.com/site/about/guidelines.xhtml

In the sensitivity analyses where each surgical subtype was run in a separate model adjusted for age sex and deprivation, for the risk of further thumb base procedure within each surgical subtype, an increased risk of further procedure was seen in men for those who underwent trapeziectomy alone when adjusted for age and deprivation (adjusted sHR 1.33 [1.02 to 1.73]). This was not seen in those undergoing LRTI, arthrodesis or arthroplasty, but noting there were fewer cases in these subgroups. Age and sociodemographic status did not appear significant in any of the surgical subgroups in regression analysis. (Supplementary tables 9-12).

DISCUSSION

Principle Findings

This study found that patients undergoing surgery for BTOA in the NHS in England are predominantly females and in their 7th decade. There has been an increase in surgery undertaken over the last 19 years. There was a low rate of revision surgery and intraarticular steroid injection identified following primary surgery in this cohort. In addition, there are low rates of serious local and systemic complications requiring hospital admission identified within the immediate post-operative period. Further thumb base procedures mostly occurred within the first 18 months post operatively. The greatest factor influencing progression to further thumb base procedure in this population was surgical subtype, with patients receiving an arthroplasty around 2.5 times more likely to undergo further procedure than those having simple trapeziectomy.

Strengths and contribution to current knowledge

The major strength of this study is the national nature of the study setting. The HES APC dataset has been extensively used for evaluating the safety and outcomes following surgery for other musculoskeletal conditions, and this large national cohort of patients provides longitudinal follow up of individuals across multiple providers.[16-18] It allows retention in the study of those individuals who may have sought a second opinion at a different hospital in England. This gives a better overview of the need for revision surgery and for the management of serious adverse events following surgery than is possible in single centre or regional studies. As the private healthcare sector accounts for a low proportion of all healthcare activity in England, whilst some may seek consultation in this sector, all NHS activity will be captured within this dataset.[20] It also accounts for patient movement within the country, noting that emigration out of the UK is thought to be low at around 400,000 persons per year.[33]

This study includes all adult patients who underwent surgery in England, and is representative of the generality of hand surgery practice in a large public healthcare system. Patients at extremes of ages, with comorbid conditions that may render them ineligible to take part in clinical trials, are included. Due to practical and financial constraints, randomised control trials typically lack the statistical power and longevity of follow up to reliably inform on the safety of surgical procedures for BTOA. Large observational studies, such as this one, are important to fill that evidence gap. Previous studies have suggested increased revision risk in those of a younger age which we also detected here in a univariable analysis. The effect did not persist here in multivariable analysis, and may therefore be more directly related to choice of surgery type.[34, 35]

All widely used surgical procedure subtypes are included in this study, enabling a full comparison of each main procedure with the other. This facilitates broad comparison of the rate of complications and revision across an inclusive population with longer follow up than most clinical trials.[12, 13] This study emphasises that whilst the overall risk of BTOA surgery is low, there is an increased risk of further procedure for those undergoing arthroplasty that warrants further large scale epidemiological analysis, as has been seen in joint arthroplasty research at other anatomical sites.[36, 37]

For peer review only - http://bmjopen.bmj.com/site/about/guidelines.xhtml

Limitations and future work

This study focusses on the patient factors that can impact the risk of adverse outcome. It does not account for potential variation associated with surgeon or hospital factors. Further methodological research is underway to identify the best method of multilevel analysis that can explore the interaction of healthcare provider factors with patient factors. This technique may enable us to identify how multiple factors influence the risk of adverse events. Patients included here are those who have been identified by NHS Digital records, a dataset that is designed for remuneration rather than research.

Care was taken to validate the codes in two different populations used to identify patient records in order to limit misclassification bias, but this remains a potential limitation of the work. All arthroplasties are coded as a generic arthroplasty code, and therefore implant type cannot be analysed. Future work focussing on implant registries, such as currently used in hip and knee arthroplasty, is needed to determine the impact of implant type upon outcome in routine clinical practice.[38, 39] Members of our Department recently undertook a service evaluation project in 15 centres in the UK. [40] In this study of 150 patients, there was a 50:50 split between LRTI and simple trapeziectomy undertaken. However, this was undertaken in centres volunteering to participate in research, with data collected by surgeons, with a much smaller sample size and over a much shorter period of time (March 2017- May 2019). This study may therefore be prone to selection bias in a different way to the selection bias generated by using routinely collected data. In order to determine if we could adequately identify surgical subtypes from the HES APC dataset, we undertook two external validation studies after an iterative process of generating an included list of diagnosis and procedure codes. After these processes, we demonstrated that it was possible to differentiate between the surgical subtypes, including simple trapeziectomy and LRTI. Our data suggest that simple trapeziectomy was undertaken much more commonly than LRTI in England. Randomised control trials based in the UK and a Cochrane review have suggested little difference in efficacy between LRTI and simple trapeziectomy, and higher costs and complications associated with LRTI, and therefore the low proportion of LRTIs undertaken could reflect English surgeons adhering to UK evidence based practice.[2, 11, 13]

Comorbidities registered in this dataset are necessarily identified within an admitted patient episode, which may lead to inclusion bias of information used for remuneration. As the registration of comorbidities is likely to reflect the selection bias of only those who have received inpatient care for these conditions, these conditions were not used as factors that may influence the need for further thumb base procedures within the multivariable analysis. Whilst admitted patient care gives the best granularity of information for surgery and in-hospital events, it does not cover interactions occurring in an outpatient or primary care environment. Therefore, this study only identifies serious adverse events; those requiring at least a day case admission to hospital, and will not include those complication seen in primary care or outpatient facilities. It will fail to identify some pre-existing comorbidities in our patients, or persistent pain. Complications treated in the community such as minor surgical site infections requiring oral antibiotics will also not be identified using this data. Our future work is focussed upon identifying patients who have undergone BTOA surgery in primary care records, to compare the information collected there regarding comorbidity and post-operative infection.

Finally, this study uses revision surgery and serious adverse events as a proxy for outcome following surgery, as no patient reported outcomes (PROMS) are routinely collected for hand surgery at a national level by the NHS. Further work is needed to focus upon comparing PROMS for each surgical subtype in routine clinical practice to identify the quality of outcome from the patient perspective.

For peer review only - http://bmjopen.bmj.com/site/about/guidelines.xhtml

Meaning and use for clinical practice

As this study includes a national cohort of patients from routine clinical practice, the rates of further thumb base procedure and serious adverse events requiring in patient hospital treatment given in this study are directly of use for counselling patients during the process of consent and shared decision making. The higher rates of further thumb base procedure following arthroplasty found in this study concurs with a previous systematic review of smaller trials.[12] Within a large cohort of patients, demographic factors did not appear to be associated with significantly increased risk of adverse outcomes when accounting for surgical subtype, which again is informative for both the surgeon and patient. The study suggests that there is a low overall rate of further procedures and serious complications following surgery for BTOA. Whilst the risk of further procedure does vary by surgical subtype, demographic factors do not appear to influence this risk.

FIGURE LEGENDS

Figure 1 Data processing flow chart
Figure 2 Age specific incidence of BTOA surgeries by year, England 1998-2017
Figure 3 Kaplan Meier plot of risk of further thumb base procedure after all BTOA surgery, divided by sex
Figure 4 Forest plot of relative risk of further thumb base procedure after primary BTOA surgery within multivariable regression analysis adjusted for age, sex, sociodemographic status and surgical subtype

ETHICAL APPROVAL

This study was registered at clinicaltrials.gov (NCT03573765) and approved by the University Research Services Clinical Trials Research Group (project ID 12787), and the NHS Data Access Advisory Group (DAAG). It was carried out in accordance with the NHS Digital Data Sharing Agreement (DARS-NIC-29827-Q8Z7Q). Studies using non-identifiable records from Hospital Episode Statistics are exempt from research ethics committee approval. Patients have the right to request that their data is not released by NHS Digital for use by researchers (register a “Type 2 opt-out”).

CONTRIBUTOR STATEMENT

JCEL, DF, RC, JLR, MG and DPA conceived the study and designed the study method. MM and NDR undertook the validation study. JCEL and RC acquired the data. JCEL performed the data analysis. JCEL, DF, RC, JLR, MG, NDR and DPA interpreted the data. JCEL drafted the manuscript. All authors reviewed and edited the final manuscript. JCEL, RC, JR and DF has full access to all data in the study. DF is the guarantor.

ACKNOWLEDGEMENTS

The authors would like to acknowledge Miss Rachel Kuo and Professor Jane Green for their work in this study.

ROLE OF THE FUNDING SOURCE

This work was supported by Versus Arthritis [21605] (JL); the Medical Research Council [MR/K501256/1] (JL); RCS England/ NJR research fellowship (RC); University of Oxford (JR); the National Institute for Health Research (NIHR) [SRF-2018-11- ST2-004] (DPA); NIHR Biomedical Research Centre, Oxford (BRC) (RC, DF, JR) and Oxford Medical Research Fund (MDG). No funders had a direct role in this study. The views and opinions expressed are those of the authors and do not necessarily reflect those of the Clinician Scientist Award programme, NIHR, NHS or the Department of Health. This work used data supplied by NHS Digital copyright 2018, re-used with the permission of NHS Digital. All rights reserved.

COMPETING INTERESTS

For peer review only

For peer review only - http://bmjopen.bmj.com/site/about/guidelines.xhtml

All authors have completed an ICJME conflict of interest form that is uploaded with the study (http://www.icmje.org/conflicts-ofinterest/) no support from any organization for the submitted work; D P-A has received research grants from Amgen, Servier, UCB; departmental fees for speaker services from Amgen, departmental fees for consultancy from UCB.JL reports grants from the Medical Research Council (MR/K501256/1) and Versus Arthritis (21605), outside of the submitted work. NR reports personal fees from Acumed, outside the submitted work; Dr. Craig reports grants from Royal College of Surgeons/National Joint Registry, outside the submitted work. No other relationships or activities could appear to have influenced the submitted work.

TRANSPARENCY STATEMENT

The lead author affirms that the manuscript is an honest, accurate, and transparent account of the study being reported; no important aspects of the study have been omitted, and that any discrepancies from the study as originally planned have been explained.

DATA SHARING STATEMENT

No further data can be made available due to NHS Digital restrictions. Data extracts can be applied for via the NHS Digital data access request service (https://digital.nhs.uk/services/data-access-request-service-dars).

EXECUTIVE LICENSE

The Corresponding Author has the right to grant on behalf of all authors and does grant on behalf of all authors, a worldwide licence (http://www.bmj.com/sites/default/files/BMJ%20Author%20Licence%20March%202013.doc) to the Publishers and its licensees in perpetuity, in all forms, formats and media (whether known now or created in the future), to i) publish, reproduce, distribute, display and store the Contribution, ii) translate the Contribution into other languages, create adaptations, reprints, include within collections and create summaries, extracts and/or, abstracts of the Contribution and convert or allow conversion into any format including without limitation audio, iii) create any other derivative work(s) based in whole or part on the on the Contribution, iv) to exploit all subsidiary rights to exploit all subsidiary rights that currently exist or as may exist in the future in the Contribution, v) the inclusion of electronic links from the Contribution to third party material where-ever it may be located; and, vi) licence any third party to do any or all of the above. All research articles will be made available on an open access basis (with authors being asked to pay an open access fee— see http://www.bmj.com/about-bmj/resources-authors/forms-policies-and-checklists/copyright-open-access-and-permission-reuse). The terms of such open access shall be governed by a Creative Commons licence—details as to which Creative Commons licence will apply to the research article are set out in our worldwide licence referred to above.

REFERENCES

1. Yuan F, Aliu O, Chung KC, Mahmoudi E. Evidence-Based Practice in the Surgical Treatment of Thumb Carpometacarpal Joint Arthritis. J Hand Surg Am. 2017;42(2):104-12 e1.
2. Field J, Buchanan D. To suspend or not to suspend: a randomised single blind trial of simple trapeziectomy versus trapeziectomy and flexor carpi radialis suspension. Journal of Hand Surgery: European Volume. 2007;32:462-6.
3. Thorkildsen RD, Røkkum M. Trapeziectomy with LRTI or joint replacement for CMC1 arthritis, a randomised controlled trial. J Plast Surg Hand Surg. 2019;53(6):361-9.
4. Hernández-Cortés P, Pajares-López M, Robles-Molina MJ, Gómez-Sánchez R, Toledo-Romero MA, De Torres-Urrea J. Two-year outcomes of Elektra prosthesis for trapeziometacarpal osteoarthritis: a longitudinal cohort study. J Hand Surg Eur Vol. 2012;37(2):130-7.
5. Vissers G, Goorens CK, Vanmierlo B, Bonte F, Mermuys K, Fils JF, et al. Ivory arthroplasty for trapeziometacarpal osteoarthritis: 10-year follow-up. J Hand Surg Eur Vol. 2019;44(2):138-45.
6. Cootjans K, Vanhaecke J, Dezillie M, Barth J, Pottel H, Stockmans F. Joint Survival Analysis and Clinical Outcome of Total Joint Arthroplasties With the ARPE Implant in the Treatment of

Trapeziometacarpal Osteoarthritis With a Minimal Follow-Up of 5 Years. J Hand Surg Am. 2017;42(8):630-8.
7. Toffoli A, Teissier J. MAÏA Trapeziometacarpal Joint Arthroplasty: Clinical and Radiological Outcomes of 80 Patients With More than 6 Years of Follow-Up. J Hand Surg Am. 2017;42(10):838 e1- e8.
8. Dehl M, Chelli M, Lippmann S, Benaissa S, Rotari V, Moughabghab M. Results of 115 Rubis II reverse thumb carpometacarpal joint prostheses with a mean follow-up of 10 years. J Hand Surg Eur Vol. 2017;42(6):592-8.
9. Barrera-Ochoa S, Vidal-Tarrason N, Correa-Vázquez E, Reverte-Vinaixa MM, Font-Segura J, Mir-Bullo X. Pyrocarbon interposition (PyroDisk) implant for trapeziometacarpal osteoarthritis: minimum 5-year follow-up. J Hand Surg Am. 2014;39(11):2150-60.
10. van Cappelle HG, Deutman R, van Horn JR. Use of the Swanson silicone trapezium implant for treatment of primary osteoarthritis : long-term results. J Bone Joint Surg Am. 2001;83(7):999-1004.
11. Gangopadhyay S, McKenna H, Burke FD, Davis TR. Five- to 18-year follow-up for treatment of trapeziometacarpal osteoarthritis: a prospective comparison of excision, tendon interposition, and ligament reconstruction and tendon interposition. J Hand Surg Am. 2012;37(3):411-7.
12. Ganhewa AD, Wu R, Chae MP, Tobin V, Miller GS, Smith JA, et al. Failure Rates of Base of Thumb Arthritis Surgery: A Systematic Review. J Hand Surg Am. 2019;44(9):728-41 e10.
13. Wajon A, Carr E, Edmunds I, Ada L. Surgery for thumb (trapeziometacarpal joint) osteoarthritis. Cochrane Database Syst Rev. 2009(4):CD004631.
14. Billig JI, Nasser JS, Chung KC. National Prevalence of Complications and Cost of Small Joint Arthroplasty for Hand Osteoarthritis and Post-Traumatic Arthritis. J Hand Surg Am. 2020;45(6):553 e1- e12.
15. Makady A, van Veelen A, Jonsson P, Moseley O, D'Andon A, de Boer A, et al. Using Real-World Data in Health Technology Assessment (HTA) Practice: A Comparative Study of Five HTA Agencies. PharmacoEconomics. 2018;36(3):359-68.
16. Abram SGF, Judge A, Beard DJ, Price AJ. Adverse outcomes after arthroscopic partial meniscectomy: a study of 700 000 procedures in the national Hospital Episode Statistics database for England. Lancet. 2018;392(10160):2194-202.
17. Craig RS, Lane JCE, Carr AJ, Furniss D, Collins GS, Rees JL. Serious adverse events and lifetime risk of reoperation after elective shoulder replacement: population based cohort study using hospital episode statistics for England. BMJ (Clinical research ed). 2019;364:l298.
18. Alser O, Craig RS, Lane JCE, Prats-Uribe A, Robinson DE, Rees JL, et al. Serious complications and risk of re-operation after Dupuytren's disease surgery: a population-based cohort study of 121,488 patients in England. Sci Rep. 2020;10(1):16520.
19. Digital N. Hospital Episode Statistics. 2018 [Available from: https://digital.nhs.uk/data-and-information/data-tools-and-services/data-services/hospital-episode-statistics.
20. Kings Fund. The UK private health market. London: Kings Fund; 2014.
21. Digital N. Linked HES-ONS mortality data 2018 [Available from: https://digital.nhs.uk/data-and-information/data-tools-and-services/data-services/linked-hes-ons-mortality-data#ons-mortality-data.
22. WHO. International Classification of Disease version 10 2016 [2016:[Available from: https://www.who.int/classifications/icd/icdonlineversions/en/.
23. Digital N. National clinical coding standards: OPCS-4 and ICD-10 London2017 [Available from: https://digital.nhs.uk/services/terminology-and-classifications/clinical-classifications.
24. Lane JCE SC, Green J, Lam WL, Furniss D, Sudlow C. Can we use routinely collected hospital and GP data for epidemiological study of common hand conditions? A UK Biobank based validation project. Biorxiv [Internet]. 2018.
25. Ministry of Housing CaLG. English indices of Deprivation: UK Government; 2018 [Available from: https://www.gov.uk/government/collections/english-indices-of-deprivation.
26. Quan H, Sundararajan V, Halfon P, Fong A, Burnand B, Luthi JC, et al. Coding algorithms for defining comorbidities in ICD-9-CM and ICD-10 administrative data. Medical care. 2005;43(11):1130-9.
27. Quan H, Li B, Couris CM, Fushimi K, Graham P, Hider P, et al. Updating and validating the Charlson comorbidity index and score for risk adjustment in hospital discharge abstracts using data from 6 countries. American journal of epidemiology. 2011;173(6):676-82.
28. Digital N. Hospital Episode Statistics Data Dictionary. In: Care HaS, editor. 2020.
29. Digital N. NHS Outcomes Framework 2018 [Available from: https://digital.nhs.uk/data-and-information/publications/ci-hub/nhs-outcomes-framework.

For peer review only - http://bmjopen.bmj.com/site/about/guidelines.xhtml

30. ONS. Population estimates for the UK, England and Wales, Scotland and Northern Ireland 2018 [Available from: https://www.ons.gov.uk/peoplepopulationandcommunity/populationandmigration/populationestimates/datasets/populationestimatesforukenglandandwalesscotlandandnorthernireland.
31. Fine JP, Gray R.J. A proportional hazards model for the subdistribution of a competing risk. Journal of the American Statistical Association. 1999;94:496-509.
32. James Lind Alliance. Common Conditions Affecting the Hand and Wrist 2017 [Available from: http://www.jla.nihr.ac.uk/priority-setting-partnerships/common-conditons-affecting-the-hand-and-wrist/ [last accessed 27.04.2020].
33. ONS. Provisional long-term international migration estimates. 2020.
34. Mattila S, Waris E. Revision of trapeziometacarpal arthroplasty: risk factors, procedures and outcomes. Acta Orthop. 2019;90(4):389-93.
35. Wilkens SC, Xue Z, Mellema JJ, Ring D, Chen N. Unplanned Reoperation After Trapeziometacarpal Arthroplasty: Rate, Reasons, and Risk Factors. Hand (N Y). 2017;12(5):446-52.
36. Partridge TCJ, Baker PN, Jameson SS, Mason J, Reed MR, Deehan DJ. Conventional Versus Highly Cross-Linked Polyethylene in Primary Total Knee Replacement: A Comparison of Revision Rates Using Data from the National Joint Registry for England, Wales, and Northern Ireland. J Bone Joint Surg Am. 2020;102(2):119-27.
37. Penfold CM, Blom AW, Sayers A, Wilkinson JM, Hunt L, Judge A, et al. Understanding the uptake of new hip replacement implants in the UK: a cohort study using data from the National Joint Registry for England and Wales. BMJ Open. 2019;9(11):e029572.
38. BOA. Trauma and Orthopaedic Registries Unifying Structure (TORUS) 2020 [Available from: https://www.boa.ac.uk/policy-engagement/registries-and-outcomes/trauma-orthopaedic-registries-unifying-structure.html [last accessed 24.06.2020].
39. BSSH. UK Hand Registry 2018 [Available from: https://secure.amplitude-registry.com/BSSH/Account/Login?ReturnUrl=%2fBSSH.
40. Parker S, Riley N, Dean B. Management of osteoarthritis at the base of the thumb. Bone Joint J. 2020;102-B(5):600-5.

For peer review only

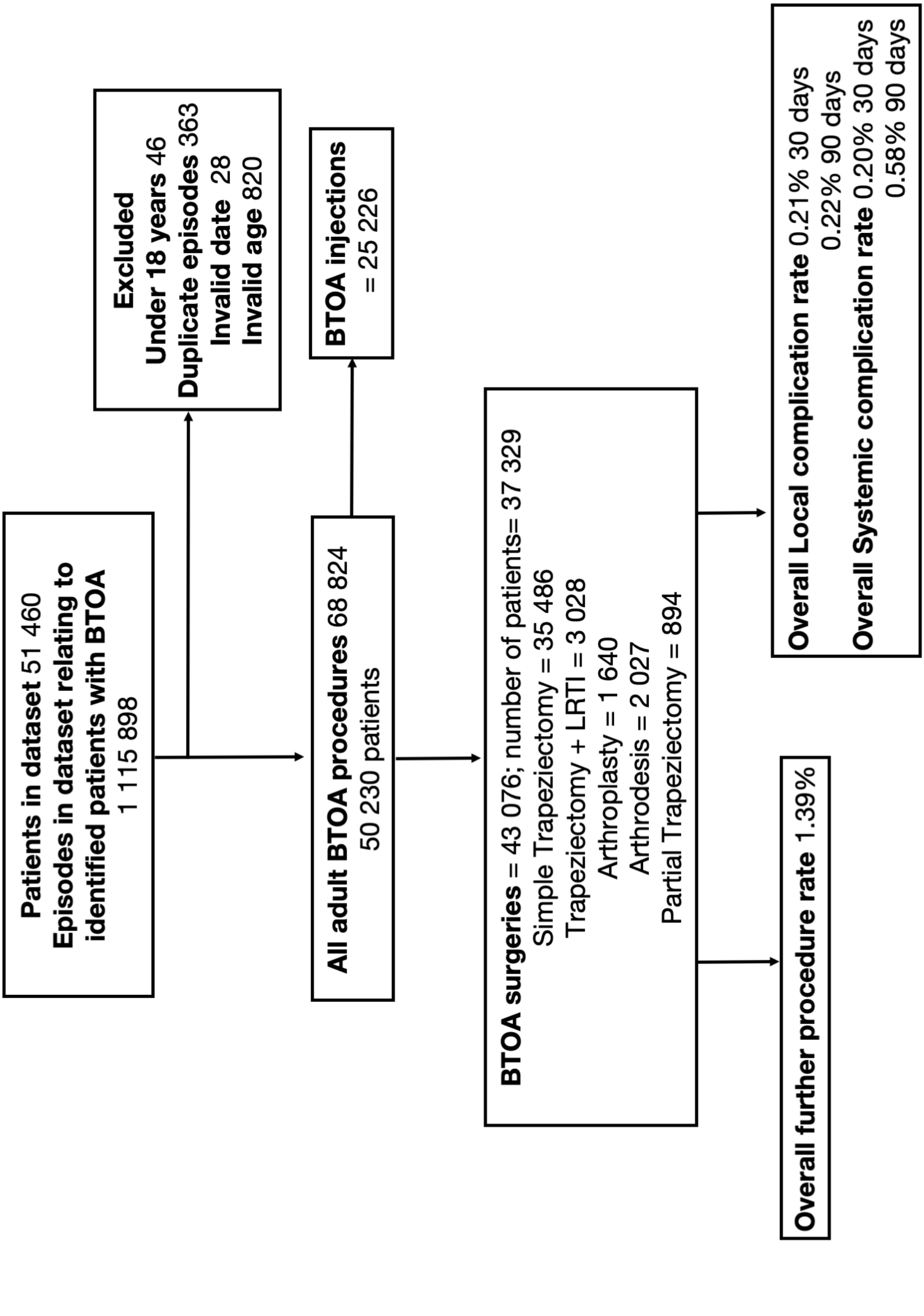
Patients in dataset 51 460
Episodes in dataset relating to identified patients with BTOA
1 115 898
Excluded
Under 18 years 46
Duplicate episodes 363
Invalid date 28
Invalid age 820
BTOA injections
= 25 226
All adult BTOA procedures 68 824
50 230 patients
BTOA surgeries = 43 076; number of patients= 37 329
Simple Trapeziectomy = 35 486
Trapeziectomy + LRTI = 3 028
Arthroplasty = 1 640
Arthrodesis = 2 027
Partial Trapeziectomy = 894
Overall Local complication rate 0.21% 30 days
0.22% 90 days
Overall Systemic complication rate 0.20% 30 days
0.58% 90 days
Overall further procedure rate 1.39%

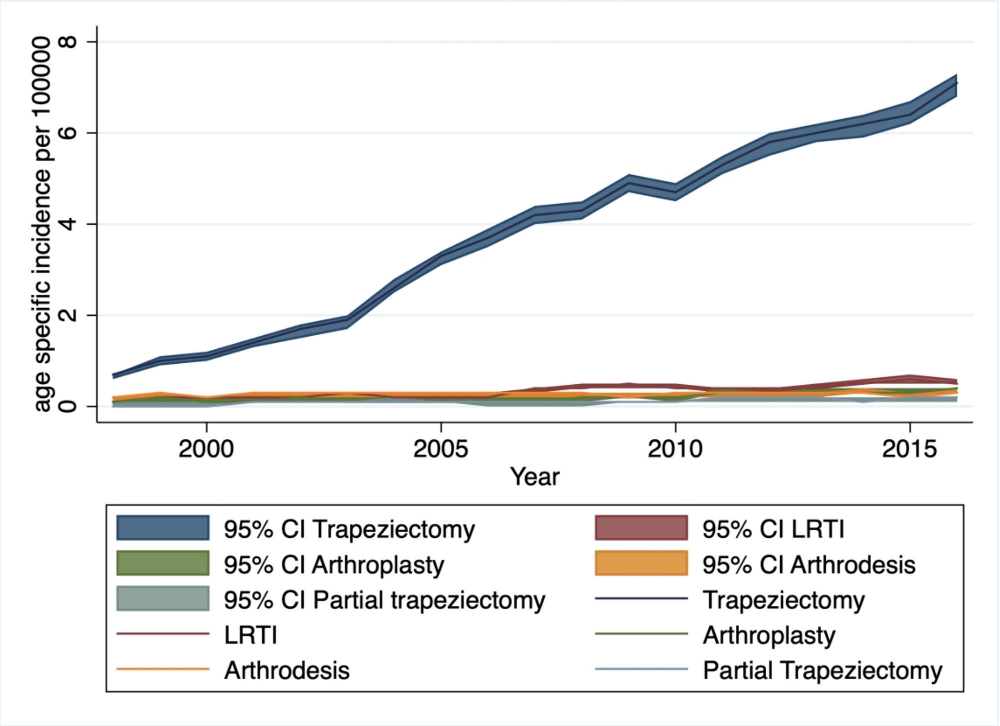

395x287mm (144 x 144 DPI)

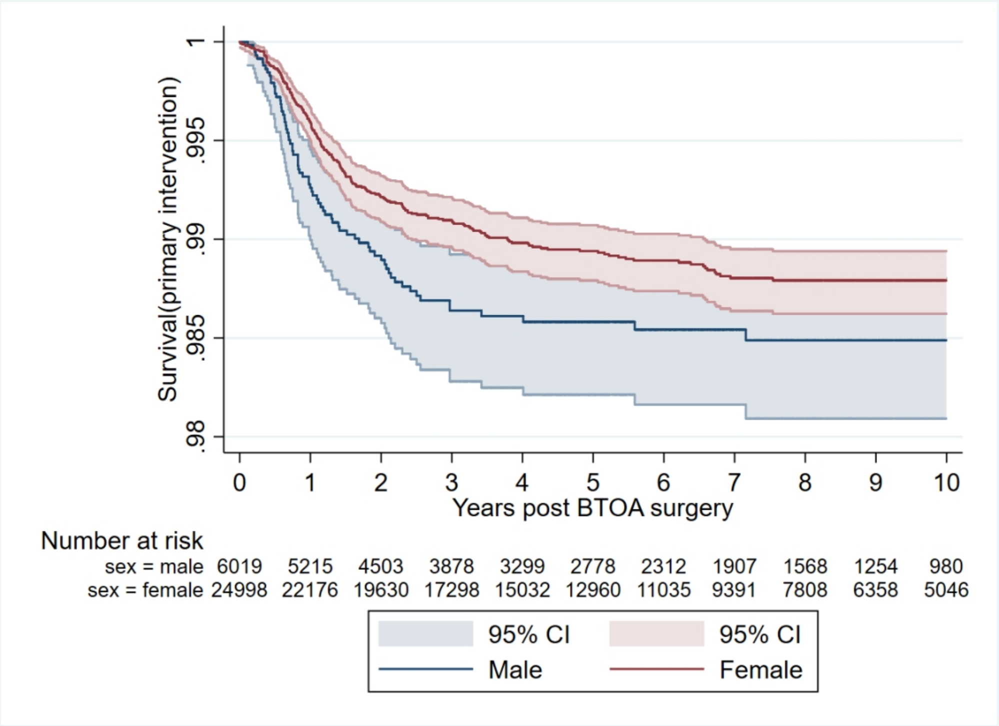

450x327mm (72 x 72 DPI)

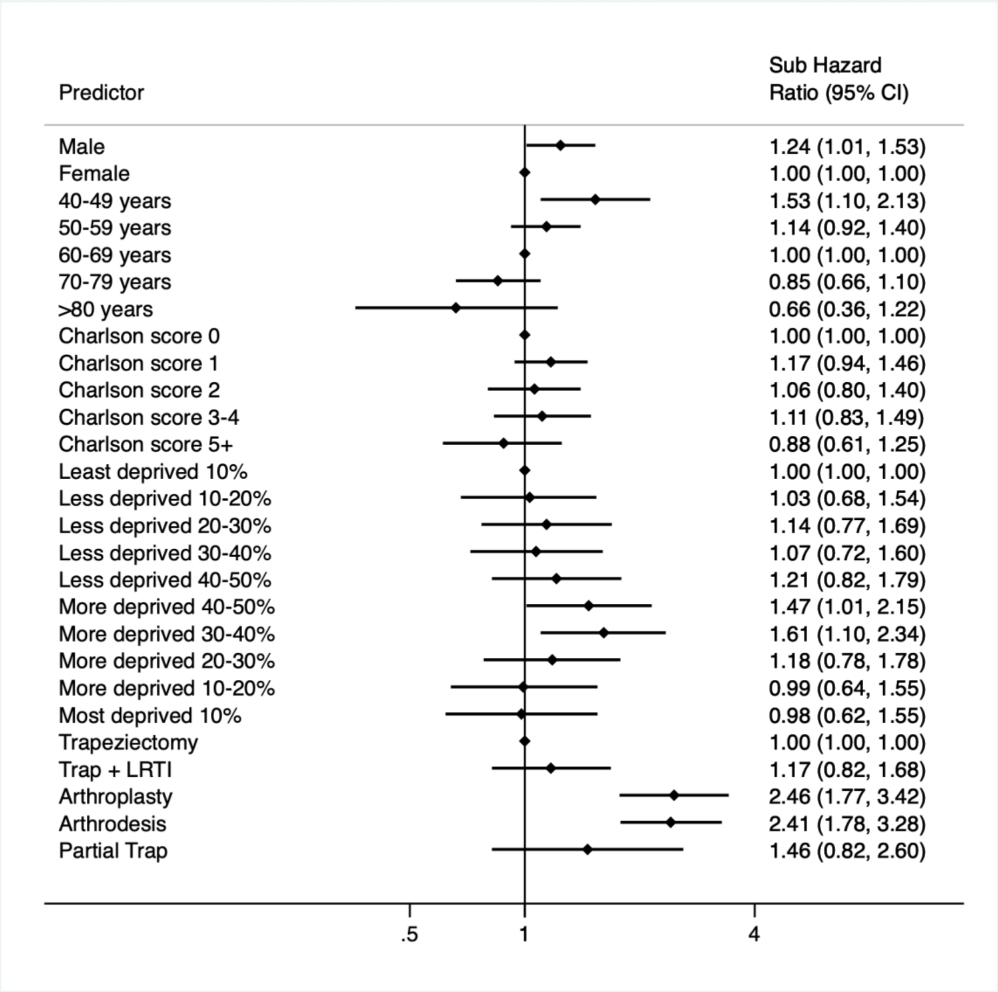

289x287mm (144 x 144 DPI)

Supplementary appendix Low rate of serious complications and further procedures following with BTOA surgery: analysis of national cohort of 43 076 surgeries with up to 19 years of follow up

Supplementary table 1 OPCS and ICD codes used to identify BTOA surgery

**BTOA OPCS codes**		
W57.2	alone	
W57.3	alone	
W57.8	**WITH ANY**	Z82.3
W02.8		Z72.4
W02.9		Z73.8
W06.8		Z73.9
W06.9		Z89.5
W08.5		Z82.2
W08.8		Z82.9
W08.9		
W13.8		
W17.8		
W17.9		
W28.1		
W33.8		
W33.9		
W43.1		
W44.1		
W45.1		
W45.3		
W54.1		
W54.3		
W56.2		
W57.2		
W62.1		
W62.2		
W62.8		
W62.9		
W63.1		
W63.2		

**BTOA ICD-10 codes**
M18.0-18.9
M15.1-2

For peer review only

W63.8		
W63.9		
W64.1		
W64.2		
W64.8		
W64.9		
W74.2		
W74.3		
W77.5		
W77.7		
W77.8		
W90.3		
W90.4		

Supplementary Table 2 Covariate codes

**Covariates for BTOA**	**ICD-10 code**
CTS	G560 (or OPCS A651)
Knee OA	M17
Generalised OA	M15; M19
Rheumatoid Arthritis	M05
Wrist/Hand fracture	S62
Oophorectomy	(OPCS Q22,Q23, Q43)

Supplementary Table 3 Complication codes for systemic complications and local complications

**Complications**	**code**
Wound dehiscence	OPCS Z82 + (S60.4 or S42.2 or S42.3 or S42.4)
Tendon repair	OPCS Z82 + T67
Wound debridement	OPCS Z82 + (T96.8 or T96.3 or T96.4)
Neurovascular injury	ICD code T81.2
Stroke	ICD I60X;I610-I619; I630-I639; I64X
Respiratory Tract Infection	ICD J12X J13, J14, J15X J180-J182 J188; J189; J22; J440 J441 J690 J691 J698 J851
Myocardial Infarction	ICD I210-I214; I219
Deep Vein Thrombosis/ Pulmonary Embolus	ICD I801-I803; I260; I269
Urinary tract infection/ acute renal failure	ICD N300 N390 N170-N172; N178 N179

For peer review only

Supplementary Table 4 Comparison of patients with and without laterality (baseline demographics)

	**With laterality**	**Without laterality**
**Total**	36 072	7004
**Sex**		
Male	7592 (21.05)	1365 (19.49)
Female	28475 (78.94)	5637 (80.48)
Missing	5 (0.01)	2 (0.03)
**Mean age (SD; years)**	63.04 (9.15)	63.13 (9.26)
**Charlson index**		
0	15365 (42.60)	2914 (41.60)
1	8937 (24.78)	1703 (24.31)
2	4746 (13.16)	947 (13.52)
3	2730 (7.57)	556 (7.94)
4	1372 (3.80)	287 (4.10)
>=5	760 (8.10)	166 (8.52)
missing	0	0
**IMD decile**		
Least deprived 10%	4103 (11.37)	646 (9.22)
Less deprived 10-20%	4041 (11.20)	728 (10.39)
Less deprived 20-30%	4055 (11.24)	764 (10.91)
Less deprived 30-40%	4023 (11.15)	795 (11.35)
Less deprived 40-50%	3947 (10.94)	735 (10.49)
More deprived 10-20%	2681 (7.43)	598 (8.54)
More deprived 20-30%	3125 (8.66)	673 (9.61)
More deprived 30-40%	3487 (9.67)	719 (10.27)
More deprived 40-50%	3820 (10.59)	739 (10.55)
Most deprived 10%	2464 (6.83)	575 (8.21)
Missing	326 (0.90)	32 (0.46)
**Ethnic group**		
Any white background	31107 (86.24)	6097 (87)
Any Asian background	380 (1.05)	68 (1)
Any Black background	71 (0.2)	12 (<1)
Any mixed background	51 (0.14)	9 (<1)
Chinese	19 (0.05)	<7*
Any other ethnic group	127 (0.35)	28 (<1)
Not stated	3639 (10.08)	650 (9)
Not known	678 (1.88)	139 (2)
missing	0	0
**Co variates**		
Carpal Tunnel Syndrome	3989 (11.06)	794 (11.34)
Knee Osteoarthritis	4559 (12.64)	936 (13.36)
General Osteoarthritis	7569 (20.98)	1667 (23.8)
Rheumatoid Arthritis	84 (0.23)	25 (0.36)
Wrist Fracture	206 (0.57)	43 (0.61)
Oophorectomy	1351 (3.74)	232 (3.31)

*Numbers less than 7 suppressed in line with NHS Digital disclosure control guidelines- percentages of other groups rounded to prevent secondary disclosure of data (29)

For peer review only

Supplementary table 5 Baseline demographics of all patients undergoing BTOA surgery

	**All surgery (N, %) (total=43 076)**	**Trapeziectomy N (%) (35 486)**	**LRTI N (%) (3028)**	**Arthroplasty N (%) (1640)**	**Arthrodesis N (%) (2027)**	**Partial Trapeziectomy N (%) (894)**
**Sex**						
Male	8957 (20.7)	6 826 (19.2)	616 (20.3)	390 (23.8)	934 (46.1)	191 (21.3)
Female	34 112 (79.1)	28 655 (80.8)	2411 (79.6)	1250 (76.2)	1092 (53.9)	704 (78.7)
Missing	7 (0.02)	5 (0.01)	1 (0.03)	0	1 (0.05)	0
**Mean age (SD; years)**	63.2 (SD 9.2)	63.6 (SD 8.8)	62.5 (SD 9.7)	61.7 (SD 9.3)	58.1 (11.7)	63.1 (9.8)
**Charlson index**						
0	18 279 (42.4)	14 992 (42.3)	1 324 (43.8)	677 (41.3)	890 (43.9)	396 (44.3)
1	10 640 (24.7)	8 807 (24.8)	721 (23.8)	444 (27.1)	475 (23.4)	193 (21.6)
2	5 693 (13.2)	4 746 (13.4)	392 (13.0)	199 (12.1)	250 (12.3)	106 (11.8)
3	3 286 (7.6)	2 723 (7.7)	222 (7.3)	125 (7.6)	142 (7.0)	74 (8.3)
4	1 659 (3.9)	1 359 (3.8)	123 (4.1)	60 (3.7)	77 (3.8)	40 (4.5)
>=5	3 519 (8.2)	2 859 (8.0)	246 (8.1)	135 (8.2)	193 (9.6)	86 (8.5)
missing	0	0	0	0	0	
**IMD decile**						
Least deprived 10%	4 749 (11.0)	4 015 (11.3)	268 (8.8)	203 (12.4)	153 (7.6)	110 (12)
Less deprived 10-20%	4 769 (11.1)	3 934 (11.1)	314 (10.4)	232 (14.15)	195 (9.6)	94 (10)
Less deprived 20-30%	4 819 (11.2)	3 976 (11.2)	354 (11. 5)	185 (11.3)	212 (10.5)	92 (10)
Less deprived 30-40%	4 818 (11.2)	3 974 (11.2)	393 (13.0)	157 (9.6)	212 (10.5)	82 (9)
Less deprived 40-50%	4 682 (10.9)	3 853 (10.9)	345 (11.4)	154 (9.4)	221 (10.9)	109 (12)
More deprived 10-20%	3 279 (7.61)	2 700 (7.61)	218 (7.2)	110 (6.7)	181 (8.9)	70 (7)
More deprived 20-30%	3 798 (8.8)	3 108 (8.8)	273 (9.0)	145 (8.8)	202 (10.0)	70 (7)
More deprived 30-40%	4 206 (9.8)	3 442 (9.7)	303 (10.1)	164 (10.0)	215 (10.6)	82 (9)
More deprived 40-50%	4 559 (10.6)	3 700 (10.4)	354 (11.7)	172 (10.5)	241 (11.9)	92 (10)
Most deprived 10%	3039 (7.1)	2 529 (7.1)	160 (5.3)	110 (6.7)	152 (7.5)	88 (9)
Missing	358 (0.8)	255 (0.7)	46 (1.5)	8 (0.5)	43 (2.1)	<7*
**Ethnic group**						
Any white background	37 204 (86.4)	30 738 (86.6)	2628 (86.8)	1410 (86.0)	1640 (80.9)	755 (84.4)
Any Asian background	439 (1.0)	376 (1.1)	24 (0.8)	20 (1.2)	16 (0.8)	12 (1.3)
Any Black background	92 (0.2)	60 (0.2)	<7*	<7*	12 (0.6)	<7*
Any mixed background	60 (0.1)	46 (0.1)	<7*	<7*	<7*	<7*
Chinese	20 (0.1)	15 (0.05)	<7*	<7*	<7*	<7*
Any other ethnic group	155 (0.4)	130 (0.36)	9 (0.3)	<7*	<7*	<7*
Not stated	1237 (2.9)	3 467 (9.8)	301 (10.0)	134 (8.2)	299 (14.8)	88 (9.8)
Not known	3 869 (9.0)	654 (1.8)	58 (1.9)	26 (1.6)	51 (2.5)	28 (3.1)
Missing	0	0	0	0	0	0
	43 076	35 486	3028	1640	2027	895
**Co variates**						
Carpal Tunnel Syndrome	4 783	3 998 (11.2)	337 (10.9)	178 (10.9)	178 (8.8)	92 (10.3)
Knee Osteoarthritis	5 495	4 643 (13.0)	368 (11.9)	176 (10.7)	205 (10.1)	103 (11.5)
General Osteoarthritis	9 236	7 663 (21.4)	641 (20.8)	378 (23.0)	382 (18.8)	172 (19.2)
Rheumatoid Arthritis	109	81 (0.2)	7 (0.2)	7 (0.4)	12 (0.6)	2 (0.2)
Wrist Fracture	249	183 (0.5)	17 (0.6)	3 (0.2)	39 (1.9)	7 (0.8)
Oophorectomy	1 583	1 371 (3.8)	83 (2.7)	53 (3.2)	50 (2.5)	26 (2.9)

*Numbers less than 7 suppressed in line with NHS Digital disclosure control guidelines- percentages of other groups rounded to prevent secondary disclosure of data (29)

Supplementary Table 6 Local and systemic complications per surgery subtype

	Time	All surgery N (%[95%CI])	Trapeziectomy N (%, 95%CI)	LRTI N (%, 95%CI)	Arthroplasty N (%, 95%CI)	Arthrodesis N (%, 95%CI)	Partial Trapeziectomy N (%, 95%CI)
**Local complications**							
Wound dehiscence and wound infection	Within 30 days	12 (0.03%, [0.01-0.05])	10 (0.03% [0.01-0.05])	<7*	0	<7*	0
	Within 90 days	14 (0.03% [0.02-0.05])	12 (0.03%[0.02-0.06])	<7*	0	<7*	0
Neurovascular injury	Within 30 days	79 (0.18% [0.15-0.23])	68 (0.19%[0.15-0.24])	<7*	0	<7*	<7*
Any complication	Within 30 days	91 (0.21% [0.17-0.26])	78 (0.22%[0.17-0.27])	<7*	0	<7*	<7*
	Within 90 days	93 (0.22% [0.17-0.26])	80 (0.23%[0.18-0.28])	<7*	0	<7*	<7*
**Systemic complications**							
Stroke	Within 30 days	9 (0.02% [0.01-0.04])	9 (0.03% [0.01-0.05])	0	0	0	0
	Within 90 days	40 (0.09% [0.07-0.13])	34 (0.10%[0.07-0.13])	<7*	<7*	<7*	<7*
Respiratory Tract infection	Within 30 days	70 (0.16% [0.13-0.21])	67 (0.19%[0.15-0.24])	<7*	0	0	<7*
	Within 90 days	174 (0.40% [0.35-0.47])	144 (0.41%[0.34-0.48])	10 (0.33%[0.16-0.61])	8 (0.49%[0.21-0.96])	<7*	<7*
Myocardial infarction	Within 30 days	17 (0.04% [0.02-0.06])	15 (0.04% [0.02-0.07])	<7*	0	0	0
	Within 90 days	50 (0.11% [0.09-0.15])	40 (0.11% [0.08-0.15])	<7*	0	<7*	<7*
DVT/PE	Within 30 days	18 (0.04% [0.02-0.07])	9 (0.03% [0.01-0.05])	<7*	<7*	0	0
	Within 90 days	41 (0.10% [0.07-0.13])	30 (0.08%[0.06-0.12])	<7*	<7*	0	<7*
UTI	Within 30 days	30 (0.07% [0.05-0.10])	25 (0.07%[0.05-0.10])	<7*	0	<7*	0
	Within 90 days	75 (0.17%[0.14-0.22])	62 (0.17% [0.13-0.22])	9 (0.30%[0.14-0.56])	0	<7*	<7*
Acute Renal Failure	Within 30 days	13 (0.03%[0.02-0.05])	9 (0.03%[0.01-0.05])	<7*	0	<7*	0
	Within 90 days	44 (0.10%[0.07-0.14])	40 (0.11 [0.08-0.15])	<7*	0	<7*	0
Any Systemic Complication	Within 30 days	87 (0.20%[0.16-0.25])	67 (0.19% [0.15-0.24])	10 (0.33%[0.16-0.61])	<7*	<7*	0
	Within 90 days	250 (0.58% [0.51-0.66])	206 (0.58% [0.50-0.67])	24 (0.79%[0.51-0.12])	<7*	<7*	<7*
Any Systemic Complication (excl UTI & ARF)	Within 30 days	44 (0.10% [0.07-0.14])	33 (0.10% [0.06-0.13])	<7*	<7*	0	0
	Within 90 days	131 (0.30%[0.25-0.36])	104 (0.29% [0.24-0.36])	13 (0.43%[0.23-0.73])	<7*	<7*	<7*

DVT/PE deep vein thrombosis or pulmonary embolic disease
UTI urinary tract infection
ARF Acute Renal Failure
<7 Numbers less than 7 suppressed in line with NHS Digital disclosure control guidelines (29)

For peer review only

Supplementary Table 7 Risk of revision following any BTOA surgery Multivariable regression analysis accounting for competing risk of mortality (without surgical subtype as a risk in the model)

ALL SURGERIES	**Crude sHR[a]**	**95% CI**	**Adj sHR[a]**	**95% CI**
**Male**	1.22	0.99 to 1.49	1.24	1.01 to 1.52
**Age**				
40-49 years	1.53	1.11 to 2.12	1.53	1.10 to 2.13
50-59 years	1.12	0.91 to 1.38	1.14	0.92 to 1.40
60-69 years	1 (ref)	1 (ref)	1 (ref)	1 (ref)
70-79 years	0.86	0.66 to 1.10	0.85	0.66 to 1.10
>80 years	0.66	0.36 to 1.21	0.66	0.36 to 1.22
**Charlson comorbidity index**				
0	1 (ref)	1 (ref)	1 (ref)	1 (ref)
1	1.14	0.92 to 1.42	1.17	0.94 to 1.46
2	0.99	0.75 to 1.31	1.06	0.80 to 1.40
3+	0.92	0.73 to 1.17	1.01	0.78 to 1.30
**IMD**				
Least deprived 10%	1 (ref)	1 (ref)	1 (ref)	1 (ref)
Less deprived 10-20%	1.03	0.69 to 1.54	1.03	0.68 to 1.54
Less deprived 20-30%	1.16	0.78 to 1.72	1.14	0.77 to 1.70
Less deprived 30-40%	1.08	0.73 to 1.61	1.07	0.72 to 1.60
Less deprived 40-50%	1.24	0.84 to 1.83	1.21	0.82 to 1.79
More deprived 40-50%	1.04	0.66 to 1.62	0.99	0.63 to 1.55
More deprived 30-40%	1.24	0.82 to 1.86	1.18	0.79 to 1.78
More deprived 20-30%	1.66	1.14 to 2.42	1.61	1.10 to 2.34
More deprived 10-20%	1.51	1.04 to 2.20	1.47	1.01 to 2.15
Most deprived 10%	1.03	0.65 to 1.63	0.98	0.62 to 1.55
[a] Subhazard ratio				

For peer review only

Supplementary Table 8 Risk of revision following any BTOA surgery Multivariable regression analysis accounting for competing risk of mortality, including surgical subtype as a factor

ALL SURGERIES, including SURG SUBTYPE	**Crude sHR[a]**	**95% CI**	**Adj sHR[a]**	**95% CI**
**Male**	1.22	0.99 to 1.49	1.11	0.89 to 1.38
**Age**				
40-49 years	1.53	1.11 to 2.12	1.24	0.95 to 1.87
50-59 years	1.12	0.91 to 1.38	1.09	0.88 to 1.34
60-69 years	1 (ref)	1 (ref)	1 (ref)	1 (ref)
70-79 years	0.86	0.66 to 1.10	0.86	0.67 to 1.11
>80 years	0.66	0.36 to 1.21	0.67	0.37 to 1.24
**Charlson comorbidity index**				
0	1 (ref)	1 (ref)	1 (ref)	1 (ref)
1	1.14	0.92 to 1.42	1.17	0.94 to 1.46
2	0.99	0.75 to 1.31	1.05	0.79 to 1.40
3+	0.92	0.73 to 1.17	0.99	0.77 to 1.28
**IMD**				
Least deprived 10%	1 (ref)	1 (ref)	1 (ref)	1 (ref)
Less deprived 10-20%	1.03	0.69 to 1.54	1.00	0.67 to 1.50
Less deprived 20-30%	1.16	0.78 to 1.72	1.13	0.76 to 1.67
Less deprived 30-40%	1.08	0.73 to 1.61	1.06	0.71 to 1.58
Less deprived 40-50%	1.24	0.84 to 1.83	1.19	0.81 to 1.77
More deprived 40-50%	1.04	0.66 to 1.62	0.98	0.63 to 1.53
More deprived 30-40%	1.24	0.82 to 1.86	1.16	0.77 to 1.75
More deprived 20-30%	1.66	1.14 to 2.42	1.58	1.09 to 2.29
More deprived 10-20%	1.51	1.04 to 2.20	1.45	1.00 to 2.11
Most deprived 10%	1.03	0.65 to 1.63	0.96	0.61 to 1.52
**Surgery subtype**				
Simple trapeziectomy	1 (ref)	1 (ref)	1 (ref)	1 (ref)
Trapeziectomy + LRTI	1.22	0.86 to 1.73	1.16	0.82 to 1.66
Arthroplasty	2.51	1.81 to 3.48	2.49	1.80 to 3.44
Arthrodesis	2.55	1.91 to 3.40	2.40	1.78 to 3.26
Partial Trapeziectomy	1.44	0.81 to 2.55	1.46	0.82 to 2.59
[a] Subhazard ratio				

For peer review only

Supplementary Table 9 Risk of revision following Trapeziectomy Multivariable regression analysis accounting for competing risk of mortality

TRAPEZIECTOMY	**Crude sHR[a]**	**95% CI**	**Adj sHR[a]**	**95% CI**
**Male**	1.31	1.01 to 1.70	1.32	1.02 to 1.72
**Age**				
40-49 years	1.21	0.76 to 1.93	1.20	0.74 to 1.92
50-59 years	0.96	0.74 to 1.25	0.97	0.73 to 1.27
60-69 years	1 (ref)	1 (ref)	1 (ref)	1 (ref)
70-79 years	0.88	0.65 to 1.19	0.92	0.67 to 1.24
>80 years	0.53	0.23 to 1.19	0.55	0.24 to 1.25
**Charlson comorbidity index**				
0	1 (ref)	1 (ref)	1 (ref)	1 (ref)
1	0.96	0.73 to 1.26	0.97	0.73 to 1.28
2	0.80	0.56 to 1.15	0.79	0.54 to 1.15
3+	0.85	0.63 to 1.14	0.86	0.63 to 1.19
**IMD**				
Least deprived 10%	1 (ref)	1 (ref)	1 (ref)	1 (ref)
Less deprived 10-20	0.86	0.51 to 1.43	0.86	0.51 to 1.43
Less deprived 20-30%	1.03	0.63 to 1.68	1.02	0.63 to 1.67
Less deprived 30-40%	0.97	0.59 to 1.59	0.97	0.59 to 1.60
Less deprived 40-50%	1.13	0.70 to 1.82	1.12	0.69 to 1.81
More deprived 40-50%	0.89	0.50 to 1.57	0.89	0.50 to 1.57
More deprived 30-40%	1.01	0.60 to 1.70	1.00	0.59 to 1.69
More deprived 20-30%	1.68	1.07 to 2.64	1.68	1.07 to 2.63
More deprived 10-20%	1.59	1.01 to 2.49	1.59	1.01 to 2.49
Most deprived 10%	1.06	0.61 to 1.85	1.07	0.62 to 1.85

[a] Subhazard ratio

For peer review only

Supplementary Table 10. Risk of revision following Trapeziectomy with LRTI
Multivariable regression analysis accounting for competing risk of mortality

LRTI	**Crude sHR**	**95% CI**	**Adjusted sHR[a]**	**95% CI**
**Male**	1.26	0.60 to 2.67	1.29	0.60 to 2.76
**Age**				
40-49 years	3.10	1.32 to 7.33	2.64	1.02 to 6.86
50-59 years	0.85	0.39 to 1.88	0.79	0.36 to 1.74
60-69 years	1 (ref)	1 (ref)	1 (ref)	1 (ref)
70-79 years	0.77	0.28 to 2.10	0.68	0.23 to 2.01
>80 years	0.77	0.10 to 5.83	0.81	0.10 to 6.28
**Charlson comorbidity index**				
0	1 (ref)	1 (ref)	1 (ref)	1 (ref)
1	1.14	0.55 to 2.34	1.04	0.50 to 2.13
2	0.79	0.29 to 2.09	0.85	0.31 to 2.31
3+	0.29	0.08 to 0.99	0.30	0.09 to 1.02
**IMD**				
Least deprived 10%	1 (ref)	1 (ref)	1 (ref)	1 (ref)
Less deprived 10-20%	0.40	0.04 to 4.39	0.39	0.04 to 4.26
Less deprived 20-30%	1.77	0.35 to 9.08	1.76	0.35 to 8.85
Less deprived 30-40%	1.39	0.25 to 7.64	1.34	0.24 to 7.33
Less deprived 40-50%	2.63	0.55 to 12.66	2.84	0.59 to 13.55
More deprived 40-50%	2.88	0.56 to 14.92	2.75	0.55 to 13.75
More deprived 30-40%	2.17	0.42 to 11.14	1.90	0.37 to 9.68
More deprived 20-30%	0.85	0.12 to 6.02	0.83	0.12 to 5.89
More deprived 10-20%	1.81	0.35 to 9.35	1.72	0.34 to 8.66
Most deprived 10%	1.52	0.22 to 10.73	1.22	0.17 to 8.92
[a] Subhazard ratio				

For peer review only

Supplementary Table 11. Risk of revision following BTOA Arthroplasty Multivariable regression analysis accounting for competing risk of mortality

Arthroplasty	**Crude sHR**	**95% CI**	**Adj sHR[a]**	**95% CI**
**Male**	0.66	0.31 to 1.40	0.72	0.33 to 1.60
**Age**				
40-49 years	0.92	0.32 to 2.69	1.07	0.37 to 3.10
50-59 years	0.75	0.37 to 1.48	0.80	0.40 to 1.59
60-69 years	1 (ref)	1 (ref)	1 (ref)	1 (ref)
70-79 years	0.83	0.36 to 1.89	0.76	0.31 to 1.84
>80 years	1.63	0.37 to 7.13	1.41	0.28 to 7.25
**Charlson comorbidity index**				
0	1 (ref)	1 (ref)	1 (ref)	1 (ref)
1	1.84	0.83 to 4.11	1.84	0.79 to 4.30
2	2.73	1.16 to 6.42	2.63	1.08 to 6.40
3+	2.47	1.12 to 5.43	2.14	0.88 to 5.24
**IMD**				
Least deprived 10%	1 (ref)	1 (ref)	1 (ref)	1 (ref)
Less deprived 10-20	1.77	0.45 to 6.89	1.71	0.44 to 6.62
Less deprived 20-30%	1.61	0.38 to 6.78	1.44	0.34 to 6.18
Less deprived 30-40%	3.23	0.87 to 12.00	2.96	0.78 to 11.22
Less deprived 40-50%	1.98	0.48 to 8.24	1.47	0.33 to 6.53
More deprived 40-50%	3.02	0.76 to 12.02	2.72	0.68 to 10.91
More deprived 30-40%	1.58	0.36 to 7.04	1.35	0.30 to 6.02
More deprived 20-30%	1.37	0.31 to 6.06	1.31	0.28 to 6.18
More deprived 10-20%	0.65	0.11 to 3.88	0.60	0.10 to 3.59
Most deprived 10%	1.91	0.42 to 8.65	1.22	0.24 to 6.30

[a] Subhazard ratio

For peer review only

Supplementary Table 12. Risk of revision following BTOA Arthrodesis Multivariable regression analysis accounting for competing risk of mortality

Arthrodesis	**Crude sHR**	**95% CI**	**Adj sHR[a]**	**95% CI**
**Male**	0.71	0.42 to 1.21	0.72	0.42 to 1.25
**Age**				
40-49 years	0.74	0.33 to 1.64	0.70	0.31 to 1.54
50-59 years	1 (ref)	1 (ref)	1 (ref)	1 (ref)
60-69 years	0.71	0.38 to 1.31	0.71	0.38 to 1.34
70-79 years	0.72	0.31 to 1.67	0.81	0.32 to 2.05
>80 years	0.51	0.07 to 3.72	0.54	0.08 to 3.86
**Charlson comorbidity index**				
0	1 (ref)	1 (ref)	1 (ref)	1 (ref)
1	2.07	1.10 to 3.88	2.18	1.16 to 4.10
2	1.36	0.60 to 3.12	1.48	0.62 to 3.55
3+	1.04	0.47 to 2.15	1.05	0.46 to 2.41
**IMD**				
Least deprived 10%	1 (ref)	1 (ref)	1 (ref)	1 (ref)
Less deprived 10-20	5.18	0.64 to 42.24	5.42	0.66 to 44.82
Less deprived 20-30%	4.06	0.49 to 33.89	4.16	0.49 to 35.07
Less deprived 30-40%	2.07	0.21 to 20.01	2.03	0.21 to 19.37
Less deprived 40-50%	4.09	0.49 to 34.21	3.93	0.46 to 33.33
More deprived 40-50%	1.69	0.15 to 18.84	1.62	0.14 to 18.29
More deprived 30-40%	6.59	0.83 to 52.31	6.50	0.79 to 53.61
More deprived 20-30%	10.00	1.30 to 76.46	9.77	1.26 to 75.72
More deprived 10-20%	6.05	0.76 to 48.20	5.92	0.72 to 49.01
Most deprived 10%	1.00	0.06 to 16.29	0.94	0.06 to 15.41

[a] Subhazard ratio

For peer review only

Supplementary Figures 1-5 Kaplan Meier plots of risk of further procedure following

1 Simple Trapeziectomy

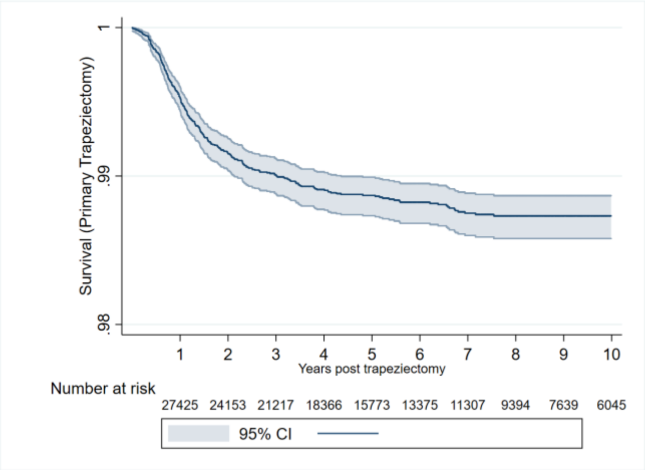

2 Trapeziectomy with LRTI

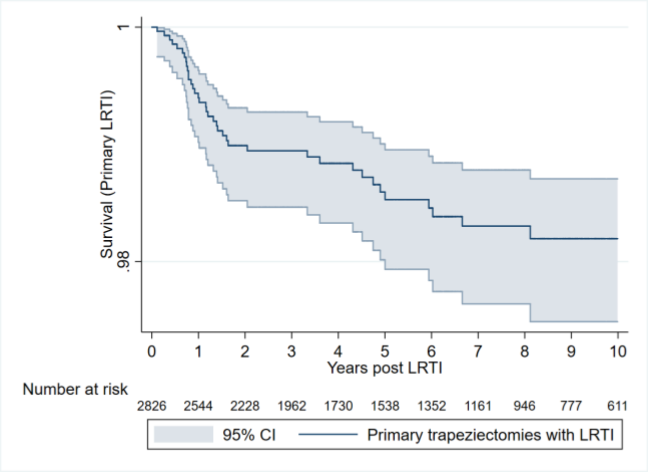

3 BTOA Arthroplasty

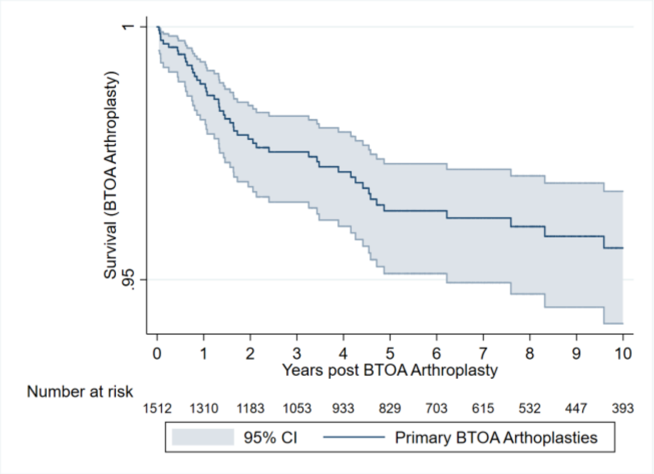

4 BTOA Arthrodesis

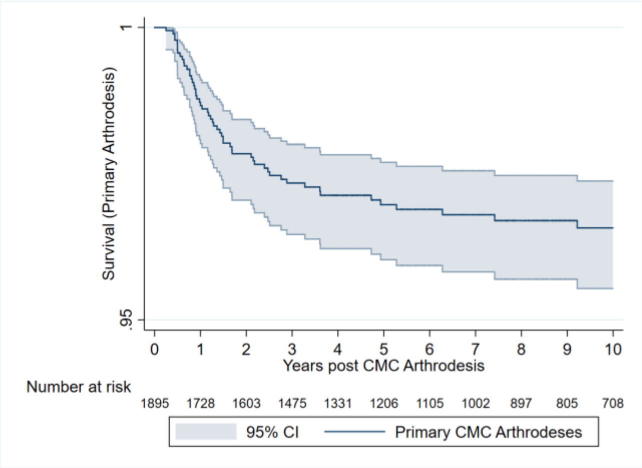

5 Partial Trapeziectomy

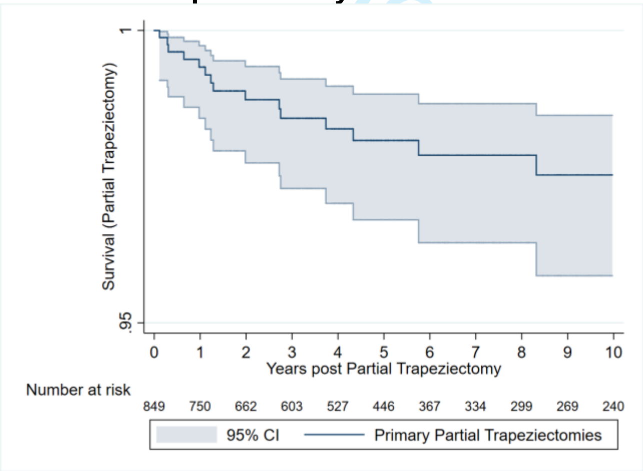

STROBE Statement—Checklist of items that should be included in reports of ***cohort studies***

	Item No	Recommendation	Page No
**Title and abstract**	1	(*a*) Indicate the study's design with a commonly used term in the title or the abstract<br>(*b*) Provide in the abstract an informative and balanced summary of what was done and what was found	1-2
**Introduction**			
Background/rationale	2	Explain the scientific background and rationale for the investigation being reported	4
Objectives	3	State specific objectives, including any prespecified hypotheses	4
**Methods**			
Study design	4	Present key elements of study design early in the paper	4-5
Setting	5	Describe the setting, locations, and relevant dates, including periods of recruitment, exposure, follow-up, and data collection	*4*
Participants	6	(*a*) Give the eligibility criteria, and the sources and methods of selection of participants. Describe methods of follow-up<br>(*b*) For matched studies, give matching criteria and number of exposed and unexposed	4-5
Variables	7	Clearly define all outcomes, exposures, predictors, potential confounders, and effect modifiers. Give diagnostic criteria, if applicable	5-6
Data sources/ measurement	8*	For each variable of interest, give sources of data and details of methods of assessment (measurement). Describe comparability of assessment methods if there is more than one group	4 & 5
Bias	9	Describe any efforts to address potential sources of bias	5
Study size	10	Explain how the study size was arrived at	4
Quantitative variables	11	Explain how quantitative variables were handled in the analyses. If applicable, describe which groupings were chosen and why	5-6
Statistical methods	12	(*a*) Describe all statistical methods, including those used to control for confounding<br>(*b*) Describe any methods used to examine subgroups and interactions<br>(*c*) Explain how missing data were addressed<br>(*d*) If applicable, explain how loss to follow-up was addressed<br>(*e*) Describe any sensitivity analyses	5-6
**Results**			
Participants	13*	(a) Report numbers of individuals at each stage of study—eg numbers potentially eligible, examined for eligibility, confirmed eligible, included in the study, completing follow-up, and analysed<br>(b) Give reasons for non-participation at each stage<br>(c) Consider use of a flow diagram	7
Descriptive data	14*	(a) Give characteristics of study participants (eg demographic, clinical, social) and information on exposures and potential confounders<br>(b) Indicate number of participants with missing data for each variable of interest<br>(c) Summarise follow-up time (eg, average and total amount)	7 + supp
Outcome data	15*	Report numbers of outcome events or summary measures over time	8 + supp

For peer review only

For peer review only - http://bmjopen.bmj.com/site/about/guidelines.xhtml

Main results	16	(*a*) Give unadjusted estimates and, if applicable, confounder-adjusted estimates and their precision (eg, 95% confidence interval). Make clear which confounders were adjusted for and why they were included<br>(*b*) Report category boundaries when continuous variables were categorized<br>(*c*) If relevant, consider translating estimates of relative risk into absolute risk for a meaningful time period	7-9
Other analyses	17	Report other analyses done—eg analyses of subgroups and interactions, and sensitivity analyses	supplementary
**Discussion**			
Key results	18	Summarise key results with reference to study objectives	10
Limitations	19	Discuss limitations of the study, taking into account sources of potential bias or imprecision. Discuss both direction and magnitude of any potential bias	11
Interpretation	20	Give a cautious overall interpretation of results considering objectives, limitations, multiplicity of analyses, results from similar studies, and other relevant evidence	10
Generalisability	21	Discuss the generalisability (external validity) of the study results	10
**Other information**			
Funding	22	Give the source of funding and the role of the funders for the present study and, if applicable, for the original study on which the present article is based	12

*Give information separately for exposed and unexposed groups.

Note: An Explanation and Elaboration article discusses each checklist item and gives methodological background and published examples of transparent reporting. The STROBE checklist is best used in conjunction with this article (freely available on the Web sites of PLoS Medicine at http://www.plosmedicine.org/, Annals of Internal Medicine at http://www.annals.org/, and Epidemiology at http://www.epidem.com/). Information on the STROBE Initiative is available at http://www.strobe-statement.org.

For peer review only

Low rates of serious complications and further procedures following surgery for Base of Thumb Osteoarthritis: analysis of a national cohort of 43 076 surgeries

Journal:	*BMJ Open*
Manuscript ID	bmjopen-2020-045614.R2
Article Type:	Original research
Date Submitted by the Author:	18-Mar-2021

Complete List of Authors:	Lane, Jennifer; University of Oxford Nuffield Department of Orthopaedics Rheumatology and Musculoskeletal Sciences<br>Craig, Richard; Oxford University, Nuffield Department of Orthopaedics, Rheumatology and Musculoskeletal Sciences<br>Rees, J L; Oxford University, Nuffield Department of Orthopaedics, Rheumatology and Musculoskeletal Sciences<br>Gardiner, Matthew; University of Oxford Nuffield Department of Orthopaedics Rheumatology and Musculoskeletal Sciences; Wexham Park Hospital, Plastic Surgery<br>Mikhail, Mark; Oxford University Hospitals NHS Foundation Trust Nuffield Orthopaedic Centre, Plastic Surgery<br>Riley, Nicholas; Oxford University Hospitals NHS Foundation Trust Nuffield Orthopaedic Centre, Hand Surgery<br>Prieto-Alhambra, Daniel; Oxford University, Nuffield Department of Orthopaedics, Rheumatology and Musculoskeletal Sciences<br>Furniss, Dominic ; Oxford University, Nuffield Department of Orthopaedics Rheumatology and Musculoskeletal Science; Oxford University Hospitals NHS Foundation Trust Nuffield Orthopaedic Centre, Plastic Surgery

**Primary Subject Heading**:	Surgery
Secondary Subject Heading:	Epidemiology
Keywords:	SURGERY, Epidemiology < TROPICAL MEDICINE, Hand & wrist < ORTHOPAEDIC & TRAUMA SURGERY, Adult orthopaedics < ORTHOPAEDIC & TRAUMA SURGERY, RHEUMATOLOGY

SCHOLARONE™
Manuscripts

I, the Submitting Author has the right to grant and does grant on behalf of all authors of the Work (as defined in the below author licence), an exclusive licence and/or a non-exclusive licence for contributions from authors who are: i) UK Crown employees; ii) where BMJ has agreed a CC-BY licence shall apply, and/or iii) in accordance with the terms applicable for US Federal Government officers or employees acting as part of their official duties; on a worldwide, perpetual, irrevocable, royalty-free basis to BMJ Publishing Group Ltd ("BMJ") its licensees and where the relevant Journal is co-owned by BMJ to the co-owners of the Journal, to publish the Work in this journal and any other BMJ products and to exploit all rights, as set out in our licence.

The Submitting Author accepts and understands that any supply made under these terms is made by BMJ to the Submitting Author unless you are acting as an employee on behalf of your employer or a postgraduate student of an affiliated institution which is paying any applicable article publishing charge ("APC") for Open Access articles. Where the Submitting Author wishes to make the Work available on an Open Access basis (and intends to pay the relevant APC), the terms of reuse of such Open Access shall be governed by a Creative Commons licence – details of these licences and which Creative Commons licence will apply to this Work are set out in our licence referred to above.

Other than as permitted in any relevant BMJ Author's Self Archiving Policies, I confirm this Work has not been accepted for publication elsewhere, is not being considered for publication elsewhere and does not duplicate material already published. I confirm all authors consent to publication of this Work and authorise the granting of this licence.

For peer review only

Low rates of serious complications and further procedures following surgery for Base of Thumb Osteoarthritis: analysis of a national cohort of 43 076 surgeries

Jennifer C.E. Lane MRCS[1], Richard S. Craig DPhil FRCS (Tr&Orth)[1], Jonathan. L. Rees MD, FRCS (Tr&Orth) [1], Matthew D. Gardiner PhD, FRCS(Plast)[2,3], Mark Mikhail MRCS[4], Nicholas D Riley FRCS(Tr&Orth)[5], Daniel Prieto-Alhambra, DPhil[1*], Dominic Furniss, DM FRCS(Plast)[1,4*]

1. Oxford NIHR Musculoskeletal Biomedical Research Unit, Nuffield Department of Orthopaedics, Rheumatology and Musculoskeletal Sciences, Nuffield Orthopaedic Centre, University of Oxford, Windmill Road, Oxford, OX3 7LD, UK
2. Department of Plastic Surgery, Wexham Park Hospital, Slough, SL2 4HL, UK
3. Kennedy Institute of Rheumatology, Nuffield Department of Orthopaedics, Rheumatology and Musculoskeletal Sciences, Roosevelt Drive, Oxford, OX3 7FY, UK
4. Department of Plastic and Reconstructive Surgery, Nuffield Orthopaedic Centre, Windmill Road, Oxford, OX3 7HE, UK
5. Department of Hand Surgery, Nuffield Orthopaedic Centre, Windmill Road, Oxford, OX3 7HE, UK

* authors co-directed this work

Corresponding author: Professor Dominic Furniss, Nuffield Department of Orthopaedics, Rheumatology and Musculoskeletal Sciences, University of Oxford, Windmill Road, Oxford, OX3 7LD, UK dominic.furniss@ndorms.ox.ac.uk

Word Count (excl title page, abstract refs figures and tables): 3967

Keywords: base of thumb osteoarthritis, surgery, adverse event, revision, epidemiology

For peer review only

ABSTRACT

Objectives

To determine the incidence of further procedures and serious adverse events (SAE) requiring admission to hospital following elective surgery for base of thumb osteoarthritis (BTOA), and the patient factors associated with these outcomes.

Design

Population based cohort study

Setting

National Health Service (NHS) using the national Hospital Episode Statistics dataset linked to mortality records over a 19-year period (01/04/1998-31/03/2017).

Participants

43 076 primary surgeries were followed longitudinally in secondary care until death or migration on 37 329 patients over 18 years of age.

Main Outcome measures

Incidence of further thumb base procedures (including revision surgery or intra-articular steroid injection) at any time post-operatively, and local wound complications and systemic events (myocardial infarction, stroke, respiratory tract infection, venous thromboembolic events, urinary tract infection or renal failure) within 30 and 90 days. To identify patient factors associated with outcome, Fine and Gray model regression analysis was used to adjust for the competing risk of mortality in addition to age, overall comorbidity and socioeconomic status.

Results

Over the 19-years, there was an increasing trend in surgeries undertaken. The rate of further thumb base procedures after any surgery was 1.39%; the lowest rates after simple trapeziectomy (1.12%), the highest rates after arthroplasty (3.84%), and arthrodesis (3.5%). When matched for age, comorbidity and socioeconomic status, those undergoing arthroplasty and arthrodesis were 2.5 times more likely to undergo a further procedure (subhazard ratio 2.51 [95%CI 1.81-3.48]) and 2.55 [1.91-3.40]) than those undergoing simple trapeziectomy. Overall complication rates following surgery were 0.22% for serious local complications and 0.58% for systemic events within 90 days of surgery.

Conclusions

The number of patients proceeding to BTOA surgery has increased over the last 19 years, with a low rate of further thumb base procedures and SAEs after surgery overall registered. Arthrodesis and arthroplasty had a significantly higher revision rate.

Trial Registration

Clinicaltrials.gov (NCT03573765)
NHS Digital Data Sharing Agreement (DARS-NIC-29827-Q8Z7Q)

For peer review only - http://bmjopen.bmj.com/site/about/guidelines.xhtml

STRENGTHS AND LIMITATIONS OF THIS STUDY

- To our knowledge, this is the largest scale national study identifying the risks following surgery for base of thumb osteoarthritis
- Long follow up is enabled by a nationalised health system linked to mortality records, that can identify surgical intervention in England over the last 19 years
- Using data from a nationalised health system has the benefit of capturing data from patients who present to another hospital for treatment of their complication
- As this study uses data collected from routine clinical care, results have a greater chance of being generalisable to a whole population
- This dataset can only register complications presenting to hospital, so is limited to identifying serious adverse events requiring day case admission or further surgery.

For peer review only

INTRODUCTION

Background

Osteoarthritis of the first carpometacarpal joint, or base of thumb osteoarthritis (BTOA), is very common and may need surgical treatment.[1] Worldwide, the majority of surgical interventions are simple trapeziectomy with or without ligament reconstruction and tendon interposition (LRTI).[1-3] However, there is increasing interest in the use of arthroplasty for BTOA, with many different implants available.[4-10] Concerns have been raised regarding increased risk of complications with LRTI and arthroplasty compared with simple trapeziectomy.[11, 12] A Cochrane review comparing surgical techniques concluded that there was a high risk of bias within current studies, with no single technique presenting superior results.[13] Recent work aimed to identify complications within a large insurance provider in the US for small joint hand osteoarthritis procedures, and found complication rates as high as 35%, including 5% risk of a complication related to the prosthesis within two years of surgery.[14]

Many surgical studies in BTOA compare two techniques either within a clinical trial or single centre cohort, but there are very few studies comparing all available techniques with a long follow up at a national level. Routinely collected data produced from everyday clinical practice offers the advantage of enabling long-term follow up and detection of rare outcomes for healthcare interventions in the general population. [15] Routinely collected data for hospital admissions within the nationalised health service in England has been widely used to evaluate the safety and outcomes following surgery for other studies musculoskeletal conditions.[16-18]

The primary aim of this study was to estimate the incidence of BTOA surgery, further thumb base procedures and complications requiring admission to hospital in adults in the NHS in England. Secondary aims were to identify factors associated with adverse outcomes, including registered local complications and systemic events, and further thumb base procedures after surgery. Our hypothesis was that there is variation in outcome between surgical subtypes.

METHODS

Data Source

A pseudonymised, bespoke extract was made from the NHS database Hospital Episode Statistics Admitted Patient Care (HES APC) covering the period 1st April 1998 to 31st March 2017).[19] This individual patient level data extract contained all patient episodes of care within the NHS England system, including those episodes that occur within independent providers that are remunerated by the NHS. The NHS provides the majority of healthcare in England, with only 13% of elective surgery estimated to be privately funded.[20] Episodes of care are linked via a patient's individual NHS number, therefore enabling linkage of all treatments undertaken and longitudinal follow up, including complications and revision surgeries undertaken and registered by any provider. The HES APC extract was also linked to the ONS national mortality dataset prior to pseudonymization to identify cause and date of death.[21] By design, raw data was collected by NHS Digital prior to delivery to the research team.

Population

All patients over 18 years undergoing BTOA surgery were identified using a list of OPCS Classification of Interventions and Procedures (OPCS-4.8) to identify the procedures undertaken and International Classification of Diseases (ICD-10) codes to identify disease associated with the procedure(Supplementary tables 1-3).[22, 23]. OPCS codes are used in

For peer review only - http://bmjopen.bmj.com/site/about/guidelines.xhtml

England to classify any procedure undertaken, and are used to identify a combination of the implant and surgical subtype used in association with the anatomical area of the body where the surgery took place. For hand surgery, OPCS codes are used with combination of a generic surgical procedure codes and anatomical location.
All episodes of care for each individual prior to and following BTOA surgery were included in the extract. Patients were followed up until date of death or the end of the study period (31/03/2017). Patients who had an ICD-10 code for traumatic injury in the same episode as BTOA surgery were excluded, in order to only include patients undergoing elective surgery for longstanding BTOA. Duplicate episodes can occur over the change of financial year, and were removed during data cleaning. Each hand was considered as a separate surgical case. All cases of BTOA surgery identified within the 19 year period were included if they were not associated with a fracture ICD diagnosis code, and were not considered to be a duplicate episode. All cases were included with the aim of identifying the full national cohort within the time frame. Minimum follow up was set at one day, due to the clinical opinion of the team that further procedure or serious adverse events can occur very shortly after surgery (for example, acute carpal tunnel syndrome requiring decompression).

Validation: determining the feasibility of identifying surgical procedures and BTOA cases
2 Validation studies were undertaken in order to determine if it was possible to successfully identify surgical treatment of BTOA in this dataset, and secondly to determine if it was possible to identify the surgical subtypes undertaken.

Firstly, we undertook consensus discussion between surgeons, coders and NHS Digital to determine the most likely OPCS procedure codes and ICD diagnosis codes that would identify surgery for BTOA within routinely collected hospital data in England. This was an iterative process within a diverse team of stakeholders. Following this, an external validation study was then undertaken using this list of OPCS and ICD codes that identified a positive predictive value of 81% for incident BTOA with good interobserver reliability.[24]

Secondly, we undertook a further external validation study to determine if OPCS procedure codes could identify surgery subtypes within routinely collected data in the NHS in England. This was undertaken in our NHS trust using 2 blinded and independent reviewers (MM and NDR), who reviewed a year of surgical activity and the OPCS and ICD codes used during this time. In a year's sample of 104 patients undergoing BTOA surgery in our institution, we demonstrated a positive predictive value of 99% in identifying surgical subtype.

Covariates
Socioeconomic status of patients was identified using the Index of Multiple Deprivation (IMD), a government generated score of deprivation upon geographical location in England.[25] Overall comorbidity level was calculated using the Charlson Comorbidity Index (CCI). [26, 27] Ethnicity, as collected by NHS Digital, was included as a baseline covariate of interest, as national data is more inclusive than trial data, allowing us to identify any effect of ethnicity.[28] Baseline incidence of other conditions associated with BTOA pathogenesis in the literature were also identified using OPCS and ICD codes from previous episodes of secondary care, including a past medical history of carpal tunnel syndrome, knee osteoarthritis, generalized osteoarthritis, rheumatoid arthritis, wrist fracture, and oophorectomy.

Further thumb base procedures
Further thumb base procedure was defined as any revision surgery or intra-articular steroid injection in the same thumb base following surgery. In order to identify factors associated with further thumb base procedure, time to further procedure and produce an incidence rate, only further procedures registered with BTOA surgery or injection codes *in the same hand* were used, identified through a laterality code. Also, to produce an estimation of a 'worst

For peer review only - http://bmjopen.bmj.com/site/about/guidelines.xhtml

case' scenario of the percentage of cases that *may* have proceeded to further thumb base procedure, patients who had 3 or more episodes with BTOA surgery or injection codes but missing laterality were included in the percentage, but were not included in all subsequent analyses.

Complications

Complications were grouped into local (surgical site infection or dehiscence requiring surgery, and neurovascular or tendon injury) and systemic events (stroke, respiratory tract infection, acute myocardial infarction, venous thromboembolic disease, urinary tract infection and acute renal failure). All complications had to be registered within HES APC to be identified and therefore required at least day case admission or surgery; minor complications will therefore not be registered in the dataset. Registered complications were defined within 30 and 90 days of surgery to align with NHS outcomes framework used to compare outcomes between different medical conditions.[29] Furthermore, in the NHS, all surgical activity is compared with regards to outcomes within 30 and 90 days, and therefore exploring serious adverse events that occur within this timeframe enables comparison to other musculoskeletal conditions and limb surgeries, and to studies already published in the literature.[16-18] It was also our clinical experience that these serious local and systemic complications requiring hospital admission were likely to present within these time periods. Registered serious systemic events were studied in addition to serious local complications due to concern about the side effects of general anaesthesia associated with upper limb procedures, and to enable comparison to rates registered following surgery for other musculoskeletal conditions.[17, 18]

Statistical methods

Age and sex specific incidence of surgery were calculated using official national mid-year population estimates in order to reflect the national nature of the healthcare system being studied, and to enable comparison to other musculoskeletal conditions, or study results from other countries.[30] Producing age and sex specific incidence also enables the results to take into account the change in population demographics over the long time period studied. All complications were calculated as a proportion of the sample with 95% confidence intervals. Incomplete records were found for 0.95% of cases for age, sex, ethnicity and IMD deciles. Data was assumed to be missing at random, and a complete case analysis undertaken without imputation. Laterality was missing in 16.2% of cases, and in order to assess if missing laterality caused a bias in included cases, the baseline demographics of patients with and without laterality were compared (supplementary table 4). No difference in baseline demographics was seen and therefore this was not considered as a potential cause of bias.

Factors associated with a further thumb base procedure were identified using a multivariable regression analysis using a Fine and Gray model that accounts for the competing risk of mortality.[31] The Fine and Gray model is a proportional hazards model that produces a subhazard ratio, that is derived from the cumulative incidence function (cause specific hazard). The Fine and Gray model splits the risk into two parts, the risk of further procedure, against (in this context) the risk of death. This is important within this study due to the long follow up and potential for patients to die within the follow up period, and allows the model to identify those who undergo a further thumb base procedure only within those who remain alive. Adjustment was undertaken for sex, age, socioeconomic status (using IMD), and overall comorbidity (using CCI). As registered complications were assessed within 90 days of surgery, multivariable cox proportional hazard modelling was used without adjusting for the competing risk of mortality. Patient age was identified as having a non-linear relationship with further thumb base procedure, and therefore was categorized, with the category containing the median age used as the reference category. When dividing the risk of further procedure by surgical subtype, there were insufficient patients below the age of 40 for inclusion in the multivariable regression analysis. Multivariable regression analysis was

For peer review only - http://bmjopen.bmj.com/site/about/guidelines.xhtml

undertaken for all patient requiring further thumb base procedure, and also for those who had trapeziectomy alone, trapeziectomy with LRTI, arthroplasty and arthrodesis. For partial trapeziectomy there were insufficient numbers of patients who had a further thumb base procedure to undertake this analysis.

In order to assess the impact of surgical subtype, the main multivariable regression analysis was undertaken to compare the risk of further thumb base procedure for each surgical subtype compared to simple trapeziectomy. Further sensitivity analyses were undertaken, where each surgical subtype was assessed as an individual cohort in separate multivariable models adjusted for age, sex and socioeconomic status. These were undertaken to investigate each cohort of patients undergoing a particular surgical procedure, to assess for the potential of confounding by indication within the main model where all patients were combined as one group.

Statistical analysis was undertaken using STATA version 15.1. Significance within the multivariable regression analysis was determined for a subhazard ratio (sHR) when a 95% confidence interval did not include 1, giving a more granular picture of the data than a p-value alone. No power calculation was undertaken as this study used all available data within a national cohort analysis.

PATIENT AND PUBLIC INVOLVEMENT

A James Lind Alliance priority setting partnership focused on common conditions affecting the hand and wrist also raised the question *'which interventions give the best results in the treatment of painful joints in the hand and wrist'* as important to patients, making this study a direct response in its research question.[32] No patients were directly involved in the study design, conduct or interpretation of the study.

RESULTS

Data processing

Figure 1 describes the data processing details. In the study period, 43 076 primary BTOA surgeries were performed on 37 329 individuals in English NHS hospitals. 79.1% were performed on female patients, and the mean age at primary surgery was 63 (SD 9.2). The median follow up time was 1835 days (interquartile range 816 to 3223). Only 345 cases (0.8%) had a follow up time of less than 30 days, and 1075 cases (2.5%) had a follow up time of less than 90 days. It was therefore considered that loss to follow up was not a sufficient source of bias within the cohort.

Demographics of patients undergoing BTOA surgery are shown in Table 1, with further granularity given in Supplementary Table 5. Over 60% of patients had no or very low levels of overall comorbidity, and 86% of patients were of white ethnic background. Patients were more likely to come from affluent sections of society. Approximately 1 in 5 patients had another hospital episode prior to BTOA surgery for osteoarthritis elsewhere in the body.

The most commonly undertaken surgical procedure was simple trapeziectomy, followed by trapeziectomy with LRTI. When comparing the baseline demographics of patients undergoing the different surgical subtypes, more men underwent arthrodesis and were slightly younger compared to the other groups. There appeared to be no difference in overall comorbidity as shown by Charlson index, and comparable socioeconomic status of patients shown by the index of multiple deprivation (IMD).

Table 1. Demographics of patients undergoing BTOA surgery

	**All surgery (N, %) (total=43 076)**	**Trapeziectomy N (%) (35 486)**	**LRTI N (%) (3028)**	**Arthroplasty N (%) (1640)**	**Arthrodesis N (%) (2027)**	**Partial Trapeziectomy N (%) (894)**
**Female sex**	34 112 (79.1)	28 655 (80.8)	2411 (79.6)	1250 (76.2)	1092 (53.9)	704 (78.7)

**Mean age (SD; years)**	63.2 (SD 9.2)	63.6 (SD 8.8)	62.5 (SD 9.7)	61.7 (SD 9.3)	58.1 (11.7)	63.1 (9.8)
**Charlson Comorbidity index**						
0	18 279 (42.4)	14 992 (42.3)	1 324 (43.8)	677 (41.3)	890 (43.9)	396 (44.3)
1	10 640 (24.7)	8 807 (24.8)	721 (23.8)	444 (27.1)	475 (23.4)	193 (21.6)
2	5 693 (13.2)	4 746 (13.4)	392 (13.0)	199 (12.1)	250 (12.3)	106 (11.8)
3	3 286 (7.6)	2 723 (7.7)	222 (7.3)	125 (7.6)	142 (7.0)	74 (8.3)
4	1 659 (3.9)	1 359 (3.8)	123 (4.1)	60 (3.7)	77 (3.8)	40 (4.5)
>=5	3 519 (8.2)	2 859 (8.0)	246 (8.1)	135 (8.2)	193 (9.6)	86 (8.5)
**Index of Multiple Deprivation (IMD)**						
Quintile 1 (least deprived)	9 518 (22.1)	7 949 (22.4)	582 (19.2)	435 (26.5)	348 (17.2)	204 (22.8)
Quintile 2	9 637 (22.4)	7 950 (22.4)	747 (24.6)	342 (20.9)	424 (21)	174 (19.5)
Quintile 3	7 961 (18.5)	6553 (18.5)	563 (18.6)	264 (16.1)	402 (19.8)	179 (20)
Quintile 4	8 004 (18.6)	6550 (18.5)	576 (19.1)	309 (18.8)	417 (20.6)	152 (17)
Quintile 5 (Most deprived)	7 598 (17.7)	6229 (17.5)	514 (17.0)	282 (17.2)	393 (19.4)	180 (20.1)
Missing	358 (0.8)	255 (0.7)	46 (1.5)	8 (0.5)	43 (2.0)	6 (0.7)

Trends in surgery

Over the 19-year period there was an increasing trend in the number of BTOA surgeries undertaken, predominantly accounted for by a steady increase in simple trapeziectomy, with minimal change in the incidence of other surgical procedures. (Figure 2)

Further Thumb Base Procedures

The overall rate of further thumb base procedure after any BTOA surgery was 1.39%, with the median time to intervention being under 1.5 years (Table 2; Figure 3). Whilst the rate of further thumb base procedures was low in general in this population, the highest rates were seen after arthroplasty (3.84%) and arthrodesis (3.50%) and the lowest following simple trapeziectomy (1.12%). The survival of BTOA surgery over time according to surgical subtype is given in Kaplan-Meier plots in the appendix (Supplementary Figures 1-5), noting the majority of patients proceeded to further intervention around the first 18 months post-operatively.

Table 2. Rate of further thumb base procedure (revision surgery or intraarticular steroid injection) according to BTOA surgical subtype

	Number of cases	Median follow up time in days (IQR)	Number cases needing further procedures[a] (%)	Median time to further procedure[a] in days (IQR)	Further procedure[a] rate per 1000 person years (95%CI)
All BTOA surgeries	43 076	1835 (816 to 3223)	599 (1.39%)	472 (272 to 965)	1.72 (1.54 to 1.92)
Trapeziectomy	35 486	1839 (820 to 3228)	398 (1.12%)	420.5 (248 to 804)	1.73 (1.55 to 1.93)
Trapeziectomy with LRTI	3 028	2063.5 (880 to 3405)	45 (1.49%)	508 (287 to 1648)	2.13 (1.56 to 2.92)
Arthroplasty	1 640	2000.5 (865 to 3683.5)	63 (3.84%)	732.5 (325 to 1666.5)	4.83 (3.65 to 6.40)
Arthrodesis	2 027	2712.5 (1222 to 4546)	71 (3.50%)	520 (301 to 1054)	3.91 (3.04 to 5.04)
Partial Trapeziectomy	894	1972 (929 to 4070)	22 (2.46%)	673.5 (296.5 to 1599.5)	2.63 (1.59 to 4.37)

[a] Further thumb base procedure only (intra-articular steroid injection or revision surgery)

For peer review only

Complications

Overall there was a low rate of registered serious local complications and systemic events requiring admission to hospital following surgery, with the risk of any serious local complication being 0.22% within 90 days, and 0.58% for systemic events within 90 days (Table 3; supplementary table 6). There was an insufficient number of cases to enable further analysis of factors associated with complications.

Table 3. Local complications and systemic events following all BTOA surgery

	Time	All surgery N (%[95%CI])
**Local complications**		
Wound dehiscence and wound infection	Within 30 days	12 (0.03% [0.01-0.05])
	Within 90 days	14 (0.03% [0.02-0.05])
Neurovascular injury	Within 30 days	79 (0.18% [0.15-0.23])
Any complication	Within 30 days	91 (0.21% [0.17-0.26]
	Within 90 days	93 (0.22% [0.17-0.26]
**Systemic events**		
Stroke	Within 30 days	9 (0.02% [0.01-0.04])
	Within 90 days	40 (0.09% [0.07-0.13])
Respiratory Tract infection	Within 30 days	70 (0.16% [0.13-0.21])
	Within 90 days	174 (0.40% [0.35-0.47])
Myocardial infarction	Within 30 days	17 (0.04% [0.02-0.06])
	Within 90 days	50 (0.11% [0.09-0.15])
DVT/PE	Within 30 days	18 (0.04% [0.02-0.07])
	Within 90 days	41(0.10% [0.07-0.13])
UTI	Within 30 days	30 (0.07% [0.05-0.10]
	Within 90 days	75 (0.17% [0.14-0.22])
Acute Renal Failure	Within 30 days	13 (0.03% [0.02-0.05])
	Within 90 days	44 (0.10% [0.07-0.14])
Any Systemic Event	Within 30 days	87 (0.20% [0.16-0.25])
	Within 90 days	250 (0.58% [0.51-0.66])
Any Systemic Event (excl UTI & ARF)	Within 30 days	44 (0.10% [0.07-0.14])
	Within 90 days	131 (0.30% [0.25-0.36])

Factors associated with further thumb base procedure

When considering the impact of age, sex, and sociodemographic factors, the risk of further thumb base procedure was increased for those in a younger age category of 40-49 years (adjusted subhazard ratio 1.53 [95%CI 1.10 to 2.13]) and for men (adjusted sHR 1.24 [1.01 to 1.53]) (Supplementary table 7) when adjusting for the other factors. When adjusting for surgical subtype in addition to age, sex and socioeconomic status to determine if surgical subtype had an impact on the risk of further procedure, the increased risk for men and those who underwent surgery at a younger age did not remain significant. This suggests that increased risk of further thumb base procedure was likely to be due to a greater proportion of younger male patients undergoing arthroplasty and arthrodesis (Supplementary table 8).

When adding in surgical subtype as a variable of interest into the main analysis, compared to those undergoing simple trapeziectomy, those undergoing arthroplasty and arthrodesis were around 2.5 times more likely to proceed to further thumb base procedure (arthroplasty 2.46 [1.77 to 3.42]; arthrodesis 2.41 [1.78 to 3.28]) after adjustment for age, sex and socioeconomic deprivation. The forest plot in Figure 4 shows the impact of age, sex, socioeconomic deprivation and surgical subtype seen upon the risk of undergoing a further procedure.

For peer review only - http://bmjopen.bmj.com/site/about/guidelines.xhtml

In the sensitivity analyses where each surgical subtype was run in a separate model adjusted for age sex and deprivation, for the risk of further thumb base procedure within each surgical subtype, an increased risk of further procedure was seen in men for those who underwent trapeziectomy alone when adjusted for age and deprivation (adjusted sHR 1.33 [1.02 to 1.73]). This was not seen in those undergoing LRTI, arthrodesis or arthroplasty, but noting there were fewer cases in these subgroups. Age and sociodemographic status did not appear significant in any of the surgical subgroups in regression analysis. (Supplementary tables 9-12).

DISCUSSION

Principle Findings

This study found that patients undergoing surgery for BTOA in the NHS in England are predominantly females and in their 7th decade. There has been an increase in surgery undertaken over the last 19 years. There was a low rate of revision surgery and intraarticular steroid injection registered following primary surgery in this cohort. In addition, there are low rates of registered serious local complications and systemic events requiring hospital admission within the immediate post-operative period. Further thumb base procedures mostly occurred within the first 18 months post operatively. The greatest factor influencing progression to further thumb base procedure in this population was surgical subtype, with patients receiving an arthroplasty around 2.5 times more likely to undergo further procedure than those having simple trapeziectomy.

Strengths and contribution to current knowledge

The major strength of this study is the national nature of the study setting. The HES APC dataset has been extensively used for evaluating the safety and outcomes following surgery for other musculoskeletal conditions, and this large national cohort of patients provides longitudinal follow up of individuals across multiple providers.[16-18] It allows retention in the study of those individuals who may have sought a second opinion at a different hospital in England. This gives a better overview of the need for revision surgery and for the management of serious adverse events following surgery than is possible in single centre or regional studies. As the private healthcare sector accounts for a low proportion of all healthcare activity in England, whilst some may seek consultation in this sector, all NHS activity will be captured within this dataset.[20] It also accounts for patient movement within the country, noting that emigration out of the UK is thought to be low at around 400,000 persons per year.[33]

This study includes all adult patients who underwent surgery in England, and is representative of the generality of hand surgery practice in a large public healthcare system. Patients at extremes of ages, with comorbid conditions that may render them ineligible to take part in clinical trials, are included. Due to practical and financial constraints, randomised control trials typically lack the statistical power and longevity of follow up to reliably inform on the safety of surgical procedures for BTOA. Large observational studies, such as this one, are important to fill that evidence gap. Previous studies have suggested increased revision risk in those of a younger age which we also detected here in a univariable analysis. The effect did not persist here in multivariable analysis, and may therefore be more directly related to choice of surgery type.[34, 35]

All widely used surgical procedure subtypes are included in this study, enabling a full comparison of each main procedure with the other. This facilitates broad comparison of the rate of registered complications and revision across an inclusive population with longer follow up than most clinical trials.[12, 13] This study emphasises that whilst the overall risk of BTOA surgery is low, there is an increased risk of further procedure for those undergoing arthroplasty that warrants further large scale epidemiological analysis, as has been seen in joint arthroplasty research at other anatomical sites.[36, 37]

For peer review only - http://bmjopen.bmj.com/site/about/guidelines.xhtml

Limitations and future work

This study focusses on the patient factors that can impact the risk of adverse outcome. It does not account for potential variation associated with surgeon or hospital factors. Further methodological research is underway to identify the best method of multilevel analysis that can explore the interaction of healthcare provider factors with patient factors. This technique may enable us to identify how multiple factors influence the risk of adverse events. Patients included here are those who have been registered within NHS Digital records, a dataset that is designed for remuneration rather than research. This means therefore that minor complications that do not require a minimum of a daycase admission or procedure will not be recorded. This study can identify the most serious complications and events that impact upon morbidity, but study of minor complications are better suited to electronic health care record or primary care data analysis.

Care was taken to validate the codes in two different populations used to identify patient records in order to limit misclassification bias, but this remains a potential limitation of the work. All arthroplasties are coded as a generic arthroplasty code, and therefore implant type cannot be analysed. Future work focussing on implant registries, such as currently used in hip and knee arthroplasty, is needed to determine the impact of implant type upon outcome in routine clinical practice.[38, 39] Members of our Department recently undertook a service evaluation project in 15 centres in the UK. [40] In this study of 150 patients, there was a 50:50 split between LRTI and simple trapeziectomy undertaken. However, this was undertaken in centres volunteering to participate in research, with data collected by surgeons, with a much smaller sample size and over a much shorter period of time (March 2017- May 2019). This study may therefore be prone to selection bias in a different way to the selection bias generated by using routinely collected data. In order to determine if we could adequately identify surgical subtypes from the HES APC dataset, we undertook two external validation studies after an iterative process of generating an included list of diagnosis and procedure codes. After these processes, we demonstrated that it was possible to differentiate between the surgical subtypes, including simple trapeziectomy and LRTI. Our data suggest that simple trapeziectomy was undertaken much more commonly than LRTI in England. Randomised control trials based in the UK and a Cochrane review have suggested little difference in efficacy between LRTI and simple trapeziectomy, and higher costs and complications associated with LRTI, and therefore the low proportion of LRTIs undertaken could reflect English surgeons adhering to UK evidence based practice.[2, 11, 13]

Comorbidities registered in this dataset are necessarily registered within an admitted patient episode, which may lead to inclusion bias of information used for remuneration. As the registration of comorbidities is likely to reflect the selection bias of only those who have received inpatient care for these conditions, these conditions were not used as factors that may influence the need for further thumb base procedures within the multivariable analysis. Whilst admitted patient care gives the best granularity of information for surgery and in-hospital events, it does not cover interactions occurring in an outpatient or primary care environment. Therefore, this study only identifies serious adverse events; those requiring at least a day case admission to hospital, and will not include those complication seen in primary care or outpatient facilities. It will fail to identify some pre-existing comorbidities in our patients, or persistent pain. Complications treated in the community such as minor surgical site infections requiring oral antibiotics will also not be registered within this dataset. Our future work is focussed upon identifying patients who have undergone BTOA surgery in primary care records, to compare the information collected there regarding comorbidity and post-operative infection.

For peer review only - http://bmjopen.bmj.com/site/about/guidelines.xhtml

Finally, this study uses revision surgery and serious adverse events as a proxy for outcome following surgery, as no patient reported outcomes (PROMS) are routinely collected for hand surgery at a national level by the NHS. Further work is needed to focus upon comparing PROMS for each surgical subtype in routine clinical practice to identify the quality of outcome from the patient perspective.

Meaning and use for clinical practice
As this study includes a national cohort of patients from routine clinical practice, the rates of further thumb base procedure and serious adverse events requiring in-patient hospital treatment given in this study are directly of use for counselling patients during the process of consent and shared decision making. The higher rates of further thumb base procedure following arthroplasty found in this study concurs with a previous systematic review of smaller trials.[12] Within a large cohort of patients, demographic factors did not appear to be associated with significantly increased risk of adverse outcomes when accounting for surgical subtype, which again is informative for both the surgeon and patient. The study suggests that there is a low overall rate of further procedures and serious complications following surgery for BTOA. Whilst the risk of further procedure does vary by surgical subtype, demographic factors do not appear to influence this risk.

FIGURE LEGENDS

Figure 1 Data processing flow chart
Figure 2 Age specific incidence of BTOA surgeries by year, England 1998-2017
Figure 3 Kaplan Meier plot of risk of further thumb base procedure after all BTOA surgery, divided by sex
Figure 4 Forest plot of relative risk of further thumb base procedure after primary BTOA surgery within multivariable regression analysis adjusted for age, sex, sociodemographic status and surgical subtype

ETHICAL APPROVAL

This study was registered at clinicaltrials.gov (NCT03573765) and approved by the University Research Services Clinical Trials Research Group (project ID 12787), and the NHS Data Access Advisory Group (DAAG). It was carried out in accordance with the NHS Digital Data Sharing Agreement (DARS-NIC-29827-Q8Z7Q). Studies using non-identifiable records from Hospital Episode Statistics are exempt from research ethics committee approval. Patients have the right to request that their data is not released by NHS Digital for use by researchers (register a “Type 2 opt-out”).

CONTRIBUTOR STATEMENT

JCEL, DF, RC, JLR, MG and DPA conceived the study and designed the study method. MM and NDR undertook the validation study. JCEL and RC acquired the data. JCEL performed the data analysis. JCEL, DF, RC, JLR, MG, NDR and DPA interpreted the data. JCEL drafted the manuscript. All authors reviewed and edited the final manuscript. JCEL, RC, JR and DF has full access to all data in the study. DF is the guarantor.

ACKNOWLEDGEMENTS

The authors would like to acknowledge Miss Rachel Kuo and Professor Jane Green for their work in this study.

ROLE OF THE FUNDING SOURCE

This work was supported by Versus Arthritis [21605] (JL); the Medical Research Council [MR/K501256/1] (JL); RCS England/ NJR research fellowship (RC); University of Oxford (JR); the National Institute for Health Research (NIHR) [SRF-2018-11- ST2-004] (DPA); NIHR Biomedical Research Centre, Oxford (BRC) (RC, DF, JR) and Oxford Medical Research Fund (MDG). No funders had a direct role in this study. The views and opinions expressed are those of the authors and do not necessarily reflect those of the Clinician

Scientist Award programme, NIHR, NHS or the Department of Health. This work used data supplied by NHS Digital copyright 2018, re-used with the permission of NHS Digital. All rights reserved.

COMPETING INTERESTS

All authors have completed an ICJME conflict of interest form that is uploaded with the study (http://www.icmje.org/conflicts-ofinterest/) no support from any organization for the submitted work; D P-A has received research grants from Amgen, Servier, UCB; departmental fees for speaker services from Amgen, departmental fees for consultancy from UCB.JL reports grants from the Medical Research Council (MR/K501256/1) and Versus Arthritis (21605), outside of the submitted work. NR reports personal fees from Acumed, outside the submitted work; Dr. Craig reports grants from Royal College of Surgeons/National Joint Registry, outside the submitted work. No other relationships or activities could appear to have influenced the submitted work.

TRANSPARENCY STATEMENT

The lead author affirms that the manuscript is an honest, accurate, and transparent account of the study being reported; no important aspects of the study have been omitted, and that any discrepancies from the study as originally planned have been explained.

DATA SHARING STATEMENT

No further data can be made available due to NHS Digital restrictions. Data extracts can be applied for via the NHS Digital data access request service (https://digital.nhs.uk/services/data-access-request-service-dars).

EXECUTIVE LICENSE

The Corresponding Author has the right to grant on behalf of all authors and does grant on behalf of all authors, a worldwide licence (http://www.bmj.com/sites/default/files/BMJ%20Author%20Licence%20March%202013.doc) to the Publishers and its licensees in perpetuity, in all forms, formats and media (whether known now or created in the future), to i) publish, reproduce, distribute, display and store the Contribution, ii) translate the Contribution into other languages, create adaptations, reprints, include within collections and create summaries, extracts and/or, abstracts of the Contribution and convert or allow conversion into any format including without limitation audio, iii) create any other derivative work(s) based in whole or part on the on the Contribution, iv) to exploit all subsidiary rights to exploit all subsidiary rights that currently exist or as may exist in the future in the Contribution, v) the inclusion of electronic links from the Contribution to third party material where-ever it may be located; and, vi) licence any third party to do any or all of the above. All research articles will be made available on an open access basis (with authors being asked to pay an open access fee—see http://www.bmj.com/about-bmj/resources-authors/forms-policies-and-checklists/copyright-open-access-and-permission-reuse). The terms of such open access shall be governed by a Creative Commons licence—details as to which Creative Commons licence will apply to the research article are set out in our worldwide licence referred to above.

REFERENCES

1. Yuan F, Aliu O, Chung KC, Mahmoudi E. Evidence-Based Practice in the Surgical Treatment of Thumb Carpometacarpal Joint Arthritis. J Hand Surg Am. 2017;42(2):104-12 e1.
2. Field J, Buchanan D. To suspend or not to suspend: a randomised single blind trial of simple trapeziectomy versus trapeziectomy and flexor carpi radialis suspension. Journal of Hand Surgery: European Volume. 2007;32:462-6.
3. Thorkildsen RD, Røkkum M. Trapeziectomy with LRTI or joint replacement for CMC1 arthritis, a randomised controlled trial. J Plast Surg Hand Surg. 2019;53(6):361-9.

4. Hernández-Cortés P, Pajares-López M, Robles-Molina MJ, Gómez-Sánchez R, Toledo-Romero MA, De Torres-Urrea J. Two-year outcomes of Elektra prosthesis for trapeziometacarpal osteoarthritis: a longitudinal cohort study. J Hand Surg Eur Vol. 2012;37(2):130-7.
5. Vissers G, Goorens CK, Vanmierlo B, Bonte F, Mermuys K, Fils JF, et al. Ivory arthroplasty for trapeziometacarpal osteoarthritis: 10-year follow-up. J Hand Surg Eur Vol. 2019;44(2):138-45.
6. Cootjans K, Vanhaecke J, Dezillie M, Barth J, Pottel H, Stockmans F. Joint Survival Analysis and Clinical Outcome of Total Joint Arthroplasties With the ARPE Implant in the Treatment of Trapeziometacarpal Osteoarthritis With a Minimal Follow-Up of 5 Years. J Hand Surg Am. 2017;42(8):630-8.
7. Toffoli A, Teissier J. MAÏA Trapeziometacarpal Joint Arthroplasty: Clinical and Radiological Outcomes of 80 Patients With More than 6 Years of Follow-Up. J Hand Surg Am. 2017;42(10):838 e1- e8.
8. Dehl M, Chelli M, Lippmann S, Benaissa S, Rotari V, Moughabghab M. Results of 115 Rubis II reverse thumb carpometacarpal joint prostheses with a mean follow-up of 10 years. J Hand Surg Eur Vol. 2017;42(6):592-8.
9. Barrera-Ochoa S, Vidal-Tarrason N, Correa-Vázquez E, Reverte-Vinaixa MM, Font-Segura J, Mir-Bullo X. Pyrocarbon interposition (PyroDisk) implant for trapeziometacarpal osteoarthritis: minimum 5-year follow-up. J Hand Surg Am. 2014;39(11):2150-60.
10. van Cappelle HG, Deutman R, van Horn JR. Use of the Swanson silicone trapezium implant for treatment of primary osteoarthritis : long-term results. J Bone Joint Surg Am. 2001;83(7):999-1004.
11. Gangopadhyay S, McKenna H, Burke FD, Davis TR. Five- to 18-year follow-up for treatment of trapeziometacarpal osteoarthritis: a prospective comparison of excision, tendon interposition, and ligament reconstruction and tendon interposition. J Hand Surg Am. 2012;37(3):411-7.
12. Ganhewa AD, Wu R, Chae MP, Tobin V, Miller GS, Smith JA, et al. Failure Rates of Base of Thumb Arthritis Surgery: A Systematic Review. J Hand Surg Am. 2019;44(9):728-41 e10.
13. Wajon A, Carr E, Edmunds I, Ada L. Surgery for thumb (trapeziometacarpal joint) osteoarthritis. Cochrane Database Syst Rev. 2009(4):CD004631.
14. Billig JI, Nasser JS, Chung KC. National Prevalence of Complications and Cost of Small Joint Arthroplasty for Hand Osteoarthritis and Post-Traumatic Arthritis. J Hand Surg Am. 2020;45(6):553 e1- e12.
15. Makady A, van Veelen A, Jonsson P, Moseley O, D'Andon A, de Boer A, et al. Using Real-World Data in Health Technology Assessment (HTA) Practice: A Comparative Study of Five HTA Agencies. PharmacoEconomics. 2018;36(3):359-68.
16. Abram SGF, Judge A, Beard DJ, Price AJ. Adverse outcomes after arthroscopic partial meniscectomy: a study of 700 000 procedures in the national Hospital Episode Statistics database for England. Lancet. 2018;392(10160):2194-202.
17. Craig RS, Lane JCE, Carr AJ, Furniss D, Collins GS, Rees JL. Serious adverse events and lifetime risk of reoperation after elective shoulder replacement: population based cohort study using hospital episode statistics for England. Bmj. 2019;364:l298.
18. Alser O, Craig RS, Lane JCE, Prats-Uribe A, Robinson DE, Rees JL, et al. Serious complications and risk of re-operation after Dupuytren's disease surgery: a population-based cohort study of 121,488 patients in England. Sci Rep. 2020;10(1):16520.
19. Digital N. Hospital Episode Statistics. 2018 [Available from: https://digital.nhs.uk/data-and-information/data-tools-and-services/data-services/hospital-episode-statistics.
20. Kings Fund. The UK private health market. London: Kings Fund; 2014.
21. Digital N. Linked HES-ONS mortality data 2018 [Available from: https://digital.nhs.uk/data-and-information/data-tools-and-services/data-services/linked-hes-ons-mortality-data#ons-mortality-data.
22. WHO. International Classification of Disease version 10 2016 [2016:[Available from: https://www.who.int/classifications/icd/icdonlineversions/en/.
23. Digital N. National clinical coding standards: OPCS-4 and ICD-10 London2017 [Available from: https://digital.nhs.uk/services/terminology-and-classifications/clinical-classifications.
24. Lane JCE SC, Green J, Lam WL, Furniss D, Sudlow C. Can we use routinely collected hospital and GP data for epidemiological study of common hand conditions? A UK Biobank based validation project. Biorxiv [Internet]. 2018.
25. Ministry of Housing CaLG. English indices of Deprivation: UK Government; 2018 [Available from: https://www.gov.uk/government/collections/english-indices-of-deprivation.
26. Quan H, Sundararajan V, Halfon P, Fong A, Burnand B, Luthi JC, et al. Coding algorithms for defining comorbidities in ICD-9-CM and ICD-10 administrative data. Medical care. 2005;43(11):1130-9.

For peer review only - http://bmjopen.bmj.com/site/about/guidelines.xhtml

27. Quan H, Li B, Couris CM, Fushimi K, Graham P, Hider P, et al. Updating and validating the Charlson comorbidity index and score for risk adjustment in hospital discharge abstracts using data from 6 countries. American journal of epidemiology. 2011;173(6):676-82.
28. Digital N. Hospital Episode Statistics Data Dictionary. In: Care HaS, editor. 2020.
29. Digital N. NHS Outcomes Framework 2018 [Available from: https://digital.nhs.uk/data-and-information/publications/ci-hub/nhs-outcomes-framework.
30. ONS. Population estimates for the UK, England and Wales, Scotland and Northern Ireland 2018 [Available from: https://www.ons.gov.uk/peoplepopulationandcommunity/populationandmigration/populationestimates/datasets/populationestimatesforukenglandandwalesscotlandandnorthernireland.
31. Fine JP, Gray R.J. A proportional hazards model for the subdistribution of a competing risk. Journal of the American Statistical Association. 1999;94:496-509.
32. James Lind Alliance. Common Conditions Affecting the Hand and Wrist 2017 [Available from: http://www.jla.nihr.ac.uk/priority-setting-partnerships/common-conditons-affecting-the-hand-and-wrist/ [last accessed 27.04.2020].
33. ONS. Provisional long-term international migration estimates. 2020.
34. Mattila S, Waris E. Revision of trapeziometacarpal arthroplasty: risk factors, procedures and outcomes. Acta Orthop. 2019;90(4):389-93.
35. Wilkens SC, Xue Z, Mellema JJ, Ring D, Chen N. Unplanned Reoperation After Trapeziometacarpal Arthroplasty: Rate, Reasons, and Risk Factors. Hand (N Y). 2017;12(5):446-52.
36. Partridge TCJ, Baker PN, Jameson SS, Mason J, Reed MR, Deehan DJ. Conventional Versus Highly Cross-Linked Polyethylene in Primary Total Knee Replacement: A Comparison of Revision Rates Using Data from the National Joint Registry for England, Wales, and Northern Ireland. J Bone Joint Surg Am. 2020;102(2):119-27.
37. Penfold CM, Blom AW, Sayers A, Wilkinson JM, Hunt L, Judge A, et al. Understanding the uptake of new hip replacement implants in the UK: a cohort study using data from the National Joint Registry for England and Wales. BMJ Open. 2019;9(11):e029572.
38. BOA. Trauma and Orthopaedic Registries Unifying Structure (TORUS) 2020 [Available from: https://www.boa.ac.uk/policy-engagement/registries-and-outcomes/trauma-orthopaedic-registries-unifying-structure.html [last accessed 24.06.2020].
39. BSSH. UK Hand Registry 2018 [Available from: https://secure.amplitude-registry.com/BSSH/Account/Login?ReturnUrl=%2fBSSH.
40. Parker S, Riley N, Dean B. Management of osteoarthritis at the base of the thumb. Bone Joint J. 2020;102-B(5):600-5.

For peer review only

For peer review only - http://bmjopen.bmj.com/site/about/guidelines.xhtml

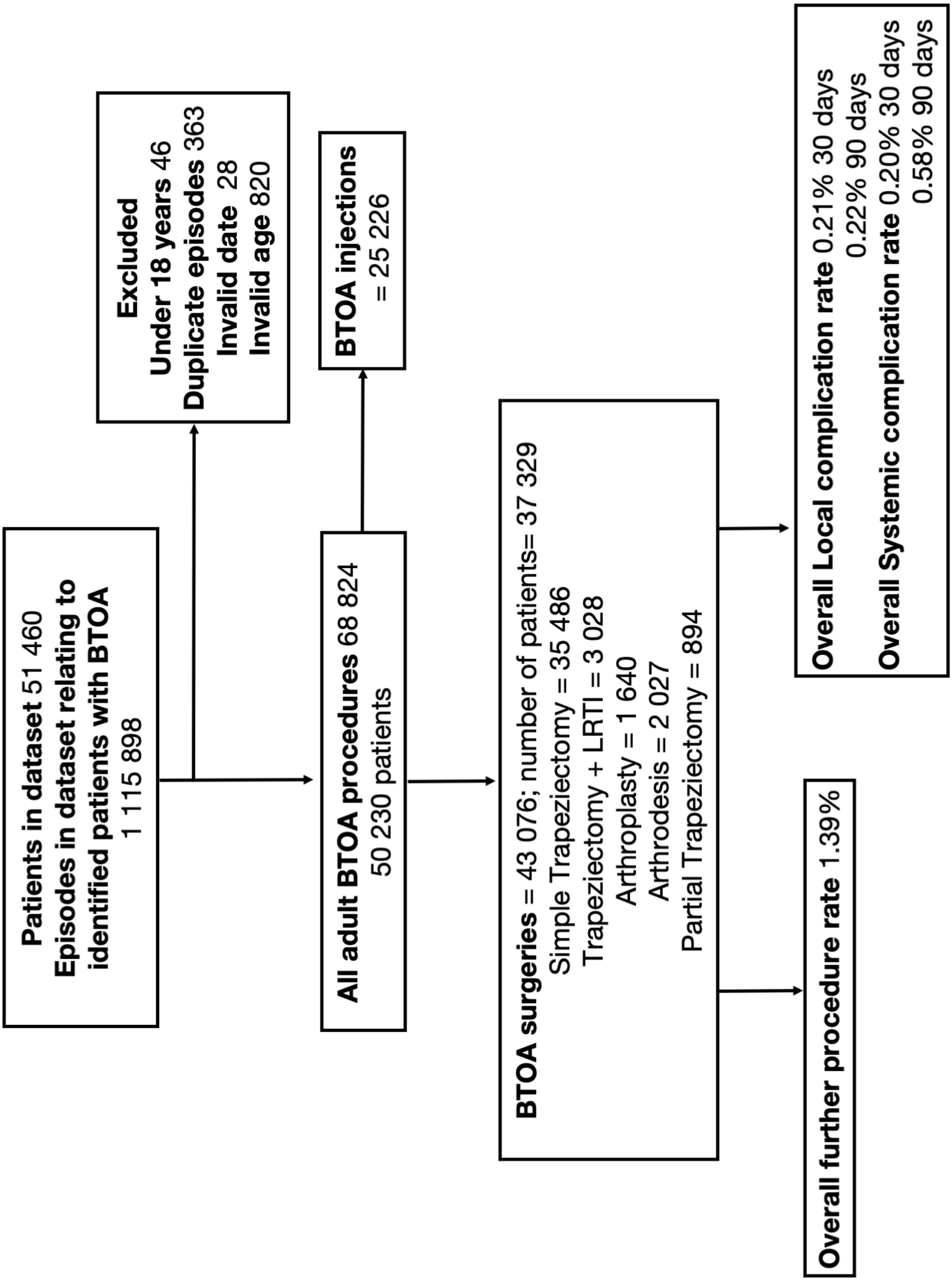
Patients in dataset 51 460
Episodes in dataset relating to identified patients with BTOA
1 115 898
Excluded
Under 18 years 46
Duplicate episodes 363
Invalid date 28
Invalid age 820
All adult BTOA procedures 68 824
50 230 patients
BTOA injections
= 25 226
BTOA surgeries = 43 076; number of patients= 37 329
Simple Trapeziectomy = 35 486
Trapeziectomy + LRTI = 3 028
Arthroplasty = 1 640
Arthrodesis = 2 027
Partial Trapeziectomy = 894
Overall Local complication rate 0.21% 30 days
0.22% 90 days
Overall Systemic complication rate 0.20% 30 days
0.58% 90 days
Overall further procedure rate 1.39%

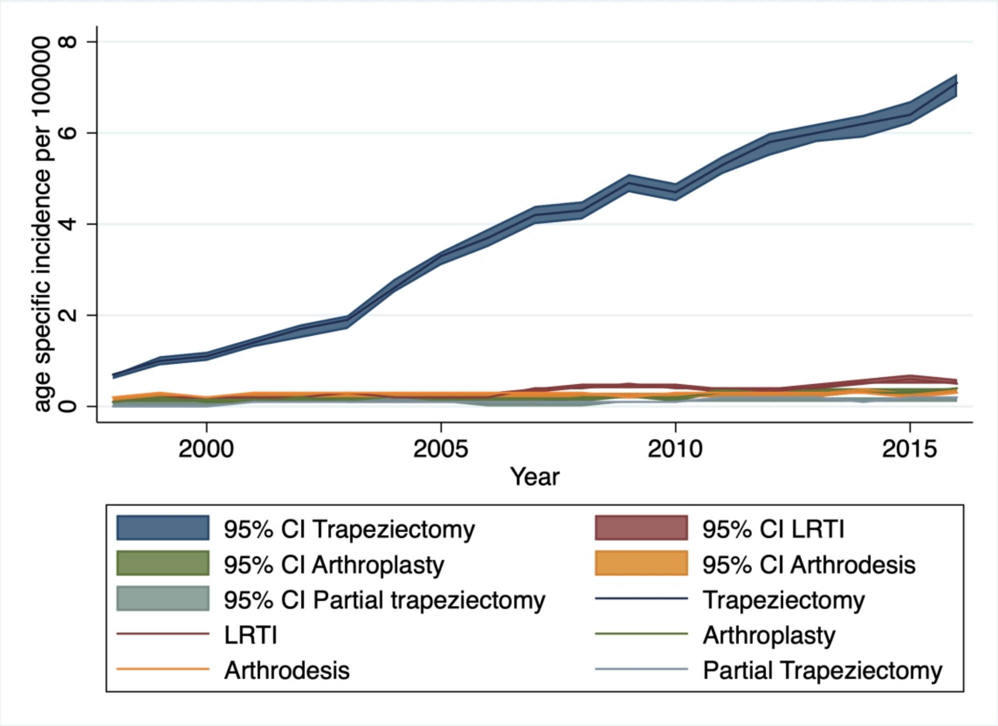

395x287mm (144 x 144 DPI)

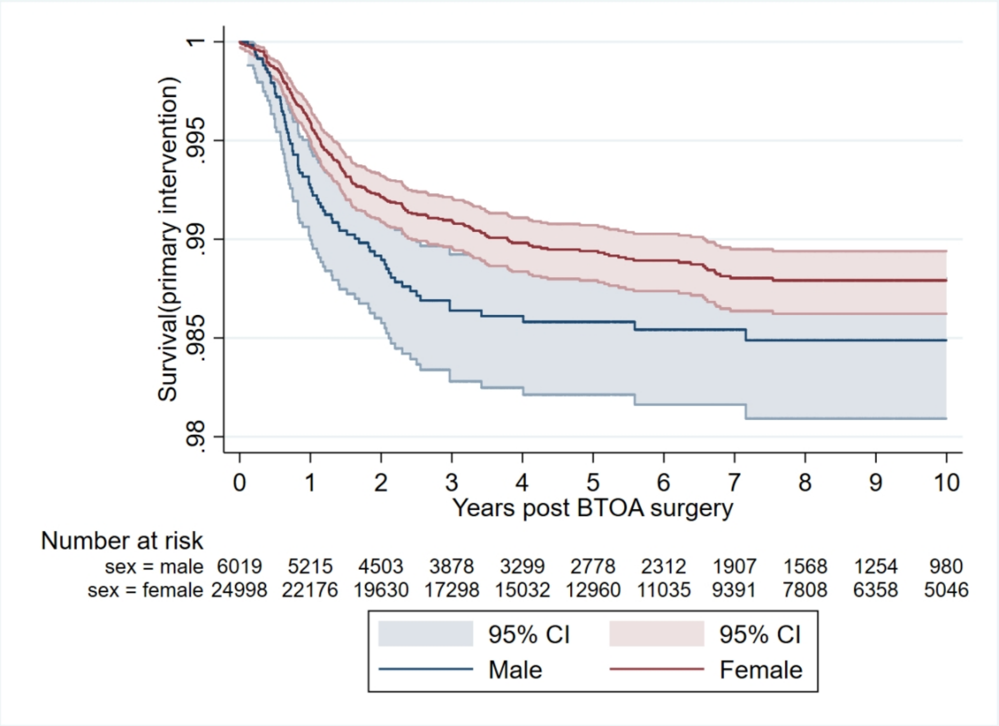

450x327mm (72 x 72 DPI)

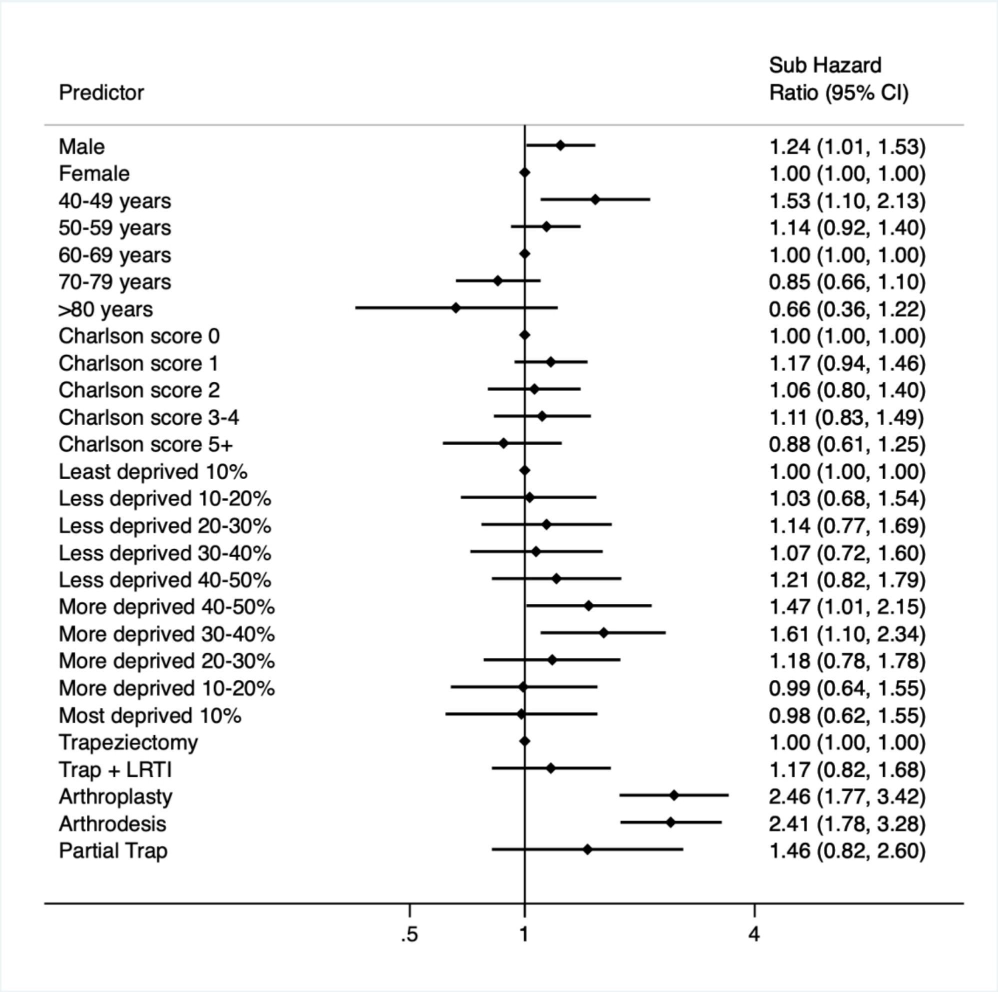

289x287mm (144 x 144 DPI)

Supplementary appendix Low rate of serious complications and further procedures following with BTOA surgery: analysis of national cohort of 43 076 surgeries with up to 19 years of follow up

Supplementary table 1 OPCS and ICD codes used to identify BTOA surgery

**BTOA OPCS codes**			**BTOA ICD-10 codes**
W57.2	alone		M18.0-18.9
W57.3	alone		M15.1-2
W57.8	**WITH ANY**	Z82.3	
W02.8		Z72.4	
W02.9		Z73.8	
W06.8		Z73.9	
W06.9		Z89.5	
W08.5		Z82.2	
W08.8		Z82.9	
W08.9			
W13.8			
W17.8			
W17.9			
W28.1			
W33.8			
W33.9			
W43.1			
W44.1			
W45.1			
W45.3			
W54.1			
W54.3			
W56.2			
W57.2			
W62.1			
W62.2			
W62.8			
W62.9			
W63.1			
W63.2			

For peer review only

W63.8		
W63.9		
W64.1		
W64.2		
W64.8		
W64.9		
W74.2		
W74.3		
W77.5		
W77.7		
W77.8		
W90.3		
W90.4		

Supplementary Table 2 Covariate codes

**Covariates for BTOA**	**ICD-10 code**
CTS	G560 (or OPCS A651)
Knee OA	M17
Generalised OA	M15; M19
Rheumatoid Arthritis	M05
Wrist/Hand fracture	S62
Oophorectomy	(OPCS Q22,Q23, Q43)

Supplementary Table 3 Complication codes for systemic complications and local complications

**Complications**	**code**
Wound dehiscence	OPCS Z82 + (S60.4 or S42.2 or S42.3 or S42.4)
Tendon repair	OPCS Z82 + T67
Wound debridement	OPCS Z82 + (T96.8 or T96.3 or T96.4)
Neurovascular injury	ICD code T81.2
Stroke	ICD I60X;I610-I619; I630-I639; I64X
Respiratory Tract Infection	ICD J12X J13, J14, J15X J180-J182 J188; J189; J22; J440 J441 J690 J691 J698 J851
Myocardial Infarction	ICD I210-I214; I219
Deep Vein Thrombosis/ Pulmonary Embolus	ICD I801-I803; I260; I269
Urinary tract infection/ acute renal failure	ICD N300 N390 N170-N172; N178 N179

For peer review only

Supplementary Table 4 Comparison of patients with and without laterality (baseline demographics)

	**With laterality**	**Without laterality**
**Total**	36 072	7004
**Sex**		
Male	7592 (21.05)	1365 (19.49)
Female	28475 (78.94)	5637 (80.48)
Missing	5 (0.01)	2 (0.03)
**Mean age (SD; years)**	63.04 (9.15)	63.13 (9.26)
**Charlson index**		
0	15365 (42.60)	2914 (41.60)
1	8937 (24.78)	1703 (24.31)
2	4746 (13.16)	947 (13.52)
3	2730 (7.57)	556 (7.94)
4	1372 (3.80)	287 (4.10)
>=5	760 (8.10)	166 (8.52)
missing	0	0
**IMD decile**		
Least deprived 10%	4103 (11.37)	646 (9.22)
Less deprived 10-20%	4041 (11.20)	728 (10.39)
Less deprived 20-30%	4055 (11.24)	764 (10.91)
Less deprived 30-40%	4023 (11.15)	795 (11.35)
Less deprived 40-50%	3947 (10.94)	735 (10.49)
More deprived 10-20%	2681 (7.43)	598 (8.54)
More deprived 20-30%	3125 (8.66)	673 (9.61)
More deprived 30-40%	3487 (9.67)	719 (10.27)
More deprived 40-50%	3820 (10.59)	739 (10.55)
Most deprived 10%	2464 (6.83)	575 (8.21)
Missing	326 (0.90)	32 (0.46)
**Ethnic group**		
Any white background	31107 (86.24)	6097 (87)
Any Asian background	380 (1.05)	68 (1)
Any Black background	71 (0.2)	12 (<1)
Any mixed background	51 (0.14)	9 (<1)
Chinese	19 (0.05)	<7*
Any other ethnic group	127 (0.35)	28 (<1)
Not stated	3639 (10.08)	650 (9)
Not known	678 (1.88)	139 (2)
missing	0	0
**Co variates**		
Carpal Tunnel Syndrome	3989 (11.06)	794 (11.34)
Knee Osteoarthritis	4559 (12.64)	936 (13.36)
General Osteoarthritis	7569 (20.98)	1667 (23.8)
Rheumatoid Arthritis	84 (0.23)	25 (0.36)
Wrist Fracture	206 (0.57)	43 (0.61)
Oophorectomy	1351 (3.74)	232 (3.31)

*Numbers less than 7 suppressed in line with NHS Digital disclosure control guidelines- percentages of other groups rounded to prevent secondary disclosure of data (29)

For peer review only

Supplementary table 5 Baseline demographics of all patients undergoing BTOA surgery

	**All surgery (N, %) (total=43 076)**	**Trapeziectomy N (%) (35 486)**	**LRTI N (%) (3028)**	**Arthroplasty N (%) (1640)**	**Arthrodesis N (%) (2027)**	**Partial Trapeziectomy N (%) (894)**
**Sex**						
Male	8957 (20.7)	6 826 (19.2)	616 (20.3)	390 (23.8)	934 (46.1)	191 (21.3)
Female	34 112 (79.1)	28 655 (80.8)	2411 (79.6)	1250 (76.2)	1092 (53.9)	704 (78.7)
Missing	7 (0.02)	5 (0.01)	1 (0.03)	0	1 (0.05)	0
**Mean age (SD; years)**	63.2 (SD 9.2)	63.6 (SD 8.8)	62.5 (SD 9.7)	61.7 (SD 9.3)	58.1 (11.7)	63.1 (9.8)
**Charlson index**						
0	18 279 (42.4)	14 992 (42.3)	1 324 (43.8)	677 (41.3)	890 (43.9)	396 (44.3)
1	10 640 (24.7)	8 807 (24.8)	721 (23.8)	444 (27.1)	475 (23.4)	193 (21.6)
2	5 693 (13.2)	4 746 (13.4)	392 (13.0)	199 (12.1)	250 (12.3)	106 (11.8)
3	3 286 (7.6)	2 723 (7.7)	222 (7.3)	125 (7.6)	142 (7.0)	74 (8.3)
4	1 659 (3.9)	1 359 (3.8)	123 (4.1)	60 (3.7)	77 (3.8)	40 (4.5)
>=5	3 519 (8.2)	2 859 (8.0)	246 (8.1)	135 (8.2)	193 (9.6)	86 (8.5)
missing	0	0	0	0	0	
**IMD decile**						
Least deprived 10%	4 749 (11.0)	4 015 (11.3)	268 (8.8)	203 (12.4)	153 (7.6)	110 (12)
Less deprived 10-20%	4 769 (11.1)	3 934 (11.1)	314 (10.4)	232 (14.15)	195 (9.6)	94 (10)
Less deprived 20-30%	4 819 (11.2)	3 976 (11.2)	354 (11. 5)	185 (11.3)	212 (10.5)	92 (10)
Less deprived 30-40%	4 818 (11.2)	3 974 (11.2)	393 (13.0)	157 (9.6)	212 (10.5)	82 (9)
Less deprived 40-50%	4 682 (10.9)	3 853 (10.9)	345 (11.4)	154 (9.4)	221 (10.9)	109 (12)
More deprived 10-20%	3 279 (7.61)	2 700 (7.61)	218 (7.2)	110 (6.7)	181 (8.9)	70 (7)
More deprived 20-30%	3 798 (8.8)	3 108 (8.8)	273 (9.0)	145 (8.8)	202 (10.0)	70 (7)
More deprived 30-40%	4 206 (9.8)	3 442 (9.7)	303 (10.1)	164 (10.0)	215 (10.6)	82 (9)
More deprived 40-50%	4 559 (10.6)	3 700 (10.4)	354 (11.7)	172 (10.5)	241 (11.9)	92 (10)
Most deprived 10%	3039 (7.1)	2 529 (7.1)	160 (5.3)	110 (6.7)	152 (7.5)	88 (9)
Missing	358 (0.8)	255 (0.7)	46 (1.5)	8 (0.5)	43 (2.1)	<7*
**Ethnic group**						
Any white background	37 204 (86.4)	30 738 (86.6)	2628 (86.8)	1410 (86.0)	1640 (80.9)	755 (84.4)
Any Asian background	439 (1.0)	376 (1.1)	24 (0.8)	20 (1.2)	16 (0.8)	12 (1.3)
Any Black background	92 (0.2)	60 (0.2)	<7*	<7*	12 (0.6)	<7*
Any mixed background	60 (0.1)	46 (0.1)	<7*	<7*	<7*	<7*
Chinese	20 (0.1)	15 (0.05)	<7*	<7*	<7*	<7*
Any other ethnic group	155 (0.4)	130 (0.36)	9 (0.3)	<7*	<7*	<7*
Not stated	1237 (2.9)	3 467 (9.8)	301 (10.0)	134 (8.2)	299 (14.8)	88 (9.8)
Not known	3 869 (9.0)	654 (1.8)	58 (1.9)	26 (1.6)	51 (2.5)	28 (3.1)
Missing	0	0	0	0	0	0
	43 076	35 486	3028	1640	2027	895
**Co variates**						
Carpal Tunnel Syndrome	4 783	3 998 (11.2)	337 (10.9)	178 (10.9)	178 (8.8)	92 (10.3)
Knee Osteoarthritis	5 495	4 643 (13.0)	368 (11.9)	176 (10.7)	205 (10.1)	103 (11.5)
General Osteoarthritis	9 236	7 663 (21.4)	641 (20.8)	378 (23.0)	382 (18.8)	172 (19.2)
Rheumatoid Arthritis	109	81 (0.2)	7 (0.2)	7 (0.4)	12 (0.6)	2 (0.2)
Wrist Fracture	249	183 (0.5)	17 (0.6)	3 (0.2)	39 (1.9)	7 (0.8)
Oophorectomy	1 583	1 371 (3.8)	83 (2.7)	53 (3.2)	50 (2.5)	26 (2.9)

*Numbers less than 7 suppressed in line with NHS Digital disclosure control guidelines- percentages of other groups rounded to prevent secondary disclosure of data (29)

For peer review only

For peer review only - http://bmjopen.bmj.com/site/about/guidelines.xhtml

Supplementary Table 6 Local and systemic complications per surgery subtype

	Time	All surgery N (%[95%CI])	Trapeziectomy N (%, 95%CI)	LRTI N (%, 95%CI)	Arthroplasty N (%, 95%CI)	Arthrodesis N (%, 95%CI)	Partial Trapeziectomy N (%, 95%CI)
**Local complications**							
Wound dehiscence and wound infection	Within 30 days	12 (0.03%, [0.01-0.05])	10 (0.03% [0.01-0.05])	<7*	0	<7*	0
	Within 90 days	14 (0.03% [0.02-0.05])	12 (0.03%[0.02-0.06])	<7*	0	<7*	0
Neurovascular injury	Within 30 days	79 (0.18% [0.15-0.23])	68 (0.19%[0.15-0.24])	<7*	0	<7*	<7*
Any complication	Within 30 days	91 (0.21% [0.17-0.26])	78 (0.22%[0.17-0.27])	<7*	0	<7*	<7*
	Within 90 days	93 (0.22% [0.17-0.26])	80 (0.23%[0.18-0.28])	<7*	0	<7*	<7*
**Systemic complications**							
Stroke	Within 30 days	9 (0.02% [0.01-0.04])	9 (0.03% [0.01-0.05])	0	0	0	0
	Within 90 days	40 (0.09% [0.07-0.13])	34 (0.10%[0.07-0.13])	<7*	<7*	<7*	<7*
Respiratory Tract infection	Within 30 days	70 (0.16% [0.13-0.21])	67 (0.19%[0.15-0.24])	<7*	0	0	<7*
	Within 90 days	174 (0.40% [0.35-0.47])	144 (0.41%[0.34-0.48])	10 (0.33%[0.16-0.61])	8 (0.49%[0.21-0.96])	<7*	<7*
Myocardial infarction	Within 30 days	17 (0.04% [0.02-0.06])	15 (0.04% [0.02-0.07])	<7*	0	0	0
	Within 90 days	50 (0.11% [0.09-0.15])	40 (0.11% [0.08-0.15])	<7*	0	<7*	<7*
DVT/PE	Within 30 days	18 (0.04% [0.02-0.07])	9 (0.03% [0.01-0.05])	<7*	<7*	0	0
	Within 90 days	41 (0.10% [0.07-0.13])	30 (0.08%[0.06-0.12])	<7*	<7*	0	<7*
UTI	Within 30 days	30 (0.07% [0.05-0.10])	25 (0.07%[0.05-0.10])	<7*	0	<7*	0
	Within 90 days	75 (0.17%[0.14-0.22])	62 (0.17% [0.13-0.22])	9 (0.30%[0.14-0.56])	0	<7*	<7*
Acute Renal Failure	Within 30 days	13 (0.03%[0.02-0.05])	9 (0.03%[0.01-0.05])	<7*	0	<7*	0
	Within 90 days	44 (0.10%[0.07-0.14])	40 (0.11 [0.08-0.15])	<7*	0	<7*	0
Any Systemic Complication	Within 30 days	87 (0.20%[0.16-0.25])	67 (0.19% [0.15-0.24])	10 (0.33%[0.16-0.61])	<7*	<7*	0
	Within 90 days	250 (0.58% [0.51-0.66])	206 (0.58% [0.50-0.67])	24 (0.79%[0.51-0.12])	<7*	<7*	<7*
Any Systemic Complication (excl UTI & ARF)	Within 30 days	44 (0.10% [0.07-0.14])	33 (0.10% [0.06-0.13])	<7*	<7*	0	0
	Within 90 days	131 (0.30%[0.25-0.36])	104 (0.29% [0.24-0.36])	13 (0.43%[0.23-0.73])	<7*	<7*	<7*

DVT/PE deep vein thrombosis or pulmonary embolic disease
UTI urinary tract infection
ARF Acute Renal Failure
<7 Numbers less than 7 suppressed in line with NHS Digital disclosure control guidelines (29)

Supplementary Table 7 Risk of revision following any BTOA surgery Multivariable regression analysis accounting for competing risk of mortality (without surgical subtype as a risk in the model)

ALL SURGERIES	**Crude sHR[a]**	**95% CI**	**Adj sHR[a]**	**95% CI**
**Male**	1.22	0.99 to 1.49	1.24	1.01 to 1.52
**Age**				
40-49 years	1.53	1.11 to 2.12	1.53	1.10 to 2.13
50-59 years	1.12	0.91 to 1.38	1.14	0.92 to 1.40
60-69 years	1 (ref)	1 (ref)	1 (ref)	1 (ref)
70-79 years	0.86	0.66 to 1.10	0.85	0.66 to 1.10
>80 years	0.66	0.36 to 1.21	0.66	0.36 to 1.22
**Charlson comorbidity index**				
0	1 (ref)	1 (ref)	1 (ref)	1 (ref)
1	1.14	0.92 to 1.42	1.17	0.94 to 1.46
2	0.99	0.75 to 1.31	1.06	0.80 to 1.40
3+	0.92	0.73 to 1.17	1.01	0.78 to 1.30
**IMD**				
Least deprived 10%	1 (ref)	1 (ref)	1 (ref)	1 (ref)
Less deprived 10-20%	1.03	0.69 to 1.54	1.03	0.68 to 1.54
Less deprived 20-30%	1.16	0.78 to 1.72	1.14	0.77 to 1.70
Less deprived 30-40%	1.08	0.73 to 1.61	1.07	0.72 to 1.60
Less deprived 40-50%	1.24	0.84 to 1.83	1.21	0.82 to 1.79
More deprived 40-50%	1.04	0.66 to 1.62	0.99	0.63 to 1.55
More deprived 30-40%	1.24	0.82 to 1.86	1.18	0.79 to 1.78
More deprived 20-30%	1.66	1.14 to 2.42	1.61	1.10 to 2.34
More deprived 10-20%	1.51	1.04 to 2.20	1.47	1.01 to 2.15
Most deprived 10%	1.03	0.65 to 1.63	0.98	0.62 to 1.55
[a] Subhazard ratio				

For peer review only

Supplementary Table 8 Risk of revision following any BTOA surgery Multivariable regression analysis accounting for competing risk of mortality, including surgical subtype as a factor

ALL SURGERIES, including SURG SUBTYPE	**Crude sHR[a]**	**95% CI**	**Adj sHR[a]**	**95% CI**
**Male**	1.22	0.99 to 1.49	1.11	0.89 to 1.38
**Age**				
40-49 years	1.53	1.11 to 2.12	1.24	0.95 to 1.87
50-59 years	1.12	0.91 to 1.38	1.09	0.88 to 1.34
60-69 years	1 (ref)	1 (ref)	1 (ref)	1 (ref)
70-79 years	0.86	0.66 to 1.10	0.86	0.67 to 1.11
>80 years	0.66	0.36 to 1.21	0.67	0.37 to 1.24
**Charlson comorbidity index**				
0	1 (ref)	1 (ref)	1 (ref)	1 (ref)
1	1.14	0.92 to 1.42	1.17	0.94 to 1.46
2	0.99	0.75 to 1.31	1.05	0.79 to 1.40
3+	0.92	0.73 to 1.17	0.99	0.77 to 1.28
**IMD**				
Least deprived 10%	1 (ref)	1 (ref)	1 (ref)	1 (ref)
Less deprived 10-20%	1.03	0.69 to 1.54	1.00	0.67 to 1.50
Less deprived 20-30%	1.16	0.78 to 1.72	1.13	0.76 to 1.67
Less deprived 30-40%	1.08	0.73 to 1.61	1.06	0.71 to 1.58
Less deprived 40-50%	1.24	0.84 to 1.83	1.19	0.81 to 1.77
More deprived 40-50%	1.04	0.66 to 1.62	0.98	0.63 to 1.53
More deprived 30-40%	1.24	0.82 to 1.86	1.16	0.77 to 1.75
More deprived 20-30%	1.66	1.14 to 2.42	1.58	1.09 to 2.29
More deprived 10-20%	1.51	1.04 to 2.20	1.45	1.00 to 2.11
Most deprived 10%	1.03	0.65 to 1.63	0.96	0.61 to 1.52
**Surgery subtype**				
Simple trapeziectomy	1 (ref)	1 (ref)	1 (ref)	1 (ref)
Trapeziectomy + LRTI	1.22	0.86 to 1.73	1.16	0.82 to 1.66
Arthroplasty	2.51	1.81 to 3.48	2.49	1.80 to 3.44
Arthrodesis	2.55	1.91 to 3.40	2.40	1.78 to 3.26
Partial Trapeziectomy	1.44	0.81 to 2.55	1.46	0.82 to 2.59
[a] Subhazard ratio				

For peer review only

Supplementary Table 9 Risk of revision following Trapeziectomy Multivariable regression analysis accounting for competing risk of mortality

TRAPEZIECTOMY	**Crude sHR[a]**	**95% CI**	**Adj sHR[a]**	**95% CI**
**Male**	1.31	1.01 to 1.70	1.32	1.02 to 1.72
**Age**				
40-49 years	1.21	0.76 to 1.93	1.20	0.74 to 1.92
50-59 years	0.96	0.74 to 1.25	0.97	0.73 to 1.27
60-69 years	1 (ref)	1 (ref)	1 (ref)	1 (ref)
70-79 years	0.88	0.65 to 1.19	0.92	0.67 to 1.24
>80 years	0.53	0.23 to 1.19	0.55	0.24 to 1.25
**Charlson comorbidity index**				
0	1 (ref)	1 (ref)	1 (ref)	1 (ref)
1	0.96	0.73 to 1.26	0.97	0.73 to 1.28
2	0.80	0.56 to 1.15	0.79	0.54 to 1.15
3+	0.85	0.63 to 1.14	0.86	0.63 to 1.19
**IMD**				
Least deprived 10%	1 (ref)	1 (ref)	1 (ref)	1 (ref)
Less deprived 10-20	0.86	0.51 to 1.43	0.86	0.51 to 1.43
Less deprived 20-30%	1.03	0.63 to 1.68	1.02	0.63 to 1.67
Less deprived 30-40%	0.97	0.59 to 1.59	0.97	0.59 to 1.60
Less deprived 40-50%	1.13	0.70 to 1.82	1.12	0.69 to 1.81
More deprived 40-50%	0.89	0.50 to 1.57	0.89	0.50 to 1.57
More deprived 30-40%	1.01	0.60 to 1.70	1.00	0.59 to 1.69
More deprived 20-30%	1.68	1.07 to 2.64	1.68	1.07 to 2.63
More deprived 10-20%	1.59	1.01 to 2.49	1.59	1.01 to 2.49
Most deprived 10%	1.06	0.61 to 1.85	1.07	0.62 to 1.85
[a] Subhazard ratio				

For peer review only

Supplementary Table 10. Risk of revision following Trapeziectomy with LRTI
Multivariable regression analysis accounting for competing risk of mortality

LRTI	**Crude sHR**	**95% CI**	**Adjusted sHR[a]**	**95% CI**
**Male**	1.26	0.60 to 2.67	1.29	0.60 to 2.76
**Age**				
40-49 years	3.10	1.32 to 7.33	2.64	1.02 to 6.86
50-59 years	0.85	0.39 to 1.88	0.79	0.36 to 1.74
60-69 years	1 (ref)	1 (ref)	1 (ref)	1 (ref)
70-79 years	0.77	0.28 to 2.10	0.68	0.23 to 2.01
>80 years	0.77	0.10 to 5.83	0.81	0.10 to 6.28
**Charlson comorbidity index**				
0	1 (ref)	1 (ref)	1 (ref)	1 (ref)
1	1.14	0.55 to 2.34	1.04	0.50 to 2.13
2	0.79	0.29 to 2.09	0.85	0.31 to 2.31
3+	0.29	0.08 to 0.99	0.30	0.09 to 1.02
**IMD**				
Least deprived 10%	1 (ref)	1 (ref)	1 (ref)	1 (ref)
Less deprived 10-20%	0.40	0.04 to 4.39	0.39	0.04 to 4.26
Less deprived 20-30%	1.77	0.35 to 9.08	1.76	0.35 to 8.85
Less deprived 30-40%	1.39	0.25 to 7.64	1.34	0.24 to 7.33
Less deprived 40-50%	2.63	0.55 to 12.66	2.84	0.59 to 13.55
More deprived 40-50%	2.88	0.56 to 14.92	2.75	0.55 to 13.75
More deprived 30-40%	2.17	0.42 to 11.14	1.90	0.37 to 9.68
More deprived 20-30%	0.85	0.12 to 6.02	0.83	0.12 to 5.89
More deprived 10-20%	1.81	0.35 to 9.35	1.72	0.34 to 8.66
Most deprived 10%	1.52	0.22 to 10.73	1.22	0.17 to 8.92
[a] Subhazard ratio				

For peer review only

Supplementary Table 11. Risk of revision following BTOA Arthroplasty Multivariable regression analysis accounting for competing risk of mortality

Arthroplasty	**Crude sHR**	**95% CI**	**Adj sHR[a]**	**95% CI**
**Male**	0.66	0.31 to 1.40	0.72	0.33 to 1.60
**Age**				
40-49 years	0.92	0.32 to 2.69	1.07	0.37 to 3.10
50-59 years	0.75	0.37 to 1.48	0.80	0.40 to 1.59
60-69 years	1 (ref)	1 (ref)	1 (ref)	1 (ref)
70-79 years	0.83	0.36 to 1.89	0.76	0.31 to 1.84
>80 years	1.63	0.37 to 7.13	1.41	0.28 to 7.25
**Charlson comorbidity index**				
0	1 (ref)	1 (ref)	1 (ref)	1 (ref)
1	1.84	0.83 to 4.11	1.84	0.79 to 4.30
2	2.73	1.16 to 6.42	2.63	1.08 to 6.40
3+	2.47	1.12 to 5.43	2.14	0.88 to 5.24
**IMD**				
Least deprived 10%	1 (ref)	1 (ref)	1 (ref)	1 (ref)
Less deprived 10-20	1.77	0.45 to 6.89	1.71	0.44 to 6.62
Less deprived 20-30%	1.61	0.38 to 6.78	1.44	0.34 to 6.18
Less deprived 30-40%	3.23	0.87 to 12.00	2.96	0.78 to 11.22
Less deprived 40-50%	1.98	0.48 to 8.24	1.47	0.33 to 6.53
More deprived 40-50%	3.02	0.76 to 12.02	2.72	0.68 to 10.91
More deprived 30-40%	1.58	0.36 to 7.04	1.35	0.30 to 6.02
More deprived 20-30%	1.37	0.31 to 6.06	1.31	0.28 to 6.18
More deprived 10-20%	0.65	0.11 to 3.88	0.60	0.10 to 3.59
Most deprived 10%	1.91	0.42 to 8.65	1.22	0.24 to 6.30

[a] Subhazard ratio

For peer review only

Supplementary Table 12. Risk of revision following BTOA Arthrodesis Multivariable regression analysis accounting for competing risk of mortality

Arthrodesis	**Crude sHR**	**95% CI**	**Adj sHR[a]**	**95% CI**
**Male**	0.71	0.42 to 1.21	0.72	0.42 to 1.25
**Age**				
40-49 years	0.74	0.33 to 1.64	0.70	0.31 to 1.54
50-59 years	1 (ref)	1 (ref)	1 (ref)	1 (ref)
60-69 years	0.71	0.38 to 1.31	0.71	0.38 to 1.34
70-79 years	0.72	0.31 to 1.67	0.81	0.32 to 2.05
>80 years	0.51	0.07 to 3.72	0.54	0.08 to 3.86
**Charlson comorbidity index**				
0	1 (ref)	1 (ref)	1 (ref)	1 (ref)
1	2.07	1.10 to 3.88	2.18	1.16 to 4.10
2	1.36	0.60 to 3.12	1.48	0.62 to 3.55
3+	1.04	0.47 to 2.15	1.05	0.46 to 2.41
**IMD**				
Least deprived 10%	1 (ref)	1 (ref)	1 (ref)	1 (ref)
Less deprived 10-20	5.18	0.64 to 42.24	5.42	0.66 to 44.82
Less deprived 20-30%	4.06	0.49 to 33.89	4.16	0.49 to 35.07
Less deprived 30-40%	2.07	0.21 to 20.01	2.03	0.21 to 19.37
Less deprived 40-50%	4.09	0.49 to 34.21	3.93	0.46 to 33.33
More deprived 40-50%	1.69	0.15 to 18.84	1.62	0.14 to 18.29
More deprived 30-40%	6.59	0.83 to 52.31	6.50	0.79 to 53.61
More deprived 20-30%	10.00	1.30 to 76.46	9.77	1.26 to 75.72
More deprived 10-20%	6.05	0.76 to 48.20	5.92	0.72 to 49.01
Most deprived 10%	1.00	0.06 to 16.29	0.94	0.06 to 15.41

[a] Subhazard ratio

For peer review only

Supplementary Figures 1-5 Kaplan Meier plots of risk of further procedure following

1 Simple Trapeziectomy

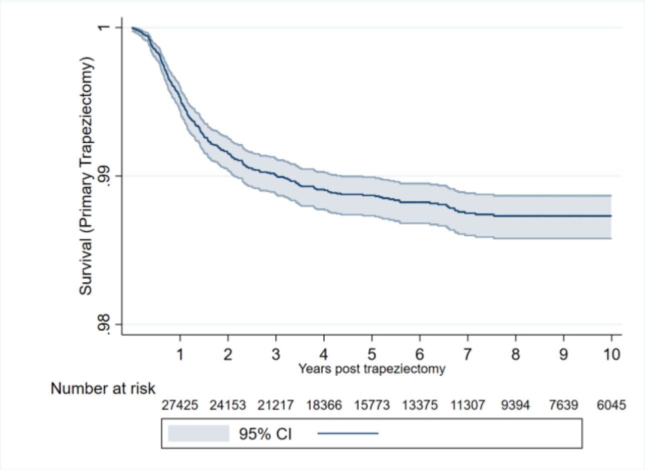

2 Trapeziectomy with LRTI

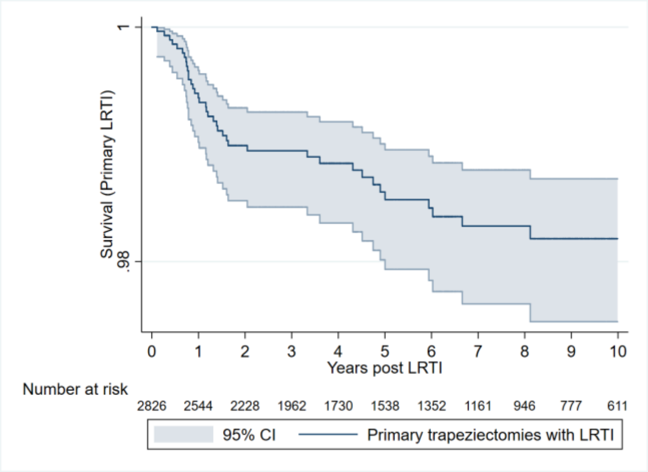

3 BTOA Arthroplasty

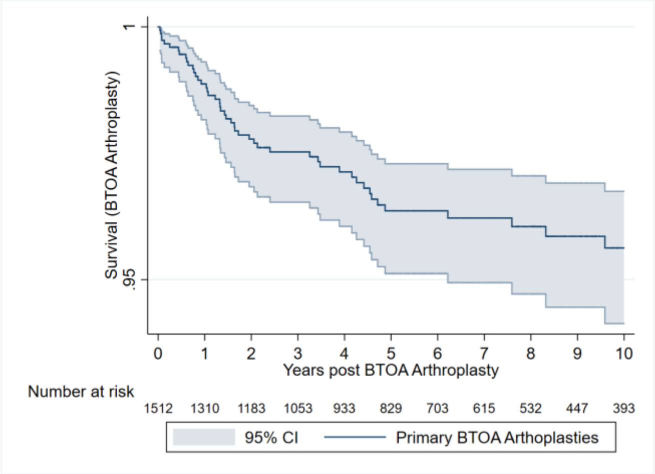

4 BTOA Arthrodesis

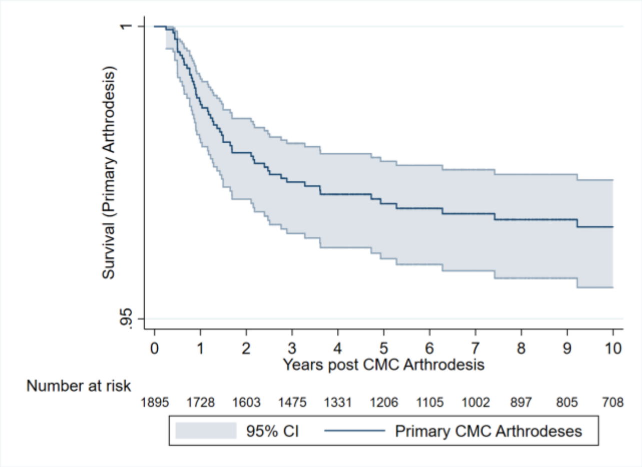

5 Partial Trapeziectomy

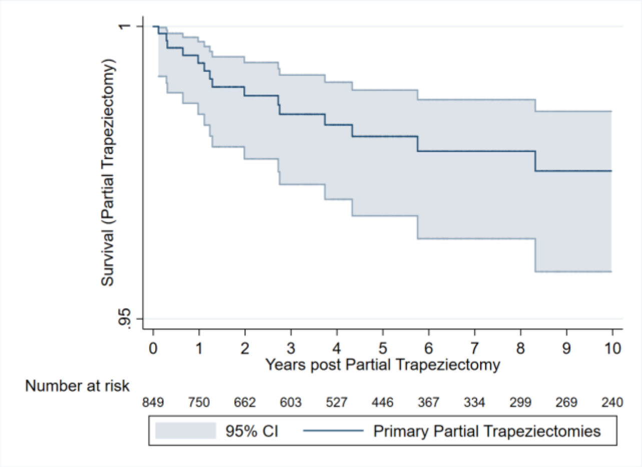

STROBE Statement—Checklist of items that should be included in reports of ***cohort studies***

	Item No	Recommendation	Page No
**Title and abstract**	1	(*a*) Indicate the study's design with a commonly used term in the title or the abstract<br>(*b*) Provide in the abstract an informative and balanced summary of what was done and what was found	1-2
**Introduction**			
Background/rationale	2	Explain the scientific background and rationale for the investigation being reported	4
Objectives	3	State specific objectives, including any prespecified hypotheses	4
**Methods**			
Study design	4	Present key elements of study design early in the paper	4-5
Setting	5	Describe the setting, locations, and relevant dates, including periods of recruitment, exposure, follow-up, and data collection	*4*
Participants	6	(*a*) Give the eligibility criteria, and the sources and methods of selection of participants. Describe methods of follow-up<br>(*b*) For matched studies, give matching criteria and number of exposed and unexposed	4-5
Variables	7	Clearly define all outcomes, exposures, predictors, potential confounders, and effect modifiers. Give diagnostic criteria, if applicable	5-6
Data sources/ measurement	8*	For each variable of interest, give sources of data and details of methods of assessment (measurement). Describe comparability of assessment methods if there is more than one group	4 & 5
Bias	9	Describe any efforts to address potential sources of bias	5
Study size	10	Explain how the study size was arrived at	4
Quantitative variables	11	Explain how quantitative variables were handled in the analyses. If applicable, describe which groupings were chosen and why	5-6
Statistical methods	12	(*a*) Describe all statistical methods, including those used to control for confounding<br>(*b*) Describe any methods used to examine subgroups and interactions<br>(*c*) Explain how missing data were addressed<br>(*d*) If applicable, explain how loss to follow-up was addressed<br>(*e*) Describe any sensitivity analyses	5-6
**Results**			
Participants	13*	(a) Report numbers of individuals at each stage of study—eg numbers potentially eligible, examined for eligibility, confirmed eligible, included in the study, completing follow-up, and analysed<br>(b) Give reasons for non-participation at each stage<br>(c) Consider use of a flow diagram	7
Descriptive data	14*	(a) Give characteristics of study participants (eg demographic, clinical, social) and information on exposures and potential confounders<br>(b) Indicate number of participants with missing data for each variable of interest<br>(c) Summarise follow-up time (eg, average and total amount)	7 + supp
Outcome data	15*	Report numbers of outcome events or summary measures over time	8 + supp

For peer review only

For peer review only - http://bmjopen.bmj.com/site/about/guidelines.xhtml

Main results	16	(*a*) Give unadjusted estimates and, if applicable, confounder-adjusted estimates and their precision (eg, 95% confidence interval). Make clear which confounders were adjusted for and why they were included<br>(*b*) Report category boundaries when continuous variables were categorized<br>(*c*) If relevant, consider translating estimates of relative risk into absolute risk for a meaningful time period	7-9
Other analyses	17	Report other analyses done—eg analyses of subgroups and interactions, and sensitivity analyses	supplementary
**Discussion**			
Key results	18	Summarise key results with reference to study objectives	10
Limitations	19	Discuss limitations of the study, taking into account sources of potential bias or imprecision. Discuss both direction and magnitude of any potential bias	11
Interpretation	20	Give a cautious overall interpretation of results considering objectives, limitations, multiplicity of analyses, results from similar studies, and other relevant evidence	10
Generalisability	21	Discuss the generalisability (external validity) of the study results	10
**Other information**			
Funding	22	Give the source of funding and the role of the funders for the present study and, if applicable, for the original study on which the present article is based	12

*Give information separately for exposed and unexposed groups.

Note: An Explanation and Elaboration article discusses each checklist item and gives methodological background and published examples of transparent reporting. The STROBE checklist is best used in conjunction with this article (freely available on the Web sites of PLoS Medicine at http://www.plosmedicine.org/, Annals of Internal Medicine at http://www.annals.org/, and Epidemiology at http://www.epidem.com/). Information on the STROBE Initiative is available at http://www.strobe-statement.org.

For peer review only

For peer review only - http://bmjopen.bmj.com/site/about/guidelines.xhtml
